# New proapoptotic chemotherapeutic agents based on the quinolone-3-carboxamide scaffold acting by VEGFR-2 inhibition

**DOI:** 10.1038/s41598-023-38264-w

**Published:** 2023-07-13

**Authors:** Zeinab S. El-Fakharany, Yassin M. Nissan, Nada K. Sedky, Reem K. Arafa, Sahar M. Abou-Seri

**Affiliations:** 1 Department of Pharmaceutical Chemistry, Faculty of Pharmacy, October University for Modern Sciences and Arts, Cairo, 12451 Egypt; 2 Department of Pharmaceutical Chemistry, Faculty of Pharmacy, Cairo University, Kasr El-Aini Street, Cairo, 11562 Egypt; 3 Department of Biochemistry, School of Life and Medical Sciences, University of Hertfordshire Hosted By Global Academic Foundation, New Administrative Capital, Cairo, Egypt; 4 Biomedical Sciences Program, University of Science and Technology, Zewail City of Science and Technology, October Gardens, 6th of October City, Giza, 12578 Egypt; 5 Drug Design and Discovery Lab, Zewail City of Science and Technology, Cairo, 12578 Egypt

**Keywords:** Biochemistry, Cancer, Drug discovery

## Abstract

In the current study, we designed and synthesized a series of new quinoline derivatives **10a-p** as antiproliferative agents targeting cancer through inhibition of VEGFR-2. Preliminary molecular docking to assess the interactions of the designed derivatives with the binding site of VEGFR-2 (PDB code: 4ASD) displayed binding poses and interactions comparable to sorafenib. The synthesized compounds exhibited VEGFR-2 inhibitory activity with IC_50_ ranging from 36 nM to 2.23 μM compared to sorafenib (IC_50_ = 45 nM), where derivative **10i** was the most potent. Additionally, the synthesized derivatives were evaluated in vitro for their cytotoxic activity against HepG2 cancer cell line. Seven compounds **10a**, **10c**, **10d**, **10e**, **10i**, **10n** and **10o** (IC_50_ = 4.60, 4.14, 1.07, 0.88, 1.60, 2.88 and 2.76 μM respectively) displayed better antiproliferative activity than sorafenib (IC_50_ = 8.38 μM). Compound **10i** was tested against Transformed Human Liver Epithelial-2 normal cell line (THLE-2) to evaluate its selective cytotoxicity. Furthermore, **10i**, as a potent representative of the series, was assayed for its apoptotic activity and cell cycle kinetics’ influence on HepG2, its effects on the gene expression of VEGFR-2, and protein expression of the apoptotic markers Caspase-7 and Bax. Compound **10i** proved to have a potential role in apoptosis by causing significant increase in the early and late apoptotic quartiles, a remarkable activity in elevating the relative protein expression of Bax and Caspase-7 and a significant reduction of VEGFR-2 gene expression. Collectively, the obtained results indicate that compound **10i** has a promising potential as a lead compound for the development of new anticancer agents.

## Introduction

Cancer is considered the second cause of death globally^1^. The quest for new anticancer drugs is still a demand for the globe. Several anticancer agents are being used despite their side effects and emerging resistance. The urge for developing new agents for better efficacy with fewer adverse effects can be explored through targeting kinases as they are variably overexpressed in various types of cancers. One such important kinase is the vascular endothelial growth factor receptor-2 (VEGFR-2) due to its major role in angiogenesis, the process of formation of new blood vessels used for oxygen and nutrients supply to the cells, which is a vital step required for cancer growth and metastasis^2^. This process is extensively noticed in most of the solid tumors due to their significant higher consumption of glucose, oxygen as well as other nutrients because of their rapid growth compared to the normal tissues^3^. It is reported that inhibiting tumor angiogenesis potentiates the effect of other therapeutic options as chemotherapy and radiotherapy, suggesting that agents which act on VEGF or its receptors can be co-administered with conventional therapy to achieve maximum effectiveness^4^. Even though there are several small-molecule VEGFR-2 kinase inhibitors approved by the Food and Drug Administration (FDA) for clinical use, there are several concerns limiting their use, such as drug resistance and side effects (cardiovascular, hyperparathyroidism and kidney injury)^5,6^. Hence, discovering novel inhibitors with satisfactory outcomes is still in a great demand.

The quinoline ring system has long been identified as a versatile nucleus in the design of biologically active agents as many quinoline derivatives exhibit different pharmacological activities as antibacterial^7^, antifungal^8^, antipsychotic^9^, antimalarial^10^, local anesthetic^11^, and anticancer^12^.

**Figure 1:**
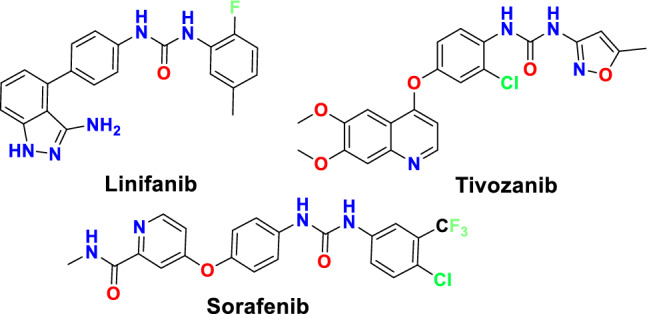
Structures of FDA approved VEGFR-2 inhibitor drugs featuring bi-aryl moiety.

Additionally, it was found that the bi-aryl urea moiety is widely used as a key structural fragment especially in type II kinase inhibitors for binding through hydrogen bonds and hydrophobic interaction. It also serves as a linker between the hinge-binding moiety and the lipophilic part of the molecule that would fit in the hydrophobic pocket present in the inactive form of the protein kinases (the DFG-out pocket). The urea linker was reported to interact with two conserved residues: a glutamic acid present in the αC-helix in addition to the aspartic acid in the DFG motif. Therefore, several bi-aryl urea containing compounds were synthesized and successively approved as VEGFR-2 inhibitors, such as sorafenib, linifanib and tivozanib (Fig. 1)^13,14^. These drugs are classified as type II inhibitors as they target the protein in the DFG-out conformation, where heteroaromatic scaffold occupies the adenine pocket and interacts with the hinge region, while the bi-aryl urea moiety is oriented to fit in a hydrophobic pocket near the ATP binding site and the gatekeeper residue, that was created upon the displacement of the DFG loop^14^. The urea linker of these inhibitors is reported to be involved in key H-bonding interactions with Glu885 and Asp1046 amino acids^12,15^.

**Figure 2:**
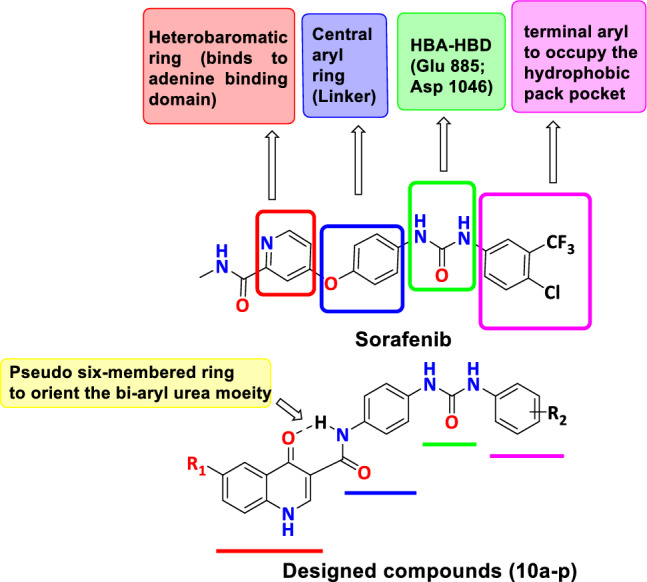
Design strategy of the target quinolone-3-carboxamide based VEGFR-2 inhibitors **10a-p**.

In continuation to our work in discovering new anticancer agents in general and VEGFR-2 inhibitors in particular^16–21^ it deemed of interest to explore new chemotypes for their VEGFR-2 inhibition-mediated anticancer potential. Thus, based on studying the structure activity relationships (SAR) of several VEGFR-2 inhibitors, in addition to analysis of the binding interactions of sorafenib and tivozanib as VEGFR-2 inhibitors, this project describes the design and synthesis of a series of new quinolone-3-carboxamide derivatives **10a-p** combined with a bi-aryl urea side chain for better binding inside the active site. In our design, for targeting the ATP binding site, the quinolone ring system featuring different substituents at position 6 was used, then a 3-amide function was used as a linker to connect the bi-aryl urea moiety. Noting that, the designed quinolone-3-carboxamide nucleus may form a pseudo six-membered ring through an intramolecular hydrogen bonding between the carbonyl group of the quinolone nucleus and the amide NH which may orient the bi-aryl urea moiety for better interaction with the key amino acids (Glu 885 and Asp 1046) and the hydrophobic pocket within the VEGFR-2 active site, thus resulting in high affinity. Finally, the adopted molecular manipulations involved decorating the terminal phenyl ring with different groups of various electronic and lipophilic characters to improve interaction with the hydrophobic back pocket of VEGFR-2 and gain an insight about the SAR of the designed compounds (Fig. 2).

The synthesized compounds were screened for their VEGFR-2 inhibitory activity and cytotoxic effect against hepatocellular carcinoma HepG2 cell line. In addition, selective cytotoxicity on normal liver cells was assessed for the most potent compound. Moreover, the proapoptotic activity, cell cycle alteration and gene and protein expression changes affected by treatment of HepG2 cells with the most active compound, as a potent representative of this class of compounds, were studied. Finally, in silico studies including molecular modelling for the target compounds was performed to elucidate their binding interactions within the active site of VEGFR-2 as well as pharmacokinetics properties predictions for the most active compound.

## Results and discussion

### Chemistry

**Figure 3:**
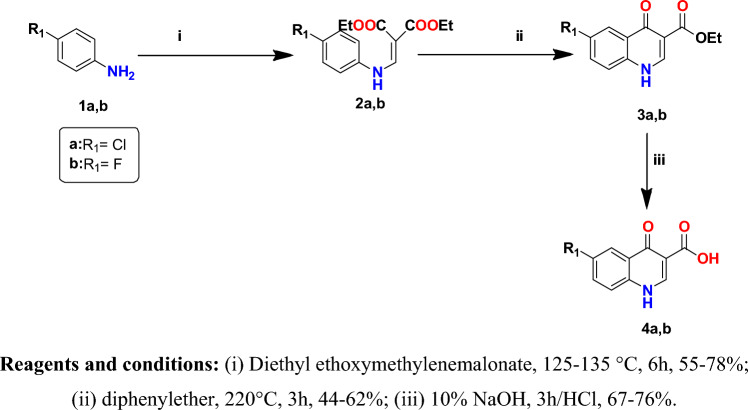
Synthesis of 6-substituted 4-quinolone-3-carboxylic acid derivatives **4a,b**.

**Figure 4:**
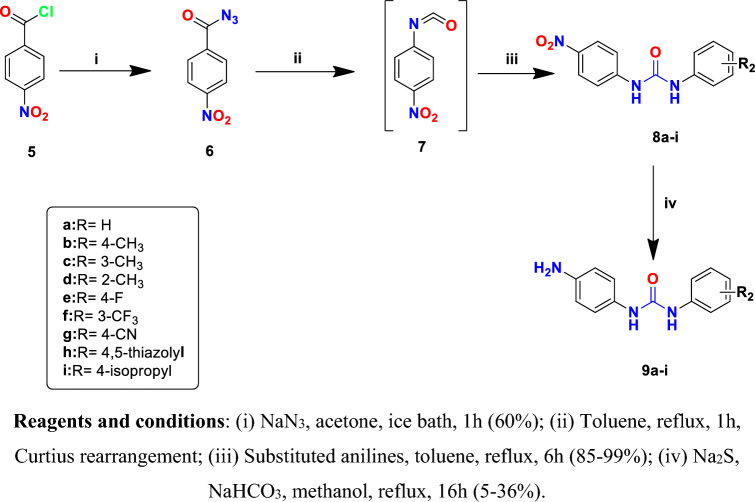
Synthesis of 1-(4-aminophenyl)-3-arylurea derivatives **9a-i**.

**Figure 5:**
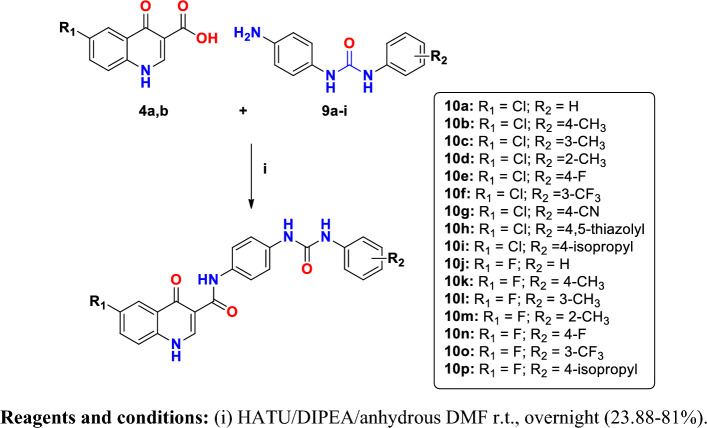
Synthesis of 6-Substituted 4-quinolone-3-carboxamide derivatives (**10a-p**).

This research work involves the synthesis of a series of novel 4-quinolone-3-carboxamide derivatives **10a-p**. The synthetic pathways used to obtain the new targeted compounds are represented in Figs. 3, 4 and 5. Figure 3 illustrates the synthesis of 6-substituted 4-quinolone-3-carboxylic acid derivatives (**4a** and **4b**) as starting materials. Synthesis of the quinolone ring was accomplished via the Gould-Jacobs reaction; where in the first step substituted anilines **1a,b** were condensed with diethyl ethoxymethylenemalonate to provide diethyl 2-((phenylamino)methylene)malonate derivatives **2a,b**^22–24^. Gould-Jacobs cyclization of **2a,b** in diphenyl ether produced the 4-oxo-1,4-dihydroquinoline-3-carboxylate ethyl esters **3a,b**^22–24^. The 4-oxo-1,4-dihydroquinoline-3-carboxylic acid derivatives **4a,b** were prepared by base hydrolysis of their ester analogs **3a,b** upon reflux with 1 M NaOH followed by acidification with diluted HCl to liberate the free acid derivatives **4a,b**^25^. The structures of compounds **4a,b** were confirmed by ^1^HNMR spectroscopy^23,26^.

Figure 4 summarizes the synthesis of 1-(4-aminophenyl) urea derivatives **9a–i** as starting material. Starting with the reaction of 4-nitrobenzoyl chloride **5** with sodium azide to form 4-nitrobenzoyl azide **6**^27^. This was followed by heating azide **6** in dry toluene to affect Curtius rearrangement and produce the corresponding isocyanate **7**, which was then reacted with different anilines to produce the required urea derivatives **8a-i**. The obtained 1-(4-nitrophenyl)-3-arylurea derivatives (**8a-i**) were converted to their reduced amino derivatives **9a-i** using sodium hydrogen sulfide in aqueous methanol. The solution containing sodium hydrogen sulfide was freshly prepared by adding sodium bicarbonate to an aqueous solution of sodium sulfide followed by the addition of methanol^28^. All the synthesized 1-(4-aminophenyl)-3-arylurea derivatives **9a-9i** were confirmed by ^1^H-NMR analyses where all protons were seen according to the expected chemical shift with characteristic new peak representing the two D_2_O exchangeable protons of the newly formed -NH_2_ group at the range of 4.74–4.79 ppm.

Synthesis of the target 6-substituted-4-quinolone-3-carboxamide derivatives **10a**-**p** is illustrated in Fig. 5. In this work, the final step comprises formation of the amide bond through direct coupling of the obtained quinolinone-3-carboxylic acid derivatives **4a,b** and the 1-(4-aminophenyl)-3-arylurea derivatives **9a-i** using the coupling agent HATU and DIPEA as a base to yield compounds **10a-p**^29–32^. Structures of the target compounds was confirmed by spectral and elemental analyses. The ^1^HNMR spectra of compounds **10a-p** revealed the disappearance of D_2_O exchangeable signal at 4.74–4.79 ppm of amino protons in **9a-i** and the appearance of D_2_O exchangeable signal for the newly formed CONH at 12.19–12.47 ppm thus assuring the formation of amide bond. Besides, the two singlet signals assigned for the urea NH groups were detected at 7.88–9.04 ppm.

### Biological screening

#### Enzyme inhibition assay versus VEGFR-2

All synthesized compounds **10a-p** were in-vitro evaluated for their VEGFR-2 inhibition activity using sorafenib as a reference drug. The kinase assay was performed using Kinase-Glo MAX Luminescence assay kit from BPS Bioscience (Catalog number: 40325). The results are presented as IC_50_ values (nM) ± SE and listed in Table 1. Most of the tested compounds displayed potent to moderate inhibitory VEGFR-2 activity (36–578 nM) except for compounds **10j**, **10 h** and **10k** which displayed low inhibitory activity (IC_50_ = 937–2226 nM). It is worthy to note that compounds **10i** and **10o** displayed higher or comparable VEGFR-2 inhibitory activity to the reference drug sorafenib (IC_50_ = 36, 38 and 45 nM, respectively).

**Table 1:** Results of VEGFR-2 inhibitory activity of the final derivatives as IC_50_ in nM.


Compound	R_1_	R_2_	IC_50_ (nM)
**10a**	Cl	H	138 ± 0.01
**10b**	Cl	4-CH_3_	578 ± 0.03
**10c**	Cl	3-CH_3_	169 ± 0.01
**10d**	Cl	2-CH_3_	69 ± 0.02
**10e**	Cl	4-F	179 ± 0.01
**10f**	Cl	3-CF_3_	104.5 ± 0.005
**10g**	Cl	4-CN	1603 ± 0.06
**10h**	Cl	4,5-thiazolyl	937 ± 0.04
**10i**	Cl	4-isopropyl	36 ± 0.01
**10j**	F	H	340 ± 0.01
**10k**	F	4-CH_3_	2226 ± 0.09
**10l**	F	3-CH_3_	317 ± 0.01
**10m**	F	2-CH_3_	181 ± 0.01
**10n**	F	4-F	257 ± 0.01
**10o**	F	3-CF_3_	38 ± 0.02
**10p**	F	4-isopropyl	97 ± 0.01
**Sorafenib**	–	–	45 ± 0.02

*IC_50_ values are the average of 3 independent runs ± SE.

Close examination of the results illustrated in Table 1 revealed that the inhibitory potency of the tested compounds is affected by the substituent at the 6-positon on the quinolone ring as well as that substituent on the terminal phenyl ring.

In the present study, two major series were evaluated; the first one represents the 6-chloroquinolone derivatives (**10a**-**i**). The second one displays the 6-flouroquinolone derivatives (**10j**- **p**). Generally, the 6-chloroquinolone derivatives (**10a**-**i**) seemed to show higher inhibition for VEGFR-2 than the 6-flouroquinolone counterparts (**10j-p**). For example: the 6-chloroquinolone derivative with unsubstituted phenyl urea moiety **10a** (R_2_ = H) showed an IC_50_ = 138 nM compared to its 6-flouroquinolone analog **10j** with IC_50_ = 340 nM, Similarly, the *para-*methylphenyl derivative **10b** (R_2_ = 4-CH_3_) of the 6-chloroquinolone series has IC_50_ = 578 nM versus its 6-flouro counterpart **10k** with IC_50_ = 2226 nM. Thus, SAR analysis implies that the incorporation of chlorine atom with a relatively larger size and higher lipophilicity than fluorine at the 6-position of the quinolone ring was beneficial for the VEGFR-2 inhibitory activity.

Regarding the effect of nature and position of substituent on the terminal phenyl on the VEGFR-2 inhibitory activity, *para* substitution with a lipophilic electron withdrawing substituent such as fluorine in compounds **10e **(R_1_ = Cl, R_2_ = 4-F, IC_50_ = 197 nM) and **10n** (R_1_ = F, R_2_ = 4-F, IC_50_ = 257 nM) resulted in comparable activity to those with unsubstituted phenyl **10a** (R_1_ = Cl, R_2_ = H, IC_50_ = 138 nM) and **10j** (IC_50_ = 340 nM). On the other hand, grafting a *para* hydrophilic electron withdrawing cyano group in compound **10g** (R_1_ = Cl, R_2_ = 4-CN, IC_50_ = 1603 nM) was detrimental to the inhibitory effect, this might be attributed to poor fitting of this derivative in the hydrophobic back pocket of the VEGFR-2 active site. Likewise, substitution with a small lipophilic electron donating moiety such as methyl group at the *para* position as in derivatives **10b** (R_1_ = Cl, R_2_ = 4-CH_3_, IC_50_ = 578 nM) and **10k** (R_1_ = F, R_2_ = 4-CH_3_, IC_50_ = 2226 nM) resulted in decreased activity when compared to the unsubstituted phenyl analogues **10a** and **10j**. Interestingly, attaching a branched isopropyl substituent in **10i** (R_1_ = Cl, R_2_ = 4-isopropyl, IC_50_ = 36 nM) and **10p** (R_1_ = F, R_2_ = 4-isopropyl, IC_50_ = 97 nM) resulted in more than 16 fold increase in activity compared to their *para* methyl substituted derivatives **10b** and **10k**.

Moving to the *meta* position at the terminal phenyl ring; addition of a methyl group in compounds **10c** (R_1_ = Cl, R_2_ = 3-CH_3_, IC_50_ = 169 nM) and **10l** (R_1_ = F, R_2_ = 3-CH_3_, IC_50_ = 317 nM) had no significant effect on potency when related to the unsubstituted phenyl derivatives **10a** (R_1_ = Cl, R_2_ = H, IC_50_ = 138 nM) and **10j** (R_1_ = F, R_2_ = H, IC_50_ = 340 nM), respectively. However, the more lipophilic electron withdrawing *meta*-trifluoromethyl counterparts **10f** (R_1_ = Cl, R_2_ = 3-CF_3_, IC_50_ = 104.5 nM) and **10o** (R_1_ = F, R_2_ = 3-CF_3_, IC_50_ = 38 nM) improved the inhibitory activity to yield the most potent derivative **10o** among the 6-flouroquinolone derivatives.

Noticeably, fusion of an extra heterocycle to the meta and para positions of the terminal phenyl, the benzothiazole derivative **10 h** (R_1_ = Cl, R_2_ = 4,5-thiazolyl), resulted in decreased activity by about 5.5-fold (IC_50_ = 937 nM).

Finally, positional changes of the substituent on the pendant phenyl affected the biological activity, this was observed through comparing the IC_50_ values of the methyl positional isomers **10b-d** and **10k-m**. The order of activity was *ortho* methyl substituted derivatives **10d** (R_1_ = Cl, R_2_ = 2-CH_3_, IC_50_ = 69 nM) and **10m** (R_1_ = F, R_2_ = 2-CH_3_, IC_50_ = 181 nM) > *meta* methyl substituted compounds **10c** (R_1_ = Cl, R_2_ = 3-CH_3_, IC_50_ = 169 nM) and **10l** (R_1_ = F, R_2_ = 3-CH_3_, IC_50_ = 317 nM) > *para* methyl substituted compounds **10b** (R_1_ = Cl, R_2_ = 4-CH_3_, IC_50_ = 578 nM) and **10k** (R_1_ = F, R_2_ = 4-CH_3_, IC_50_ = 2226 nM). The higher potency of the ortho substituted derivatives **10d** and **10m** may be attributed to volumetric and consequently conformational changes affecting the coplanarity in the molecule resulting in more favorable bioactive conformers^33^.

**Figure 6:**
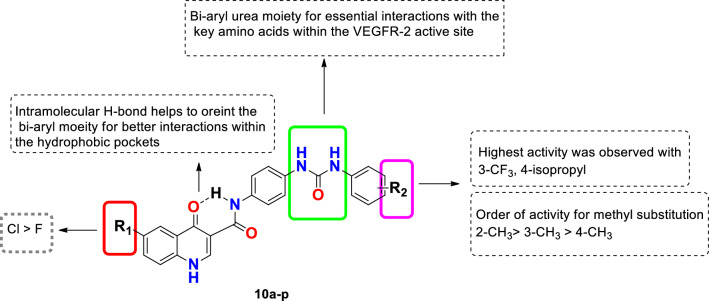
Summary of structure activity relationship findings for VEGFR-2 inhibitory activity.

In conclusion, among the most active derivatives in both series were the *meta* trifluoro methyl derivatives (**10f** and **10o**) and *para* isopropyl derivatives (**10i** and **10p**). This shows that insertion of an electron withdrawing or donating lipophilic group with considerable bulkiness at either *meta* or *para* position of the phenyl urea moiety resulted in improved inhibitory activity due to better filling of the hydrophobic region of the VEGFR-2 active site. The deduced SAR is summarized in Fig. 6. Based on these findings and compared to sorafenib (IC_50_ = 45 nM); we can suggest that compounds **10i** (IC_50_ = 36 nM) and **10o** (IC_50_ = 38 nM) could serve as suitable candidates for further investigation as VEGFR-2 inhibitors.

#### Cytotoxicity against hepatocellular carcinoma (HepG2)

The target compounds **10a-p** were also tested for their in-vitro anticancer activity against HepG2 tumor cell line using sorafenib as a reference drug and obtained results are shown in Table 2. This assay was performed by the means of 3-(4,5-dimethylthiazol-2-yl)-2,5-diphenyltetrazolium bromide (MTT) based in-vitro toxicological assay kit from SIGMA (catalog number: M-5655). VEGFR-2 overexpression is observed in different kinds of cancer such as hepatocellular carcinoma^34^. Therefore, HepG2 cell line has been selected for the cytotoxicity assessment of IC_50_ of the tested compounds **10a-p**. Moreover, liver cancer is considered to be the sixth most frequent malignancy and the fourth most prevalent cause of cancer-related deaths worldwide^35^.

**Table 2:** Results of cytotoxic activity screened against HepG2 cell line as IC_50_ in μM.

Compound No.	R_1_	R_2_	IC_50_ (μM)*
**10a**	Cl	H	4.60 ± 0.28
**10b**	Cl	4-CH_3_	50.83 ± 3.16
**10c**	Cl	3-CH_3_	4.14 ± 0.25
**10d**	Cl	2-CH_3_	1.07 ± 0.06
**10e**	Cl	4-F	0.88 ± 0.05
**10f**	Cl	3-CF_3_	62.02 ± 3.87
**10g**	Cl	4-CN	43.15 ± 2.59
**10h**	Cl	4,5-thiazolyl	14.94 ± 0.52
**10i**	Cl	4-isopropyl	1.60 ± 0.17
**10j**	F	H	41.91 ± 2.52
**10k**	F	4-CH_3_	34.21 ± 2.06
**10l**	F	3-CH_3_	14.18 ± 0.82
**10m**	F	2-CH_3_	13.01 ± 0.75
**10n**	F	4-F	2.88 ± 0.14
**10o**	F	3-CF_3_	2.76 ± 0.1
**10p**	F	4-isopropyl	8.14 ± 0.47
**Sorafenib**	–	–	8.38 ± 0.5

*IC_50_ values are the average of 3 independent runs ± SE.

Examination of the results of the cytotoxic activity of the synthesized derivatives against HepG2 tumor cell line showed anticancer activity with IC_50_ values ranging from 0.88 to 62.02 μM. Seven compounds **10a**, **10c**, **10d**, **10e**, **10i**, **10n** and **10o** (IC_50_ = 4.60, 4.14, 1.07, 0.88, 1.60, 2.88 and 2.76 μM, respectively) displayed better antiproliferative activity than sorafenib (IC_50_ = 8.38 μM). Furthermore, one derivative **10p** (R_1_ = F, R_2_ = 4-isopropyl, IC_50_ = 8.14 μM) exhibited comparable activity to sorafenib.

In accordance with the SAR analysis of VEGFR-2 inhibition of the synthesized derivatives, most of the 6-chloroquinolone derivatives reported better anticancer activity than their corresponding 6-flouroquinolone analogs. Also, substitution on the terminal phenyl ring with a *para* isopropyl group as in derivatives **10i** (R_1_ = Cl, R_2_ = 4-isopropyl, IC_50_ = 1.60 μM) and **10p** (R_1_ = F, R_2_ = 4-isopropyl, IC_50_ = 8.14 μM) showed potent anticancer activity as well as *2-*methyl substitution in derivatives **10d** (R_1_ = Cl, R_2_ = 2-CH_3_, IC_50_ = 1 0.07 μM) and **10m** (R_1_ = F, R_2_ = 2-CH_3_, IC_50_ = 13.01 μM) compared to sorafenib.

As an exception, compounds bearing *para* flouro substituent on the terminal phenyl ring **10e** (R_1_ = Cl, R_2_ = 4-F, IC_50_ = 0.88 μM) and **10n** (R_1_ = F, R_2_ = 4-F, IC_50_ = 2.88 μM) showed enhanced cytotoxic activity regardless of their moderate VEGFR-2 inhibitory potency.

In conclusion, the cytotoxic activity of most of the tested compounds agreed with SAR analysis of their VEGFR-2 inhibitory activities suggesting that the potent anticancer activity of the synthesized compounds might be attributed to their VEGFR-2 inhibitory activity.

#### Cytotoxicity screening against normal liver cell line (THLE-2)

Compounds **10i** and **10o** exhibited the best VEGFR-2 inhibitory activity (IC_50_ = 36 and 38 nM, respectively) with potent cytotoxic activity against HepG2 tumor cells (IC_50_ = 1.60 and 2.76 μM, respectively); consequently, they were subjected to cytotoxicity screening against Transformed Human Liver Epithelial-2 normal cell line (THLE-2) using Sorafenib as reference drug (Table 3). The selectivity index (SI) of the designed compounds was calculated as a ratio of CC_50_ against normal cell line (THLE-2) to IC_50_ on cancer cell line (HepG2) which reflects the selectivity of these compounds and accordingly their safety profile. Compounds **10i** and **10o** reported higher selectivity indices (SI = 13.13 and 5.52, respectively) than the reference drug sorafenib (SI = 2.20), indicating an acceptable cytotoxicity profile. Since compound **10i** displayed higher selectivity than **10 o**, it was selected for further studies.

**Table 3:** Results of the cytotoxic activity of compounds **10i**,**10o** and sorafenib against THLE-2 normal cell line as CC_50_ in μM and their calculated selectivity index (SI) for HepG2 cancer cell line.

Compound	CC_50_^a^	SI^b^
**10i**	21.00	13.13
**10o**	15.18	5.52
**Sorafenib**	18.49	2.20

^a^CC_50_: Cytotoxic activity against THLE-2 normal cell line in μM; measured in triplicates.

^b^SI: Selectivity index calculated as CC_50_/IC_50_.

#### Apoptosis assay

A hallmark of cancer cells is the loss of apoptotic control which allows cells to survive longer and gives more time for the accumulation of mutations which can increase invasiveness during tumor progression, stimulate angiogenesis, deregulate cell proliferation and interfere with differentiation^36^. Moreover, it was reported that, VEGFR-2 inhibition in cancer cells triggers apoptosis, which synergistically augments the anticancer effect^37^.

**Figure 7:**
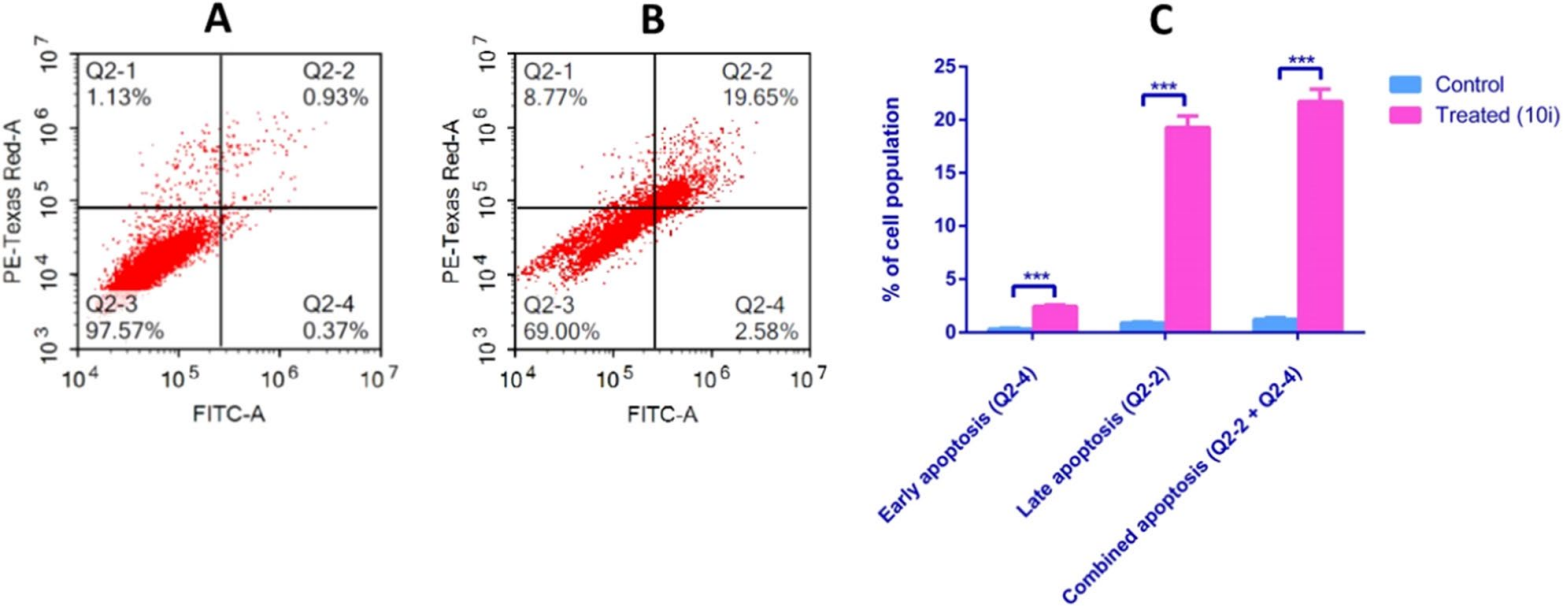
Apoptosis in HepG2 cells after 48 h exposure to compound **10i**. Cytograms presenting annexin-V/Propidium Iodide-stained untreated HepG2 cells as negative control (**A**), cells treated with compound **10i** (**B**), and representation of the apoptosis analysis results (**C**). Quadrant charts show Q2-1 (necrotic cells, AV-/PI +), Q2-2 (late apoptotic cells, AV + /PI +), Q2-3 (normal cells, AV-/PI-), Q2-4 (early apoptotic cells, AV + /PI-). Data represented is the average of triplicate experimental trials ± SD. *** refers to p-values ≤ 0.0005.

To explore the ability of most potent VEGFR-2 inhibitor **10i** to restore apoptosis, HepG2 cancer cells were treated with compound **10i** for 48 h at its IC_50_ concentration. Non-treated HepG2 cells were employed as the negative control and results are demonstrated in Table 4 and Fig. 7. The quinolone derivative **10i** caused a significant increase in early (2.43% cell population), late (19.28% cell population), and combined apoptosis (21.72% cell population) quartiles. Studies have also reported a similar, yet a little bit weaker apoptotic profile for the reference drug Sorafenib, whereby 2.17% cell population were observed in the early apoptotic phase, 4.2% in the late apoptotic phase and 6.37% in the combined apoptosis^38^. This in turn provides a robust support for compound **10i** ability to reimpose apoptosis in HepG2 and act as an anticancer agent.

**Table 4:** Apoptosis assay of HepG2 cells after treatment with the most potent compound **10i** for 48 h.

Apoptotic stage	Percent cell population*	
	Negative control	10i Treated
**Late apoptosis (Q2-2)**	0.91 ± 0.09	***19.28 ± 1.09
**Early apoptosis (Q2-4)**	0.32 ± 0.08	***2.43 ± 0.13
**Combined apoptosis (Q2-2 + Q2-4)**	1.23 ± 0.18	***21.72 ± 1.16

*The provided percent is the mean of triplicate independent runs ± SD.

***indicates a high significance where p-values ≤ 0.0005.

#### Cell cycle analysis

**Figure 8:**
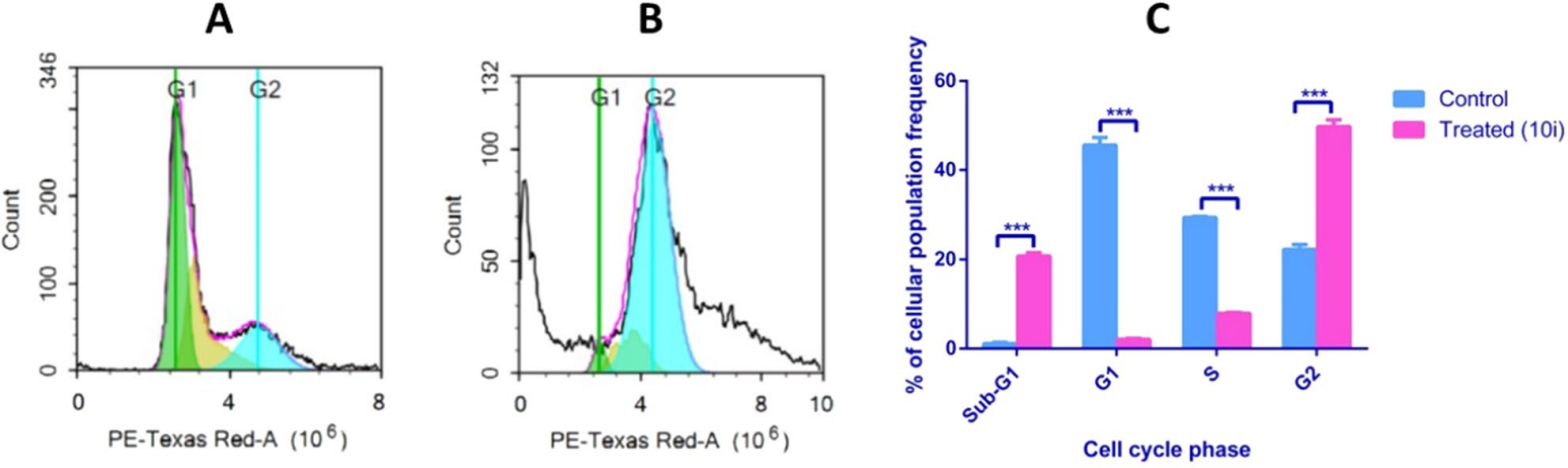
Cytograms showing the cell cycle analysis of A549 cells. (**A**) represents non treated HepG2 cells (control), (**B**) represents HepG2 cells after 48 h exposure to compound **10i** (treated), (**C**) a graph demonstrating the percentage of HepG2 cell population in different cell cycle phases. *** refers to p-values ≤ 0.0005.

DNA flow cytometry was used to analyze the cell cycle kinetics of HepG2 cancer cells when pre-treated for 48 h with compound **10i** at its IC_50_ concentration. Non-treated HepG2 cells were considered as negative control. The assay proved beneficial in determining the exact phases of the cell cycle that were affected with the target compound **10i** (Table 5, Fig. 8). In agreement with the apoptosis assay results, application of compound **10i** caused a profound sequestration of cells in Sub-G1(G0) indicating an apoptosis-enhancing activity^39^ as well as accumulation of cells at G2 phase, hence preventing progression of the cell cycle to the M-phase. Furthermore, the compound significantly decreased the frequency of cells in the S-phase thus interrupting the DNA synthesis. The compound also caused a remarkable decrease in the frequency of cells that pass to the G1 phase to continue with the cell cycle, so lowering down the progression of the cycle. Literature search also revealed great similarity in the cell cycle kinetics of HepG2 cells upon application of the reference drug Sorafenib at its IC_50_ for 48 h; Single treatment with sorafenib resulted in sequestration of cells in the PreG1 (or Sub-G1 (G0)), as well as cell growth arrest in G2/M phase^38^.

**Table 5:** Cell cycle analysis of HepG2 cells treated with compound **10i** compared to untreated HepG2 cells (Control).

Cell cycle phases	Control	**10i**
**Freq Sub-G1**	1.13 ± 0.2987	***20.80 ± 0.7102
**Freq G1**	45.62 ± 1.73	***2.067 ± 0.189
**Freq S**	29.35 ± 0.2937	***7.920 ± 0.2117
**Freq G2**	22.26 ± 1.139	***49.80 ± 1.466

Data are presented as the average of triplicate experiments ± SD. Freq refers to the frequency of cells at different phases of the cell cycle (Sub-G1, G1, S and G2 phases). *** refers to p-values ≤ 0.0005.

#### Real-time PCR analysis (RT-qPCR)

**Figure 9:**
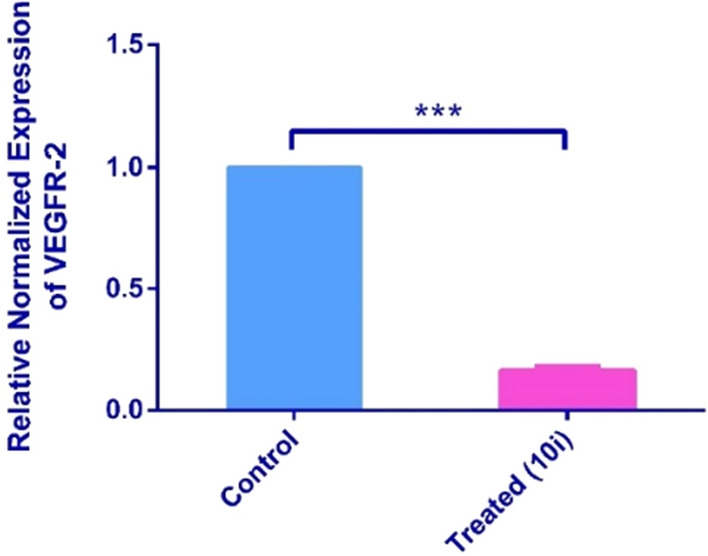
Real-time PCR analysis data showing the relative normalized expression of VEGFR-2 after exposure of HepG2 cells to compound **10i**. Each value represents three replicates. β-actin gene was used as the reference gene for normalization and to calculate the relative expression based on 2^−ΔΔCt^ method^41^.

RT-qPCR was performed to assess the relative normalized gene expression of VEGFR-2 gene in HepG2 cancer cells when pre-treated for 48 h with compound **10i** at its IC_50_ concentration utilizing non-treated HepG2 cells as negative control. Results displayed a remarkable decline in VEGFR-2 expression upon addition of our chemotherapeutic compound **10i** whereby it decreased the VEGFR-2 expression by 84% (8.4-fold) compared to untreated cells (Fig. 9). Setting against the reference drug Sorafenib, our compound (**10i**) possesses a surplus activity on HepG2 cells. For instance, Sorafenib is known for its ability to downregulate VEGFR-2 in certain types of cancer cells including A549 and Hela but not HepG2 cells as sorafenib caused upregulation of VEGFR-2 when introduced to HepG2 cells^40^. Therefore, **10i** is suggested to be more advantageous to hepatocellular carcinoma where it caused a remarkable downregulation in VEGFR-2 expression in HepG2 cells.

#### Western blotting

The probable mechanistic effect of **10i** has been further investigated on the two apoptotic markers, Bax and Caspase-7. Bax and Caspase-7 were selected as early and late apoptotic markers, respectively^42,43^.

**Figure 10:**
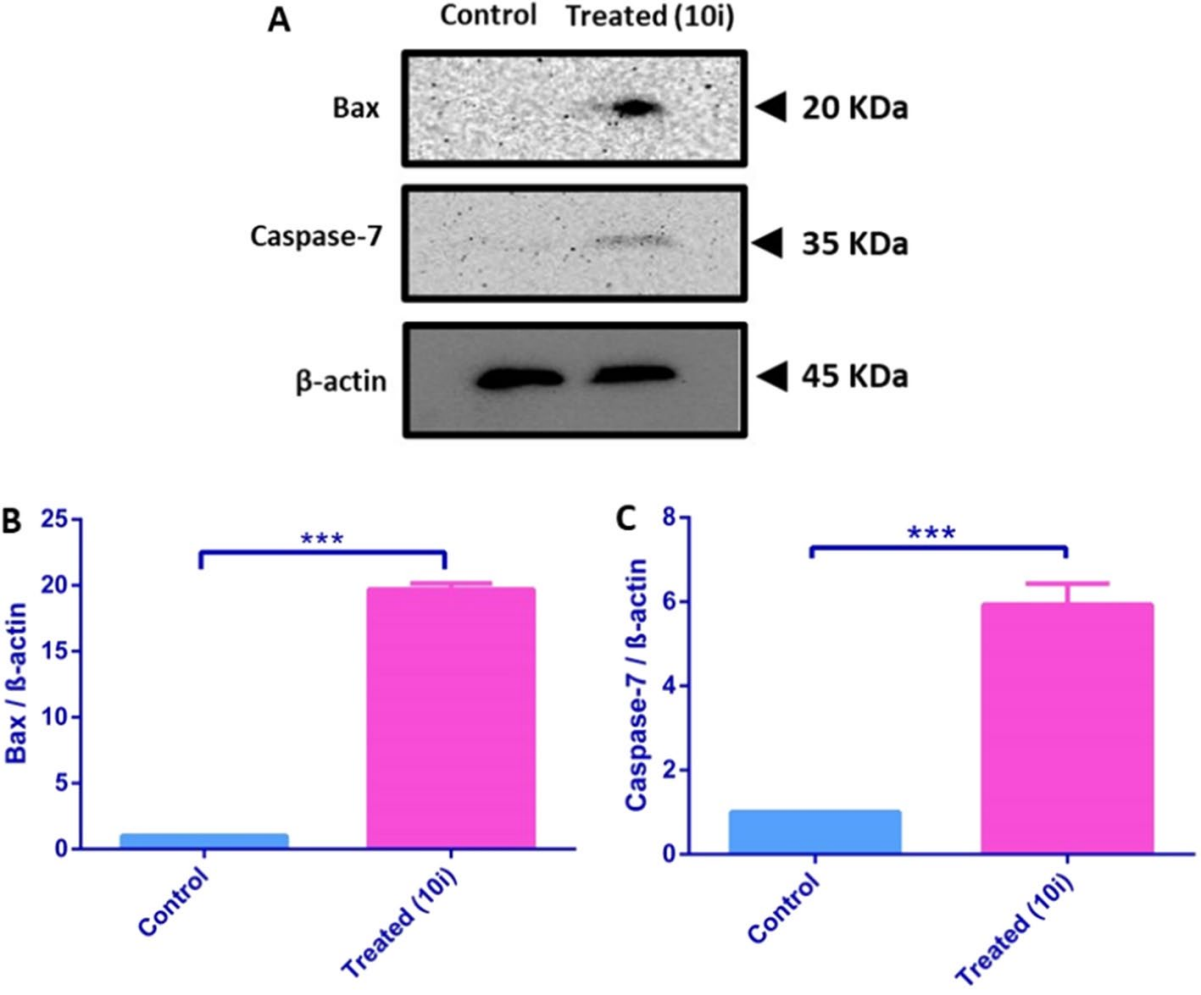
(**A**) Representative western blots for HepG2 levels of Bax and Caspase-7 in both compound (**10i**)-treated and untreated groups. (**B**) A bar chart representation for the relative protein expression of Bax normalized to β-actin levels. (**C**) A bar chart representation for the relative protein expression of Caspase-7 normalized to β-actin levels. Data are presented as mean ± SD (n = 3). Statistical analysis was carried out using one-way ANOVA followed by Tukey–Kramer post hoc test. *** indicates significant difference vs. control group at p < 0.001.

Western blotting was performed to assess the relative normalized protein expression of the apoptotic proteins Bax and Caspase-7 in HepG2 cancer cells when pre-treated for 48 h with compound **10i** at its IC_50_ concentration in comparison to non-treated HepG2 cells. A significant elevation in the protein expression of both Bax and Caspase-7 was demonstrated in treated cells where **10i** significantly induced protein expression of Bax and Caspase-7 up to 20-folds and 6-folds, respectively compared to the negative control group (Fig. 10). A previous study by Li et al*.* reported similar findings for the reference drug sorafenib regarding its ability to significantly upregulate the expression of caspase-7 in HepG2 cells, however, no significant upregulation of the pro-apoptotic marker, Bax was reported^44^. The current findings provide strong evidence for the positive apoptotic activity of compound **10i** in HepG2 cells that accompanies its inhibitory activity on cell proliferation.

### Computational study

#### Molecular docking

**Figure 11:**
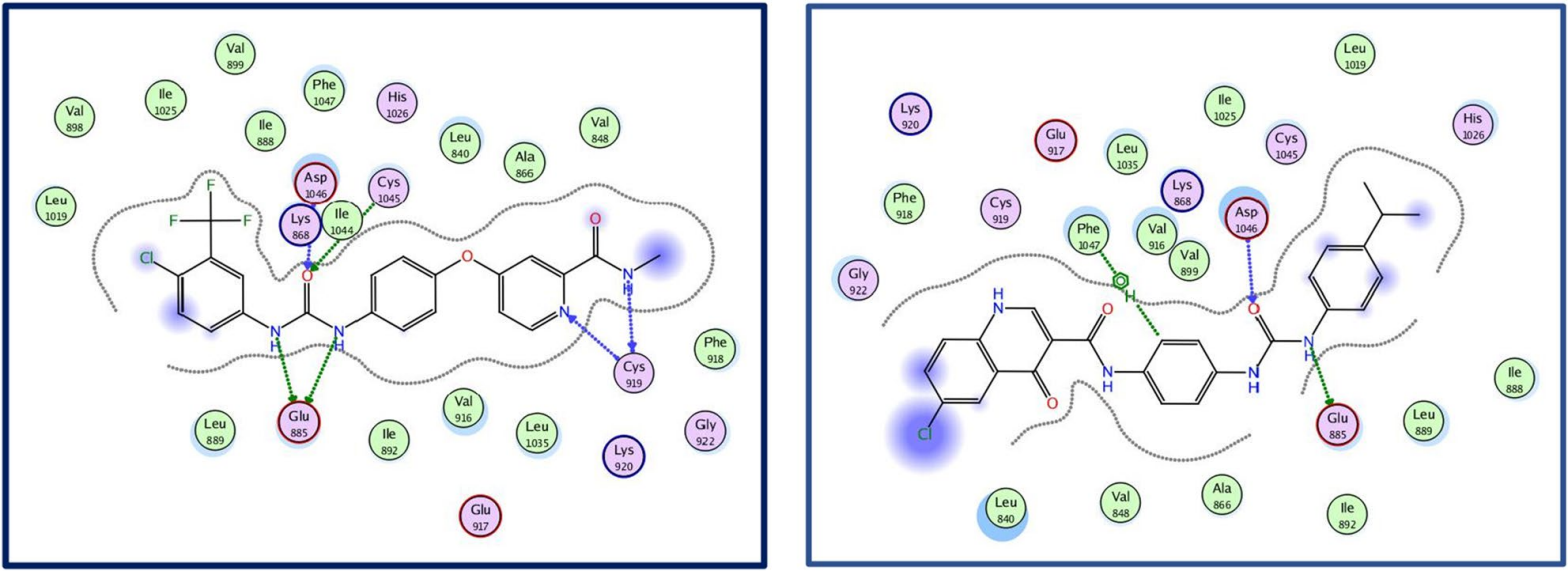
Left panel: 2D interactions of Sorafenib; right panel: 2D interactions of compound **10i** (PDB ID: 4ASD).

Molecular modeling is a computational method used to simulate the behavior of molecules within the active site of biological targets. Owing to the enormous increase in the available structural data for proteins as well as the major advances in the field of computational techniques and hardware, highly accurate docking methodologies were developed to aid in the multistep process of drug design^45,46^. Docking studies were performed to fit the designed molecules into the active site of the VEGFR-2 (PDB code: 4ASD) to predict their plausible binding mode inside the active site residues using the software “Molecular Operating Environment” (MOE) version 2019.01. Docking was performed employing alpha triangle placement method, poses were prioritized based on affinity London dG scoring and refinement of the results was done using forcefield. Docking scores of the best poses were recorded (Table 6) as well as their interactions within the active site (data not shown). All synthesized compounds were found to fit well within the active site of VEGFR-2 (PDB ID: 4ASD) with similar binding modes to the reference molecule sorafenib. Docking scores of this series members were in the range of −9.96 to −8.40 kcal/mol being better than the docking score of the reference drug sorafenib (S score = −7.79 kcal/mol). In our design, a quinoline carboxamide nucleus was used in which the amide group at the 3-position of the quinoline nucleus formed a pseudo six membered ring with the carbonyl group at position 4; this helped with the correct orientation of the novel derivatives across the allosteric channel towards the hydrophobic pocket within the pocket of VEGFR-2. Moreover, all compounds displayed a good correlation between the docking results and the VEGFR-2 inhibitory activity. As an example, docking results of compound **10i** are presented herein. **10i** showed the second-best docking score −9.72 kcal/mol and formed key interactions with the active site amino acids of VEGFR-2 comparable to sorafenib where **10i**’s urea NH formed a hydrogen bond with Glu 885, the urea C=O formed another H bond with Asp 1046 and the central phenyl ring was involved in a pi-H interaction with Phe 2047 (Fig. 11). The terminal phenyl ring bearing the lipophilic isopropyl group fitted into the hydrophobic back pocket lined with the hydrophobic side chains of Ile888, Leu889, Ile892, Leu1019 and Ile1044. Thus, the increased binding affinity of **10i** could be attributed to increase in hydrophobic interaction with the back pocket. These results are in line with the potent VEGFR-2 inhibitory activity of **10i **(IC_50_ = 36 nM), thus, proving our hypothesis and supporting our design strategy.

**Table 6:** Docking scores of the designed derivatives within the pocket of VEGFR-2.

Compound	Docking score (Kcal/mol)
**10a**	−8.80
**10b**	−9.02
**10c**	−9.30
**10d**	−8.78
**10e**	−8.79
**10f**	−9.28
**10g**	−9.18
**10h**	−9.47
**10i**	−9.72
**10j**	−8.40
**10k**	−8.97
**10l**	−9.21
**10m**	−8.73
**10n**	−8.70
**10o**	−9.17
**10p**	−9.96
**Sorafenib**	−7.79

#### In silico pharmacokinetic prediction

**Figure 12:**
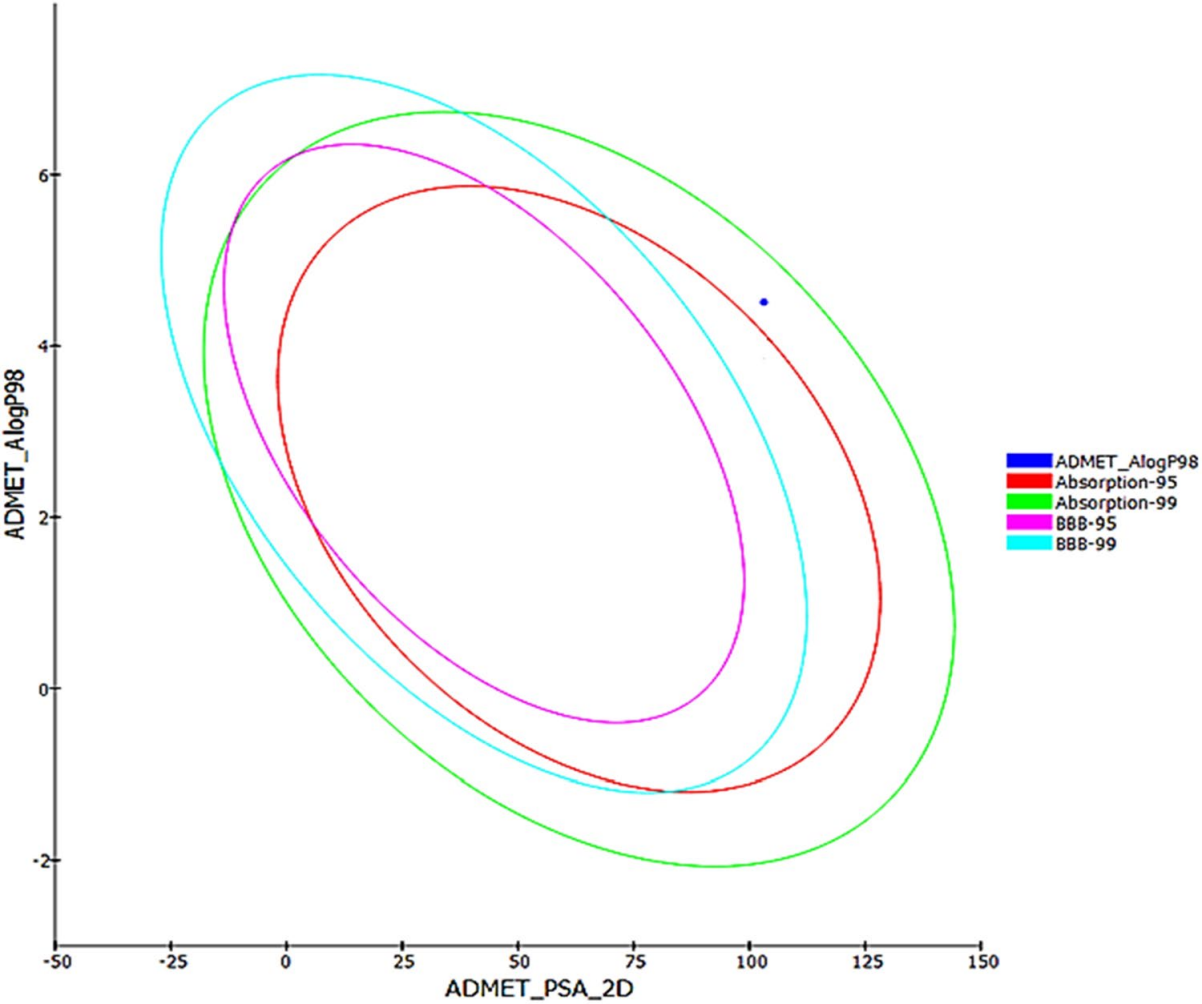
ADME plot for compounds **10i**.

Absorption, distribution, metabolism, and excretion (ADME) properties of **10i** was predict using Biovia Discovery Studio. **10i** showed a promising pharmacokinetic profile with good to moderate GIT absorption; no blood brain permeability indicating the absence of potential CNS side effects and no inhibition for Cytochrome P450 2D6 indicating no possible drug interactions. Results are illustrated in Table 7 and Fig. 12.

**Table 7:** Pharmacokinetic prediction results of compounds **10i** using Biovia Discovery Studio.

Parameter	**10i**
GIT absorption	1
Blood Brain permeability	4
Cytochrome P450 2D6	False
Absorption: 0 (Good), 1 (Moderate), 2 (Poor), 3 (Very poor)	
BBB permeability: 0 (Very high), 1 (High), 2 (Medium), 3 (Low), 4 (Undefined)

Additionally, drug-likeness properties for this compound were predicted using SWISS ADME (http://www.swissadme.ch/index.php) and the predicted parameters are illustrated in Table 8. **10i** was predicted to follow Lipinski’s rule of five with zero violations indicating good oral bioavailability, also it was predicted not to be a P-glycoprotein substrate with low potential of being effluxed from cancer cells. Undesirable substructures alert such as Pains and Brenk alerts were not predicted for this compound indicating that it is specific.

**Table 8:** Predicted pharmacokinetic parameters for compounds **10i** using SWISS ADME.

Parameter	**10i**
**Lipinski**	Yes violation
**P-gp substrate**	No
**PAINS alert**	No alert
**Brenk alert**	No alert

## Conclusion

In the current study, a number of novel quinolone-3-carboxamide based molecules **10a-p** were designed and synthesized. All the targeted molecules were tested in vitro for their VEGFR-2 inhibitory activity and their cytotoxic activity against HepG2 tumor cells. Generally, most of the compounds gave potent to moderate VEGFR-2 inhibitory activity with IC_50_ ranging from 36 nM to 0.578 µM compared to the reference drug sorafenib as a reference drug with IC_50_ = 45 nM, while seven compounds showed better cytotoxic activity than sorafenib. Compounds **10i** and **10o** exhibited the best VEGFR-2 inhibitory activity with potent cytotoxic activity against HepG2 tumor cell line, therefore their selectivity index was calculated showing their tolerable cytotoxicity profile. As the most potent VEGFR-2 inhibitor with high selective cytotoxicity, derivative **10i** was taken forward for further mechanistic investigations as a representative of this series of new quinolones. HepG2 cell apoptosis profiling, cell cycle analysis, Caspase-7 and BAX protein expression levels and VEGFR-2 gene expression level upon treatment of HepG2 cells with **10i** were assessed and found to be significantly affected by treatment favoring apoptosis of these cancer cells. Furthermore, in silico docking study was performed to predict the possible interactions between these molecules and the amino acids within the binding site of VEGFR-2; the results were fundamentally in agreement with the biological data. Moreover, in silico ADME prediction of **10i**’s pharmacokinetic and drug-likeness properties showed promising results. As a conclusion, compound **10i** could serve as a suitable candidate for further investigation as an antiproliferative agent acting through VEGFR-2 inhibition.

## Experimental

### Chemistry

Melting points were determined using Stuart SMP3 version 5.0 apparatus, using open capillary method and were uncorrected. Infrared spectra were recorded on Shimadzu-FTIR spectroscopy using KBR discs and obtained in wave number (cm^−1^), Faculty of Pharmacy, Cairo University. ^1^H NMR spectra were carried out using Bruker spectrophotometer operating (400 MHz) at Faculty of Pharmacy, Ain Shams University or using JEOL spectrophotometer (500 MHz) at the National Research Center, in DMSO-*d*_6_ as solvent and the chemical shifts were given in *δ* as parts per million (ppm) downfield from tetramethylsilane (TMS) as internal standard. ^13^C NMR spectra were obtained using Bruker spectrophotometer operating (100 MHz) at Faculty of Pharmacy, Ain Shams university or using JEOL spectrophotometer (125 MHz) at the National Research Center, in DMSO-*d*_6_ as solvent and the chemical shifts were given in δ as parts per million (ppm) downfield from tetramethylsilane (TMS) as internal standard. Elemental microanalysis was performed at the Regional center for Mycology and Biotechnology, Al-Azhar University. The reactions were monitored by TLC (Merck, Germany), methylene chloride/methanol (9:1) mixture was used as eluting solvent and spots were visualized by ultraviolet lamp. All reagents and solvents were purified and dried using the standard techniques. All compounds were chemically named using chemical name facility of ChemDraw Ultra 12.0 software.

#### Synthesis of diethyl 2-(((4-substituted phenyl) amino) methylene) malonate (**2a,b**)

The substituted aniline **1a** or **1b** (0.02 mol) was heated with diethyl ethoxymethylenemalonate (0.0216 mol, 4.67 gm, 4.36 mL) at 125–135 °C for 6 h causing ethanol to be liberated. The corresponding diethyl anilinomethylenemalonate derivatives **2a,b** were produced as a brown oil which was cooled down into a semi-solid. The obtained product was purified using hexane, followed by petroleum ether, and finally left to dry^23,46–48^.

##### Diethyl 2-(((4-chlorophenyl)amino)methylene)malonate (**2a**)^46^

Yield: 78.5%; M.p.: 63–65 °C as reported.

##### Diethyl 2-(((4-fluorophenyl)amino)methylene)malonate (**2b**)^23^

Yield: 55.7%; M.p.: 68–70 °C as reported.

#### Synthesis of 6-substituted-ethyl 4-oxo-1,4-dihydroquinoline-3-carboxylate (**3a,b**)

The diethyl anilinomethylenemalonate derivative **2a** or **2b** (0.015 mol) was heated with diphenyl ether (0.01 mol, 1.7 gm, 1.5 mL) at 220 °C for 1 h then moved to a sand bath for 2 h. Cooling of this reaction mixture to provide a precipitate to be filtered off, washed with hexane then diethyl ether and dried to afford the 6-substituted ethyl 4-oxo-1,4-dihydroquinoline-3-carboxylate **3a,b**^23,49^.

##### Ethyl 6-chloro-4-oxo-1,4-dihydroquinoline-3-carboxylate (**3a**)^49^

Yield: 62.7%; M.p.: > 300 °C as reported.

##### Ethyl 6-fluoro-4-oxo-1,4-dihydroquinoline-3-carboxylate (**3b**)^23^

Yield: 44%; M.p.: 273–275 °C as reported.

#### Synthesis of 4-oxo-1,4-dihydroquinoline-3-carboxylic acids(**4a,b**)

A suspension of the 6-substituted quinolone ester **3a** or **3b** (0.01 mmol) in 1 M aqueous NaOH (60 mL) was refluxed for 3 h. After cooling to room temperature, this mixture was filtered using Buchner funnel. Filtrate was then acidified using diluted HCl. The formed precipitate was filtered and washed several times with water then left to dry yielding the pure acid^49,50^.

##### 6-Chloro-4-oxo-1,4-dihydroquinoline-3-carboxylic acid (**4a**)^51^

Yield: 67%; M.p.: 260–262 °C as reported. ^1^H NMR (400 MHz, DMSO-*d*_6_) *δ* ppm: 15.06 (s, 1H, COOH, D_2_O exchangeable), 8.91 (s, 1H, H-2-quinoline), 8.18 (d, *J* = 2.4 Hz, 1H, H-5-quinoline), 7.90 (dd, *J* = 8.8, 2.4 Hz, 1H, H-7-quinoline), 7.83 (d, *J* = 8.9 Hz, 1H, H-8-quinoline).

##### 6-Fluoro-4-oxo-1,4-dihydroquinoline-3-carboxylic acid (**4b**)^23^

Yield: 77.2%; M.p.: 286–288 °C as reported. ^1^H NMR (500 MHz, DMSO-*d*_6_) *δ* ppm: 15.13 (s, 1H, COOH, D_2_O exchangeable), 8.88 (s, 1H, H-5-quinoline), 7.85 range (m, 2H, H-5 and H-8-quinoline), 7.76 (dt, *J* = 6.7, 1.95 Hz, 1H, H-7-quinoline).

#### Synthesis of 4-nitrobenzoylazide (**6**)^27^

Sodium azide (2.86 g, 0.0441 mol) was dissolved in 15 mL water and cooled in an ice bath, A solution of 4-nitrobenzoyl chloride (**5**) (5 gm, 0.027 mol) dissolved in 15 mL dry acetone was added dropwise over 1 h. The reaction mixture was stirred for 30 min followed by the addition of 15 mL water and continuous stirring for another 30 min. A precipitate will be formed and collected by filtration followed by washing with water and finally dried dry to yield 4-nitrobenzoyl azide (**6**) as slightly yellow flakes. Yield: 60%; Melting point: 71 °C as reported.

#### Synthesis of 4-nitrobenzoylisocyanate (**7**)^52^

Reflux 4-nitrobenzoyl azide (**6**) (3.84 gm, 0.02 mmol) in 60 mL dry toluene for 1 h to afford the 4-nitrobenzoyl isocyanate (**7**), which was employed in the next reaction as an intermediate without further isolation and purification^52,53^.

#### Synthesis of 1-(4-Nitrophenyl)-3-arylurea derivatives (**8a-i**)

To a hot solution of 0.02 mmol of 1-isocyanato-4-nitrobenzene (**7**) in 60 mL dry toluene, 0.02 mmol of substituted aniline was added and refluxed for 6 h. A precipitate was formed and filtered on hot then washed with hot toluene to yield 1-(4-nitrophenyl)-3-phenylurea derivatives **8a-i.**

##### 1-(4-Nitrophenyl)-3-phenylurea (**8a**)^53^

Yield: 99%; M.p.: 207–209 °C as reported.

##### 1-(4-Nitrophenyl)-3-(***p***-tolyl)urea (**8b**)^54^

Yield: 85%; M.p.: 187–189 °C as reported.

##### 1-(4-Nitrophenyl)-3-(***m***-tolyl)urea (**8c**)^55^

Yield: 92%; M.p.: 199-201^0^C as reported.

##### 1-(4-Nitrophenyl)-3-(***o***-tolyl)urea (**8d**)^55^

Yield: 91%; M.p.: 195–197 °C.

##### 1-(4-Fluorophenyl)-3-(4-nitrophenyl)urea (**8e**)^56^

Yield: 89%; M.p.: 249–251 °C as reported.

##### 1-(4-Nitrophenyl)-3-(3-(trifluoromethyl)phenyl)urea (**8f**)^57^

Yield: 94%; M.p.: 259–261 °C as reported.

##### 1-(4-Cyanophenyl)-3-(4-nitrophenyl)urea (**8g**)^56^

Yield: 90%; M.p.: 289–291 °C as reported.

##### 1-(Benzo[d]thiazol-6-yl)-3-(4-nitrophenyl)urea (**8h**)^58^

Yield: 92%; M.p.: 237–239 °C as reported.

##### 1-(4-Isopropylphenyl)-3-(4-nitrophenyl)urea (**8i**)^59^

Yield: 91%; M.p.: 218–220 °C.

#### Synthesis of 1-(4-Aminophenyl)-3-arylurea derivatives (**9a-i**)

18 gm (0.075 mol) of crystallized sodium sulfide, Na_2_S.9H_2_O, were dissolved in 50 ml of water; then add 6 gm (0.0714 mol) of finally powdered sodium hydrogen carbonate in small portions with continuous stirring. After dissolving all of the carbonate, add 50 mL of methanol, the precipitated sodium carbonate was filtered off by vacuum filtration. Three 8 mL portions of methanol were used to wash the precipitate. The filtrate and washings were retained which contain about 3.9 gm of sodium hydrogen sulfide (NaHS) in solution and was used directly afterwards for reduction. Dissolving 0.015 mol of 1-(4-nitrophenyl)-3-arylurea derivatives **8a-i** in 50 mL methanol on hot followed by the addition with shaking of half of the methanolic solution of sodium hydrogen sulfide previously prepared was performed, reflux for 16 h. was done while ignoring any further sodium carbonate that might precipitate. The reaction mixture was left to cool then filtered to collect the precipitate. For purification of the product; the precipitate was dissolved in dil. HCl, filtered using Buchner funnel, filtrate was neutralized using NaHCO_3_ resulting in reprecipitation of the pure 1-(4-aminophenyl)-3-arylurea derivatives **9a-i** which were collected by filtration and dried.

##### 1-(4-Aminophenyl)-3-phenylurea (**9a**)^57^

Yield: 21%; M.p.: 224–227 °C; ^1^H NMR (400 MHz, DMSO-*d*_*6*_) *δ* ppm: 8.47 (s, 1H, NH, D_2_O exchangeable), 8.13 (s, 1H, NH, D_2_O exchangeable), 7.41 (d, *J* = 7.7 Hz, 2H, Ar–H), 7.22 (t, *J* = 7.8 Hz, 2H, Ar–H), 7.07 (d, *J* = 8.6 Hz, 2H, Ar–H), 6.90 (t, *J* = 7.3 Hz, 1H, Ar–H), 6.51 (d, *J* = 8.7 Hz, 2H, Ar–H), 4.76 (s, 2H, NH_2_, D_2_O exchangeable).

##### 1-(4-Aminophenyl)-3-(***p***-tolyl)urea (**9b**)^60^

Yield: 28%; M.p.: > 300 °C; ^1^H NMR (400 MHz, DMSO-*d*_*6*_) *δ* ppm: 8.36 (s, 1H, NH, D_2_O exchangeable), 8.08 (s, 1H, NH, D_2_O exchangeable), 7.29 (d, *J* = 8.0 Hz, 2H, Ar–H), 7.04 (m, 4H, Ar–H), 6.49 (d, *J* = 8.3 Hz, 2H, Ar–H), 4.74 (s, 2H, NH_2_, D_2_O exchangeable), 2.22 (s, 3H, -CH_3_).

##### 1-(4-Aminophenyl)-3-(***m***-tolyl)urea (**9c**)^61^

Yield: 36.3%; M.p.: > 300 °C; ^1^H NMR (500 MHz, DMSO-*d*_*6*_) *δ* 8.39 (s, 1H, NH, D_2_O exchangeable), 8.11 (s, 1H, NH, D_2_O exchangeable), 7.26 (s, 1H, Ar–H), 7.18 (d, *J* = 8.8 Hz, 1H, Ar–H), 7.10 (t, *J* = 7.7 Hz, 1H, Ar–H), 7.05 (d, *J* = 8.7 Hz, 2H, Ar–H), 6.73 (d, *J* = 7.4 Hz, 1H, Ar–H), 6.49 (d, *J* = 8.7 Hz, 2H, Ar–H), 4.79 (s, 2H, NH_2_, D_2_O exchangeable), 2.25 (s, 3H, -CH_3_).

##### 1-(4-Aminophenyl)-3-(***o***-tolyl)urea (**9d**)^60^

Yield:30.55%; M.p.: > 300 °C; ^1^H NMR (500 MHz, DMSO-*d*_*6*_) *δ* ppm: 8.54 (s, 1H, NH, D_2_O exchangeable), 7.84 (d, *J* = 8.4 Hz, 1H, Ar–H), 7.72 (s, 1H, NH, D_2_O exchangeable), 7.10 (m, 7.6 Hz, 2H, Ar–H), 7.07 (d, *J* = 8.8 Hz, 2H, Ar–H), 6.87 (m, 1H, Ar–H), 6.50 (d, *J* = 8.8 Hz, 2H, Ar–H), 4.77 (s, 2H, NH_2_, D_2_O exchangeable), 2.21 (s, 3H, -CH_3_).

##### 1-(4-Aminophenyl)-3-(4-fluorophenyl)urea (**9e**)^62^

Yield: 30%; M.p.: 273–275 °C; ^1^H NMR (400 MHz, DMSO-*d*_*6*_) *δ* ppm: 8.70 (s, 1H, NH, D_2_O exchangeable), 8.28 (s, 1H, NH, D_2_O exchangeable),7.45–7.41 (m, 2H, Ar–H), 7.10–7.05 (m, 4H, Ar–H), 6.52–6.50 (m, 2H, Ar–H), 4.74 (s, 2H, NH_2_, D_2_O exchangeable).

##### 1-(4-Aminophenyl)-3-(3-(trifluoromethyl)phenyl)urea (**9f**)^57^

Yield: 6.1%; M.p.: 147–149 °C; ^1^H NMR (500 MHz, DMSO-*d*_*6*_) *δ* ppm: 9.34 (s, 1H, NH, D_2_O exchangeable), 8.75 (s, 1H, NH, D_2_O exchangeable), 8.04 (s, 1H, Ar–H), 7.58 (d, *J* = 8.3 Hz, 1H, Ar–H), 7.41 (t, *J* = 7.9 Hz, 1H, Ar–H), 7.21 (d, *J* = 7.8 Hz, 1H, Ar–H), 7.11 (d, *J* = 8.3 Hz, 2H, Ar–H), 6.48 (d, *J* = 8.2 Hz, 2H, Ar–H), 4.77 (s, 2H, NH_2_, D_2_O exchangeable).

##### 1-(4-Aminophenyl)-3-(4-cyanophenyl)urea (**9g**)^62^

Yield: 21%; M.p.: 217–219 °C; ^1^H NMR (500 MHz, DMSO-*d*_*6*_) *δ* ppm: 9.3 (s, 1H, NH, D_2_O exchangeable), 8.79 (s, 1H, NH, D_2_O exchangeable),7.74 (d, *J* = 8.9 Hz, 2H, Ar–H), 7.53 (d, *J* = 8.8 Hz, 2H, Ar–H), 7.13 (d, *J* = 8.3 Hz, 2H, Ar–H), 6.47 (d, *J* = 8.9 Hz, 2H, Ar–H), 4.75 (s, 2H, NH_2_, D_2_O exchangeable).

##### 1-(4-Aminophenyl)-3-(benzo[d]thiazol-6-yl)urea (**9h**)

Yield: 33%; M.p.: > 300 °C; ^1^H NMR (500 MHz, DMSO-*d*_*6*_) *δ* ppm: 9.19 (s, 1H, NH, D_2_O exchangeable), 8.89 (s, 1H, NH, D_2_O exchangeable), 8.35 (m, 2H, H-2 and H-7 benzo[d]thiazole), 7.95 (d, *J* = 8.8 Hz, 1H, H-4 benzo[d]thiazole), 7.45 (d, *J* = 8.9 Hz, 1H, H-5 benzo[d]thiazole), 7.10 (d, *J* = 8.3 Hz, 2H, Ar–H), 6.52 (d, *J* = 8.2 Hz, 2H, Ar–H), 4.81 (s, 1H, -NH, D_2_O exchangeable), 4.78 (s, 1H, NH, D_2_O exchangeable).

##### 1-(4-Aminophenyl)-3-(4-isopropylphenyl)urea (**9i**)

Yield: 5%; M.p.: 183 °C; ^1^H NMR (500 MHz, DMSO-*d*_*6*_) *δ* ppm: 8.43 (s, 1H, NH, D_2_O exchangeable), 8.14 (s, 1H, NH, D_2_O exchangeable), 7.31 (d, *J* = 8.1 Hz, 2H, Ar–H), 7.13–7.06 (m, 4H, Ar–H), 6.49 (d, *J* = 8.3 Hz, 2H, Ar–H) C_6_H_4_), 4.76 (s, 1H, NH, D_2_O exchangeable), 4.73 (s, 1H, NH, D_2_O exchangeable) 2.84–2.77 (m, 1H, -CH(CH_3_)_2_), 1.16 (d, *J* = 7.0 Hz, 6H, -CH(CH_3_)_2_).

#### Synthesis of 4-oxo-N-(4-(3-phenylureido)phenyl)-1,4-dihydroquinoline-3-carboxamide derivatives (**10a-p**)

The quinoline carboxylic acid **4a** or **4b** (1.0 mmol) is stirred with HATU (1.5 mmol) and diisopropylethylamine (3 mmol) in anhydrous dimethyl formamide for 1 h in an ice bath (0 °C). This was followed by addition of the corresponding amine **9a-i** (1.5 mmol). The mixture was left overnight at room temperature with continuous stirring. The mixture was poured slowly over iced water to form a precipitate which was filtered and subjected to sequential washes with ethyl acetate, ethanol and finally methanol to yield the final targeted compounds **10a-p**. (IR, ^1^H NMR and ^13^C NMR charts for spectral analyses for derivatives **10a-p** are provided in the supplementary material, Figures S1-S45).

##### 6-Chloro-4-oxo-*N*-(4-(3-phenylureido)phenyl)-1,4-dihydroquinoline-3-carboxamide (**10a**)

Pale yellowish orange fine precipitate; Yield: 62.5%; M.p.: > 300 °C. IR (KBr, cm^−1^): 3398–3263 (4 NH), 3086 (CH aromatic), 1674–1650 (3 C=O).^1^H NMR (400 MHz, DMSO-*d*_*6*_) *δ* ppm: 12.22 (s, 1H, CONH, D_2_O exchangeable), 8.90 (s, 1H, H-2-quinoline), 8.64 (s, 2H,2NH, D_2_O exchangeable), 8.25 (s, 1H, H-5 quinoline), 7.83 (d, *J* = 9.1 Hz, 1H, H-7 quinoline), 7.79 (d, *J* = 9.0 Hz, 1H, H-8 quinoline), 7.64 (d, *J* = 9.1, 2H, Ar–H), 7.46 – 7.44 (m, 4H, Ar–H), 7.30 – 7.26 (m, 2H, Ar–H), 6.94 (t, *J* = 9.2 Hz, 1H, Ar–H). ^13^C NMR (101 MHz, DMSO-*d*_*6*_) *δ* ppm:175.04, 162.17, 152.57, 144.59, 139.79, 138.12, 135.41, 133.10, 132.89, 129.79, 128.76 (2C), 127.05, 124.37, 121.84, 121.72, 120.20 (2C), 118.86 (2C), 118.15(2C), 111.01. Anal. Calcd for C_23_H_17_ClN_4_O_3_ (432.86): C, 63.82; H, 3.96; N, 12.94.; Found: C, 64.05; H, 4.12; N, 13.18.

##### 6-Chloro-4-oxo-*N*-(4-(3-(*p*-tolyl)ureido)phenyl)-1,4-dihydroquinoline-3-carboxamide (**10b**)

Yellow precipitate; Yield: 32.8%; M.p.: > 300 °C. IR (KBr, cm^−1^): 3275 (br., 4 NH), 3062 (CH aromatic), 2962 (CH aliphatic), 1654–1639 (3 C=O). ^1^H NMR (400 MHz, DMSO-*d*_*6*_) *δ* ppm: 12.19 (s, 1H, CONH, D_2_O exchangeable), 8.89 (s, 1H, H-2-quinoline), 8.57 (s, 1H, NH, D_2_O exchangeable), 8.51 (s, 1H, NH, D_2_O exchangeable), 8.23 (d, *J* = 2.3 Hz, 1H, H-5 quinoline),7.81(dd, *J* = 8.6, 2.3 Hz, 1H, H-7 quinoline), 7.76 (d, *J* = 8.9 Hz, 1H, H-8 quinoline),7.63 (d, *J* = 8.5 Hz, 2H, Ar–H),7.43 (d, *J* = 8.4 Hz, 2H, Ar–H), 7.33 (d, *J* = 8.0 Hz, 2H, Ar–H),7.07 (d, *J* = 8.0 Hz, 2H, Ar–H),2.24 (s, 3H, CH_3_). ^13^C NMR (101 MHz, DMSO-*d*_*6*_) *δ* ppm:175.07, 162.10, 152.61, 144.47, 137.96, 137.20, 135.53, 132.98, 130.53, 129.83 (2C), 129.15 (2C), 127.03, 124.38, 121.73, 120.19 (2C), 118.78 (2C), 118.25 (2C), 111.05, 20.32. Anal. Calcd for C_24_H_19_ClN_4_O_3_ (446.89): C, 64.50; H, 4.29; N, 12.54. Found: C, 64.29; H, 4.53; N, 12.79.

##### 6-Chloro-4-oxo-*N*-(4-(3-(*m*-tolyl)ureido)phenyl)-1,4-dihydroquinoline-3-carboxamide (**10c**)

Buff precipitate; Yield: 23.88%; M.p.: > 300 °C. IR (KBr, cm^−1^): 3348 (br., 4 NH), 3082 (CH aromatic), 3966 (CH aliphatic), 1670–1651 (3 C=O). ^1^H NMR (400 MHz, DMSO-*d*_*6*_) *δ* ppm: 12.20 (s, 1H, CONH, D_2_O exchangeable), 8.88 (s, 1H, H-2-quinoline), 8.61 (s, 1H, NH, D_2_O exchangeable), 8.55 (s, 1H, NH, D_2_O exchangeable), 8.23 (d, *J* = 2.4 Hz, 1H, H-5 quinoline), 7.80 (dd, *J* = 8.7, 2.5 Hz, 1H, H-7 quinoline),7.76 (d, *J* = 9.0 Hz, 1H, H-8 quinoline),7.63 (d, *J* = 8.6 Hz, 2H, Ar–H), 7.44 (d, *J* = 8.8 Hz, 2H, Ar–H), 7.30 (s, 1H, Ar–H),7.22 (d, *J* = 8.2 Hz, 1H, Ar–H), 7.13 (t, *J* = 7.8 Hz, 1H, Ar–H), 6.77 (d, *J* = 7.4 Hz, 1H, Ar–H), 2.28 (s, 3H, CH_3_). ^13^C NMR (101 MHz, DMSO-* d*_*6*_) *δ* ppm: 175.07, 162.11, 152.54, 144.45, 139.69, 137.96, 137.91, 135.44, 133.05, 132.92, 129.82, 128.59, 127.02, 124.38, 122.47, 121.72, 120.19 (2C), 118.82 (2C), 118.67, 115.33, 111.04, 21.23. Anal. Calcd for C_24_H_19_ClN_4_O_3_ (446.89): C, 64.50; H, 4.29; N, 12.54. Found: C, 64.34; H, 4.50; N, 12.76.

##### 6-Chloro-4-oxo-*N*-(4-(3-(*o*-tolyl)ureido)phenyl)-1,4-dihydroquinoline-3-carboxamide (**10d**)

Yellow precipitate; Yield: 37.3%_;_ M.p.: > 300 °C. IR (KBr, cm^−1^): 3282 (br., 4 NH), 3082 (CH aromatic), 2962 (CH aliphatic), 1660–1639 (3 C=O). ^1^H NMR (500 MHz, DMSO-*d*_*6*_) *δ* ppm: 12.23 (s, 1H, CONH, D_2_O exchangeable), 9.02 (s, 1H, NH, D_2_O exchangeable), 8.89 (s, 1H, H-2-quinoline), 8.23 (s, 1H, H-5 quinoline), 7.91 (s, 1H, NH, D_2_O exchangeable), 7.86 – 7.82 (m, 2H. H-7 and H-8-quinoline), 7.78 (m, 1H, Ar–H), 7.64 (d, *J* = 8.9 Hz, 2H, Ar–H), 7.45 (d, *J* = 8.6 Hz, 2H, Ar–H), 7.17–7.12 (m, 2H, Ar–H), 6.95–6.92 (m, 1H, Ar–H), 2.24 (s, 3H, CH_3_). ^13^C NMR (126 MHz, DMSO-*d*_*6*_) *δ* ppm: 175.05, 162.13, 152.67, 144.50, 138.04, 137.46, 135.59, 132.97, 132.96, 130.14, 129.79, 127.45, 127.03, 126.11, 124.36, 122.57, 121.78, 121.01, 120.24 (2C), 118.65 (2C), 111.04, 17.83. Anal. Calcd for C_24_H_19_ClN_4_O_3_ (446.89):C, 64.50; H, 4.29; N, 12.54. Found: C, 64.59; H, 4.38; N, 12.68.

##### 6-Chloro-*N*-(4-(3-(4-fluorophenyl)ureido)phenyl)-4-oxo-1,4-dihydroquinoline-3-carboxamide (**10e**)

Light yellow precipitate; Yield: 29.6%; M.p.: > 300 °C. IR (KBr, cm^−1^): 3290 (br., 4 NH), 3082–3008 (CH aromatic), 1659–1636 (3 C=O). ^1^H NMR (500 MHz, DMSO-*d*_*6*_) *δ* ppm: 12.26 (s, 1H, CONH, D_2_O exchangeable), 8.90 (s, 1H, H-2-quinoline), 8.70 (s, 1H, NH, D_2_O exchangeable), 8.65 (s, 1H, NH, D_2_O exchangeable), 8.23 (d, *J* = 2.4 Hz, 1H, H-5-quinoline), 7.81 (dd, *J* = 8.7, 2.5 Hz, 1H, H-7-quinoline), 7.77 (d, *J* = 9.0 Hz, 1H, H-8-quinoline), 7.64 (d, *J* = 8.6 Hz, 2H, Ar–H), 7.48–7.43 (m, 4H, Ar–H), 7.13–7.09 (m, 2H, Ar–H). ^13^C NMR (101 MHz, DMSO-*d*_*6*_) *δ* ppm: 175.07, 162.25, 152.71, 144.67, 138.22, 136.14, 135.39, 133.17, 129.83, 127.10, 124.40, 121.93, 120.23 (2C), 120.01, 119.93 (2C), 118.99 (2C), 115.39 (2C), 115.17, 111.03. Anal. Calcd for C_23_H_16_ClFN_4_O_3_ (450.85): C, 61.27; H, 3.58; N, 12.43. Found: C, 61.43; H, 3.7; N, 12.71.

##### 6-Chloro-4-oxo-*N*-(4-(3-(3-(trifluoromethyl)phenyl)ureido)phenyl)-1,4-dihydroquinoline-3-carboxamide (**10f**)

Yellow precipitate; Yield: 61%; M.p.: > 300 °C. IR (KBr, cm^−1^): 3429–3259 (4 NH), 3093 (CH aromatic), 1670 (br., 3 C=O). ^1^H NMR (500 MHz, DMSO-*d*_*6*_) *δ* ppm: 12.23 (s, 1H, CONH, D_2_O exchangeable), 9.03 (s, 1H, NH, D_2_O exchangeable), 8.90 (s, 1H, H-2-quinoline), 8.78 (s, 1H, NH, D_2_O exchangeable), 8.24 (s, 1H, H-5-quinoline), 8.02 (s, 1H, Ar–H), 7.84 (d, *J* = 9.0 Hz, 1H, H-7-quinoline), 7.78 (d, *J* = 8.8 Hz, 1H, H-8-quinoline), 7.65 (d, *J* = 8.4 Hz, 2H, Ar–H), 7.56 (d, *J* = 8.3 Hz, 1H, Ar–H), 7.49 (m, 1H, Ar–H), 7.45 (d, *J* = 8.4 Hz, 2H, Ar–H), 7.30 (d, *J* = 7.8 Hz, 1H, Ar–H). ^13^C NMR (126 MHz, DMSO-*d*_*6*_) *δ* ppm: 175.17, 162.22, 155.76, 152.60, 152.19, 147.99, 144.52, 140.72, 137.98, 135.07, 133.44, 133.05, 129.93, 127.10, 124.45, 121.83, 121.82, 120.25 (2C), 119.32 (2C), 118.04, 114.16, 111.12. Anal. Calcd for C_24_H_16_ClF_3_N_4_O_3_ (500.86): C, 57.55; H, 3.22; N, 11.19. Found: C, 57.81; H, 3.49; N, 11.42.

##### 6-Chloro-*N*-(4-(3-(4-cyanophenyl)ureido)phenyl)-4-oxo-1,4-dihydroquinoline-3-carboxamide (**10g**)

Brown precipitate; Yield: 48.5%; M.p.: > 300 °C. IR (KBr, cm^−1^): 3356–3271 (4 NH), 3078 (CH aromatic), 2225 (CN), 1658–1640 (3 C=O). ^1^H NMR (500 MHz, DMSO-*d*_*6*_) *δ* ppm: 12.25 (s, 1H, CONH, D_2_O exchangeable), 8.93 (s, 1H, NH, D_2_O exchangeable), 8.90 (s, 1H, NH, D_2_O exchangeable), 8.76 (s, 1H, H-2-quinoline), 8.24 (s, 1H, H-5-quinoline), 7.85–7.77 (m, 4H, Ar–H), 7.67 – 7.64 (m, 2H, H-7 and H-8-quinoline), 7.51 (d, *J* = 6.8 Hz, 2H, Ar–H), 7.46 (d, *J* = 6.8 Hz, 2H, Ar–H). ^13^C NMR (126 MHz, DMSO-*d*_*6*_) *δ* ppm: 175.05, 167.54, 162.24, 152.35, 144.65, 142.59, 138.19, 135.11, 133.24, 132.89, 129.79 (2C), 128.50, 127.07, 124.37, 121.91, 120.21 (2C), 119.09 (2C), 118.01, 117.04 (2C), 111.00. Anal. Calcd for C_24_H_16_ClN_5_O_3_ (457.87): C, 62.96; H, 3.52; N, 15.30. Found: C, 62.83; H, 3.71; N, 15.48.

##### *N*-(4-(3-(Benzo[d]thiazol-6-yl)ureido)phenyl)-6-chloro-4-oxo-1,4-dihydroquinoline-3-carboxamide (**10h**)

Buff precipitate; Yield: 81%_;_ M.p.: > 300 °C. IR (KBr, cm^−1^): 3417–3282 (4 NH), 3074 (CH aromatic), 1708–1654 (3 C=O). ^1^H NMR (500 MHz, DMSO-*d*_*6*_) *δ* ppm: 12.47 (s, 1H, CONH, D_2_O exchangeable), 9.20 (s, 1H, H-2-quinoline), 8.95 (s, 1H, NH, D_2_O exchangeable), 8.90 (s, 1H, NH, D_2_O exchangeable), 8.74 (s, 1H, H-2 benzo[d]thiazole), 8.37 (d, *J* = 2.0 Hz, 1H, H-5-quinoline), 8.23 (d, *J* = 2.3 Hz, 1H, H-7-quinoline), 7.97 (d, *J* = 8.8 Hz, 1H, H-8-quinoline), 7.78 – 7.65 (m, 4H, Ar–H), 7.49–7.45 (m, 3H, H-4, H-5, H-7-benzo[d]thiazole).^13^C NMR (126 MHz, DMSO-*d*_*6*_) *δ* ppm: 174.70, 162.82, 153.68, 152.67, 148.29, 145.93, 137.71, 135.06, 134.50, 133.54, 132.34, 129.33, 127.36, 124.27, 123.13, 122.96, 120.16 (2C), 119.21 (2C), 119.08, 118.16, 110.71, 110.28. Anal. Calcd for C_24_H_16_ClN_5_O_3_S (489.93): C, 58.84; H, 3.29; N, 14.29; S, 6.54. Found: C, 59.12; H, 3.46; N, 14.58; S, 6.63.

##### 6-Chloro-*N*-(4-(3-(4-isopropylphenyl)ureido)phenyl)-4-oxo-1,4-dihydroquinoline-3-carboxamide (**10i**)

Brown precipitate; Yield: 48%; M.p.: > 300 °C. IR (KBr, cm^−1^): 3317 (br., 4 NH), 3043–3001 (CH aromatic), 3958 (CH aliphatic), 1651 (br., 3 C=O). ^1^H NMR (500 MHz, DMSO-*d*_*6*_) *δ* ppm: 12.22 (s, 1H, CONH, D_2_O exchangeable), 8.90 (s, 1H, H-2-quinoline), 8.60 (s, 1H, NH, D_2_O exchangeable), 8.55 (s, 1H, NH, D_2_O exchangeable), 8.24 (d, *J* = 2.5 Hz, 1H, H-5-quinoline), 7.84 (dd, *J* = 8.8, 2.5 Hz, 1H, H-7-quinoline), 7.78 (d, *J* = 8.8 Hz, 1H, H-8-quinoline), 7.63 (d, *J* = 8.6 Hz, 2H, Ar–H), 7.42 (d, *J* = 8.7 Hz, 2H, Ar–H), 7.35 (d, *J* = 8.3 Hz, 2H, Ar–H), 7.13 (d, *J* = 8.3 Hz, 2H, Ar–H), 2.85–2.79 (m, 1H, -CH(CH_3_)_2_), 1.17 (d, *J* = 6.9 Hz, 6H, -CH(CH_3_)_2_). ^13^C NMR (126 MHz, DMSO-*d*_*6*_) *δ* ppm: 175.14, 162.20, 152.69, 144.59, 141.89, 138.10, 137.49, 135.59, 133.00, 129.89, 127.87, 127.12, 126.51(2C), 124.44, 121.86, 120.27 (2C), 118.87 (2C), 118.44 (2C), 111.12, 32.80, 24.05 (2C). Anal. Calcd for C_26_H_23_ClN_4_O_3_ (474.94): C, 65.75; H, 4.88; N, 11.80. Found: C, 65.92; H, 4.95; N, 11.97.

##### 6-Fluoro-4-oxo-*N*-(4-(3-phenylureido)phenyl)-1,4-dihydroquinoline-3-carboxamide (**10j**)

Bright yellow fine precipitate; Yield: 36.8%; M.p.: > 300 °C. IR (KBr, cm^−1^): 3271 (br., 4 NH), 3097 (CH aromatic),1685–1651 (3 C=O). ^1^H NMR (400 MHz, DMSO-*d*_*6*_) *δ* ppm: 12.25 (s, 1H, CONH, D_2_O exchangeable), 8.86 (s, 1H, H-2-quinoline), 8.62 (s, 2H, NH, D_2_O exchangeable), 7.93 (d, *J* = 9.0 Hz, 1H, H-5-quinoline), 7.80–7.69 (m, 2H, H-7 and H-8-quinoline), 7.63 (d, *J* = 8.5 Hz, 2H, Ar–H), 7.46–7.44 (m, 4H, Ar–H), 7.25 (t, *J* = 7.5 Hz, 2H, Ar–H), 6.94 (t, *J* = 7.3 Hz, 1H, Ar–H).^13^C NMR (101 MHz, DMSO-*d*_*6*_) *δ* ppm: 175.48, 162.36, 158.18, 152.67, 144.05, 139.82, 136.03, 135.45, 133.20, 128.85 (2C), 127.40, 122.14, 121.84, 120.27 (2C), 118.98 (2C), 118.27 (2C), 110.27, 109.87, 109.64. Anal. Calcd for C_23_H_17_FN_4_O_3_ (416.40): C, 66.34; H, 4.11; N, 13.45. Found: C, 66.23; H, 4.37; N, 13.69.

##### 6-Fluoro-4-oxo-*N*-(4-(3-(*p*-tolyl)ureido)phenyl)-1,4-dihydroquinoline-3-carboxamide (**10k**)

Yellow precipitate; Yield: 46.8%; M.p.: > 300 °C. IR (KBr, cm^−1^): 3360–3221 (4 NH), 3070 (CH aromatic), 2985 (CH aliphatic), 1660–1651 (3 C=O). ^1^H NMR (400 MHz, DMSO-*d*_*6*_) *δ* ppm: 12.24 (s, 1H, CONH, D_2_O exchangeable), 8.85 (s, 1H, H-2-quinoline), 8.58 (s, 1H, NH, D_2_O exchangeable), 8.52 (s, 1H, NH, D_2_O exchangeable), 7.93 (d, *J* = 9.0 Hz, 1H, H-5-quinoline), 7.80–7.68 (m, 2H, H-7 and H-8-quinoline), 7.63 (d, *J* = 8.6 Hz, 2H, Ar–H), 7.43 (d, *J* = 8.4 Hz, 2H, Ar–H), 7.33 (d, *J* = 8.0 Hz, 2H, Ar–H), 7.06 (d, *J* = 7.9 Hz, 2H, Ar–H), 2.23 (s, 3H, -CH_3_). ^13^C NMR (101 MHz, DMSO-*d*_*6*_) *δ* ppm: 175.46, 162.33, 152.71, 144.02, 137.24, 136.04, 135.55, 133.11, 130.64, 129.22 (2C), 122.22, 121.88, 121.63, 120.25 (2C), 118.89 (2C), 118.36 (2C), 110.27, 109.85, 109.62, 20.37. Anal. Calcd for C_24_H_19_FN_4_O_3_ (430.43): C, 66.97; H, 4.45; N, 13.02. Found: C, 66.71; H, 4.60; N, 13.28.

##### 6-Fluoro-4-oxo-*N*-(4-(3-(*m*-tolyl)ureido)phenyl)-1,4-dihydroquinoline-3-carboxamide (**10l**)

Yellow precipitate; Yield: 54.6%; M.p.: > 300 °C. IR (KBr, cm^−1^): 3414–3263 (4 NH), 3097 (CH aromatic), 2981 (CH aliphatic), 1670 (br., 3 C=O). ^1^H NMR (500 MHz, DMSO-*d*_*6*_) *δ* ppm: 12.29 (s, 1H, CONH, D_2_O exchangeable), 8.90 (s, 1H, H-2-quinoline), 8.62 (s, 1H, , NH, D_2_O exchangeable), 8.57 (s, 1H, , NH, D_2_O exchangeable), 7.96 (dd, *J* = 9.1, 3.0 Hz, 1H, , H-5-quinoline), 7.83 (dd, *J* = 9.1, 4.6 Hz, 1H, H-8-quinoline), 7.71 (td, *J* = 8.6, 3.0 Hz, 1H, H-7-quinoline), 7.63 (d, *J* = 8.9 Hz, 2H, Ar–H), 7.43 (d, *J* = 8.9 Hz, 2H, Ar–H), 7.30 (s, 1H, Ar–H), 7.22 (d, *J* = 7.7 Hz, 1H, Ar–H), 7.13 (t, *J* = 7.7 Hz, 1H, Ar–H), 6.77 (d, *J* = 7.4 Hz, 1H, Ar–H), 2.27 (s, 3H, -CH_3_). ^13^C NMR (101 MHz, DMSO-*d*_*6*_) *δ* ppm: 175.55, 162.36, 152.68, 146.83, 144.06, 139.74, 138.05, 136.00, 135.52, 133.16, 128.72, 122.64, 122.16, 120.31(2C), 118.99 (2C), 118.82, 115.48, 110.96, 110.31, 109.90, 109.68, 21.31. Anal. Calcd for C_24_H_19_FN_4_O_3_ (430.43): C, 66.97; H, 4.45; N, 13.02. Found: C, 66.88; H, 4.63; N, 13.19.

##### 6-Fluoro-4-oxo-*N*-(4-(3-(*o*-tolyl)ureido)phenyl)-1,4-dihydroquinoline-3 carboxamide (**10m**)

Pale yellow precipitate; Yield: 46.8%; M.p.: > 300 °C. IR (KBr, cm^−1^): 3271 (4 NH), 3059 (CH aromatic), 2978 (CH aliphatic), 1647 (br., 3 C=O). ^1^H NMR (400 MHz, DMSO-*d*_*6*_) *δ* ppm:12.26 (s, 1H, CONH, D_2_O exchangeable), 8.98 (s, 1H, NH, D_2_O exchangeable), 8.89 (s, 1H, H-2-quinoline), 7.95 (dd, *J* = 9.2, 3.0 Hz, 1H, H-5 quinoline), 7.88 (s, 1H, NH, D_2_O exchangeable), 7.85–7.82 (m, 2H, H-8-quinoline and Ar–H), 7.70 (td, *J* = 8.6, 3.0 Hz, 1H, H-7-quinoline,),7.64 (d, *J* = 9.0 Hz, 2H, Ar–H), 7.44 (d, *J* = 8.5 Hz, 2H, Ar–H), 7.18–7.12 (m, 2H, Ar–H), 6.92 (t, *J* = 7.4 Hz, 1H, Ar–H), 2.25 (s, 3H, -CH_3_). ^13^C NMR (101 MHz, DMSO-*d*_*6*_) *δ* ppm: 175.47, 162.31, 152.73, 144.09, 143.46, 137.49, 135.62, 133.03, 130.21, 127.50, 127.15, 126.19, 122.65, 122.27, 121.04, 120.27(2C), 118.71(2C), 115.46, 112.96, 110.25, 109.62, 17.91. Anal. Calcd for C_24_H_19_FN_4_O_3_ (430.43): C, 66.97; H, 4.45; N, 13.02. Found: C, 67.04; H, 4.61; N, 13.27.

##### 6-Fluoro-*N*-(4-(3-(4-fluorophenyl)ureido)phenyl)-4-oxo-1,4-dihydroquinoline-3-carboxamide (**10n**)

Yellow precipitate; Yield: 51%; M.p.: > 300 °C. IR (KBr, cm^−1^): 3375–3221 (4 NH), 3074 (CH aromatic), 1685–1651 (3 C=O). ^1^H NMR (500 MHz, DMSO-*d*_*6*_) *δ* ppm: 12.28 (s, 1H, CONH, D_2_O exchangeable), 8.88 (s, 1H, H-2-quinoline), 8.69 (s, 1H, NH, D_2_O exchangeable), 8.64 (s, 1H, 1NH, D_2_O exchangeable), 7.95–7.71 (m, 3H, H-5, H-7 and H-8-quinoline), 7.65 (m, 2H, Ar–H), 7.46 (m, 4H, Ar–H), 7.12 (m, 2H, Ar–H). ^13^C NMR (126 MHz, DMSO-*d*_*6*_) *δ* ppm: 175.46, 162.36, 152.74, 144.13, 136.16, 135.40, 133.24, 127.42, 122.28, 121.85, 120.24 (2C), 120.03 (2C), 119.97, 119.03 (2C), 115.37 (2C), 115.19, 110.27, 109.81, 109.63. Anal. Calcd for C_23_H_16_F_2_N_4_O_3_ (434.39): C, 63.59; H, 3.71; N, 12.90. Found: C, 63.72; H, 3.85; N, 13.18.

##### 6-Fluoro-4-oxo-*N*-(4-(3-(3-(trifluoromethyl)phenyl)ureido)phenyl)-1,4-dihydroquinoline-3-carboxamide (**10o**)

Yellow precipitate; Yield: 42%; M.p.: > 300 °C. IR (KBr, cm^−1^): 3271–3236 (4 NH), 3066 (CH aromatic), 1670–1650 (3 C=O). ^1^H NMR (500 MHz, DMSO-*d*_*6*_) *δ* ppm: 12.28 (s, 1H, CONH, D_2_O exchangeable), 9.04 (s, 1H, NH, D_2_O exchangeable), 8.88 (s, 1H, H-2-quinoline), 8.77 (s, 1H, NH, D_2_O exchangeable), 8.03 (s, 1H , Ar–H), 7.94 (dd, *J* = 9.2, 2.9 Hz, 1H, H-5-quinoline), 7.80 (dd, *J* = 9.1, 4.6 Hz, 1H, H-8-quinoline), 7.73–7.69 (m, 1H, H-7-quinoline), 7.65 (d, *J* = 8.4 Hz, 2H, Ar–H), 7.56 (d, *J* = 8.1 Hz, 1H, Ar–H), 7.51–7.48 (m, 1H, Ar–H), 7.45 (d, *J* = 8.5 Hz, 2H, Ar–H), 7.28 (d, *J* = 7.8 Hz, 1H, Ar–H). ^13^C NMR (126 MHz, DMSO-*d*_*6*_) *δ* ppm: 175.59, 162.46, 160.47, 158.53, 152.71, 144.15, 140.84, 136.11, 135.14, 133.61, 130.01, 127.46, 122.30, 122.24, 121.93, 120.33 (2C), 119.43 (2C), 118.10, 114.28, 110.38, 109.93, 109.75. Anal. Calcd for C_24_H_16_F_4_N_4_O_3_ (484.40): C, 59.51; H, 3.33; N, 11.57. Found: C, 59.78; H, 3.45; N, 11.

##### 6-Fluoro-*N*-(4-(3-(4-isopropylphenyl)ureido)phenyl)-4-oxo-1,4-dihydroquinoline-3-carboxamide (**10p**)

Buff precipitate; Yield: 64%, M.p.: > 300 °C. IR (KBr, cm^−1^): 3309–3224 (4 NH), 3055 (CH aromatic), 2954 (CH aliphatic), 1680–1643 (3 C=O). ^1^H NMR (400 MHz, DMSO-*d*_*6*_) *δ* ppm: 12.25 (s, 1H, CONH, D_2_O exchangeable), 8.88 (s, 1H, H-2-quinoline), 8.57 (s, 1H, NH, D_2_O exchangeable), 8.52 (s, 1H, NH, D_2_O exchangeable), 7.95 (dd, *J* = 9.1, 3.0 Hz, 1H, H-5-quinoline), 7.82 (dd, *J* = 9.1, 4.7 Hz, 1H, H-8-quinoline), 7.70 (dt, *J* = 10.3, 5.1 Hz, 1H, H-7-quinoline), 7.63 (d, *J* = 8.4 Hz, 2H, Ar–H), 7.43 (d, *J* = 8.5 Hz, 2H, Ar–H), 7.35 (d, *J* = 8.2 Hz, 2H, Ar–H), 7.13 (d, *J* = 8.1 Hz, 2H, Ar–H), 2.84- 2.81(m, 1H, -CH(CH_3_)_2_), 1.19–1.17 (d, *J* = 6.9 Hz, 6H, -CH(CH_3_)_2_). ^13^C NMR (126 MHz, DMSO-*d*_*6*_) *δ* ppm: 175.42, 162.24, 152.62, 144.00, 141.81, 137.42, 135.96, 135.50, 133.02, 127.34, 126.44 (2C), 122.10, 121.86, 120.18 (2C), 118.80 (2C), 118.38 (2C), 110.24, 109.77, 109.76, 32.74, 23.99 (2C). Anal. Calcd for C_26_H_23_FN_4_O_3_ (458.48): C, 68.11; H, 5.06; N, 12.22. Found: C, 67.94; H, 5.23; N, 12.46.

### Biological evaluation

#### Enzyme inhibition assay versus VEGFR-2

The VEGFR-2 Kinase Assay Kit was obtained to measure VEGFR-2 kinase activity using Kinase-Glo MAX as a detection reagent. The VEGFR-2 Kinase Assay Kit (BPS Bioscience, Catalog # 40325) is received in a convenient 96-well format, accompanied with purified recombinant VEGFR-2 enzyme, VEGFR-2 substrate in addition to ATP and Kinase Buffer 1 to be used for 100 enzyme reactions^2,34^. In this assay, the kinase activity is obtained through measuring the amount of ATP remaining in solution following a kinase reaction as a type of luminescence kinase assay. There is a direct correlation between luminescent signal and the amount of ATP present with an inverse correlation with the amount of kinase activity. The assay protocol starts with thawing 5 × Kinase Buffer 1, ATP and 50 × PTK substrate. The master mixture was prepared (25 mL per well) as follows: N wells x (6 µL 5 × Kinase Buffer 1 + 1 µL ATP (500 µM) + 1 µL 50 × PTK substrate + 17 µL water). Addition of 5 µl of Inhibitor solution for the wells named as “Test Inhibitor”. 5 μL of the same solution without the addition of inhibitor (Inhibitor buffer) was added for both the “Positive Control" and “Blank”. Preparation of 3 ml of 1 × Kinase Buffer 1 by mixing 600 µL of 5 × Kinase Buffer 1 with 2400 µl water; these 3 ml of 1 × Kinase Buffer 1 were enough to perform 100 reactions. Add 20 μL of 1 × Kinase Buffer 1 to the well designated for "Blank". Thawing of VEGFR-2 enzyme on ice, followed by calculating the quantity of VEGFR-2 needed for this test then diluting the enzyme to 1 ng/µL using 1 × Kinase Buffer 1. Initiating the reaction by the addition of 20 µL of the diluted VEGFR-2 enzyme to the “Positive Control” wells and also "Test Inhibitor Control" wells then incubation at 30 °C for 45 min. This was followed by the addition of 50 µL of Kinase-Glo Max reagent in all the wells within 45 min. Covering the plates using an aluminum foil to be incubated for 15 min at room temperature. At the end, luminescence was measured using the Bioline ELISA microplate reader at wave length 450 nm. The computer software Graphpad Prism was used for analyzing the luminescence data. The difference between luminescence intensities in the absence of VEGFR (Lu_t_) and in the presence of VEGFR (Luc) was defined as 100% activity (Lu_t_ – Lu_c_). Using luminescence signal (Lu) in the presence of the compound, % activity was calculated as: % activity = {(Lu_t_—Lu)/(Lu_t_—Lu_c_) × 100%, where Lu = the luminescence intensity in the presence of the compound. % Inhibition was calculated as: % inhibition = 100 (%) ˗ % activity. IC_50_ determination for target compounds against VEGFR-2 was calculated. The values of % activity versus a series of compound concentrations (10 μM, 1 μM, 0.1 μM and 0.01 μM) were then plotted using non-linear regression analysis of Sigmoidal dose–response curve generated with the equation: Y = B + (T-B)/1 + 10((LogEC50-X) × Hill Slope), where Y = percent activity, B = minimum percent activity, T = maximum percent activity, X = logarithm of compound and Hill Slope = slope factor or Hill coefficient. The IC_50_ value was determined by the concentration causing a half-maximal percent activity^61,63,64^.

#### Cytotoxicity against hepatocellular carcinoma (HepG2)

Using the 3-(4,5-dimethylthiazol-2-yl)-2,5-diphenyltetrazolium bromide (MTT) method; the cytotoxic effects of the novel quinolone derivatives were investigated in vitro against human hepatocellular carcinoma (HepG2) cells (purchased from ATCC). MTT method is an accurate method that provides reproducible results. Sorafenib was used as a reference standard. The solutions of MTT were prepared in medium or balanced salt solutions without phenol red to produce a yellowish color. The principle of this assay counts on that the mitochondrial dehydrogenases of viable cells cleave the tetrazolium ring, affording purple formazan crystals which are insoluble in aqueous solutions. The produced crystals were then dissolved in acidified isopropanol. Spectrophotometric measurement of the produced purple solution reflecting the degree of cytotoxicity caused by the test compounds^65^. A blank containing complete medium without cells should be included. Several concentrations (0.4, 1.6, 6.25, 25 and 100 mM) of the screened derivatives as well as sorafenib were added to cells that were then incubated at 37 °C for 48 h. Cultures were removed from incubator into laminar flow hood or other sterile work area where each vial of MTT [M-5655] is reconstituted to be used with 3 ml of medium or balanced salt solution without phenol red and serum. Adding the reconstituted MTT in an amount equal to 10% of the culture medium volume. After an incubation period of 2–4 h depending on cell type and maximum cell density; the produced formazan crystals were dissolved by adding an amount of MTT Solubilization Solution [M-8910] equal to the original culture medium volume. Measuring the intensity of the produced color via ROBONIK P2000 spectrophotometer at a wavelength of 570 nm. Finally, measuring the background absorbance of multi-well plates at 690 nm and subtract from the 570 nm measurement. The percentage of cell viability and drug concentration were used to construct the survival curve of HepG2 for each of the tested compounds. IC_50_ values (the concentration that causes 50% inhibition of cell viability) for the screened derivatives as well as the reference drug compound sorafenib were obtained in micromolar as the average of three independent runs ± SE^66,67^.

#### Cytotoxicity screening against normal liver cell line

The cytotoxic activities of compounds **10i** and **10o** were tested against Transformed Human Liver Epithelial-2 normal cell line (THLE-2) (purchased from ATCC) using Sorafenib as a reference drug using MTT assay to measure the viability of the cells^68^. As previously described; multi-well plates were used to treat grown cells with several concentrations of the screened derivatives then incubated for 48 h at 37 °C. The produced formazan was measured by means of the spectrophotometer ROBONIK P2000 at a wavelength of 570 nm. The Cytotoxic concentration values (CC_50_) of the assessed compounds were reported as the mean of three independent runs ± SE^69^.

#### Apoptosis assay

Apoptosis assay was performed as previously described^70^. Briefly, the assay used Annexin V-FITC apoptosis detection kit (Abcam Inc., Cambridge Science Park, Cambridge, UK) along with two fluorescent channels flow cytometry. Compound **10i**, having the lowest IC_50_ value against the HepG2 cells, was applied to the HepG2 cells at its IC_50_ concentration and cells were left to incubate for 48 h. About 10^5^ cells were collected by trypsinization and washed for two consecutive times with ice-cold PBS. Thereafter, a volume of 0.5 mL of Annexin V-FITC/PI solution was added to stain the cells and they were kept for 30 min in a dark place at standard room temperature. Then, cells were moved to the Novocyte ACEA flow cytometer (ACEA Biosciences Inc., San Diego, CA, USA) where FL2 and FL1 detectors were selected for determination of PI and FITC signals. A query of 12,000 events was recommended for each sample. The ACEA NovoExpres software (ACEA Biosciences Inc., San Diego, CA, USA) was deployed for quadrant analysis.

#### Cell cycle analysis

The assay was performed as previously mentioned^70^. In brief, HepG2 cells were pre-incubated with compound **10i** at its IC_50_ concentration for 48 h. Then, about 10^5^ cells were attained by trypsinization and washed with phosphate buffer saline (PBS). This was followed by a resuspension step with 60% ice-cold ethanol and incubation till fixation occurs. The fixed cells were washed again and resuspended in a buffer that included 50 μg/mL RNAase A and 10 μg/mL propidium iodide. Finally, cells were incubated at 37 °C in the dark for 20 min and analyzed for the cell cycle kinetics using flow cytometry where FL2 (λex/em 535/617 nm) was used as the signal detector (ACEA Novocyt flowcytometer, ACEA Biosciences Inc., San Diego, CA, USA). A query of 12,000 events was recommended for each sample. ACEA NovoExpress software (ACEA Biosciences Inc., San Diego, CA, USA) was used for analysis.

#### RT-qPCR

##### RNA extraction

Total RNA was isolated from both **10i**-treated and untreated HepG2 cell line pellets using Qiagen RNeasy Mini kit Cat# No. 74104 according to the manufacturer’s guidelines. Then, quantification and quality assessment of the isolated RNA samples were determined by the Nanodrop spectrophotometer at A230, A260 and A280.

##### cDNA synthesis

cDNA synthesis was performed for RNA using the Promega cDNA Synthesis AMV Reverse Transcriptase Kit (cat# M5108) according to the manufacturer’s instructions. The reaction components were added as shown in Table 9. The cDNA synthesis was performed on Bio-Rad 100 Thermal cycler.

**Table 9:** cDNA synthesis reaction components.

Reaction component	Amount
**RNA**	1 µg
**Random Hexamers**	2.5 µL (from 100 µM stock)
**5X Enzyme Buffer**	5 µL
**dNTP**	1.7 µL (from 40 mM stock)
**AMV-RT (20U)**	2 µL
**RNase free water**	q.s. (quantity sufficient)
**Total volume**	25 µL

##### qPCR

Primers for the VEGFR-2 gene were designed and synthesized followed by primer validation and optimization of PCR amplification conditions. Primers used are presented in Table 10. RT-qPCR expression analysis for the required genes (ß-actin and VEGFR-2) has been done using the Qiagen Quanti Nova SYBR Green PCR Kit (cat # 208052). The reaction components are clearly demonstrated in Table 11. qPCR was performed on Rotor-Gene Q -Qiagen Real-time PCR thermal cycler.

**Table 10:** Primers used in RT-qPCR.

Primer ID	Primer sequence (5′–3′)
**β-actin F**	CACCATTGGCAATGAGCGGTTC
**β-actin R**	AGGTCTTTGCGGATGTCCACGT
**VEGFR-2 F**	GGAACCTCACTATCCGCAGAGT
**VEGFR-2 R**	CCAAGTTCGTCTTTTCCTGGGC

**Table 11:** qPCR reaction components.

qPCR reaction component	Amount
**Qiagen Master Mix 2X**	10 µL
**F primer**	1 µL (from 10 µM)
**R primer**	1 µL (from 10 µM)
**cDNA**	100 ng
**Complete with RNase free H**_**2**_**O**	q.s. (quantity sufficient)
**Total volume**	20 µL

#### Western blotting

Cells were harvested and washed with phosphate buffer saline (PBS). The cells were then lysed in 10 mM Tris–HCl, 100 mM NaCl, 0.5% Triton X-100, pH 7.6 with EDTA-free Protease Inhibitor Cocktail. This was followed by a centrifugation step at 13,000*g* for 20 min at 4 °C and direct transfer to sodium dodecyl sulfate–polyacrylamide gel (SDS–PAGE). Western blot analysis was performed using primary antibodies against the target proteins and HRP-conjugated secondary antibodies (Table 12). The chemiluminescence reaction was performed using the ECL western blot HRP substrate (Pierce, Thermo Fisher Scientific). Finally, densitometric quantification of Western blot protein bands were performed with IMAGEJ Software.

**Table 12:** Antibodies used for Western blot analysis.

Target	Company	Species	Dilution	Catalog No.
**Caspase 7**	Cell Signaling	Rabbit	1:1000	9492 T
**Bax**	Cell Signaling	Rabbit	1:1000	2772 T
**β-Actin**	Cell Signaling	Rabbit	1:1000	4970S
**Anti-rabbit IgG, HRP-linked**	Cell Signaling	Goat	1:3000	7074P2

### Molecular docking study

All molecular modelling calculations and docking studies were performed using the software “Molecular Operating Environment” (MOE) version 2019.01. Docking was performed using alpha triangle placement method, poses were prioritized based on affinity London dG scoring and refinement of the results were done using forcefield. Preparation of the downloaded crystal structure of the target protein available at the Protein Data Bank, http://www.rcsb.org/pdb (PDB ID: 4ASD) starting with removing of water molecules followed by energy minimization and 3D protonation of the amino acids. Validation of the docking process was ensured through re-docking of the co-crystallized ligand (Sorafenib) and the obtained RMSD was found to be less than 1. The hydrogen bond interactions formed by sorafenib within the active site of the protein were reported to be Glu 885, Cys 917 and Asp 1046. A database containing the newly synthesized compounds was created to be used in the docking process. All the compounds were prepared before molecular docking studies by 3D protonation, partial charges addition and energy minimization. The previous measures were considered, and docking was performed.

### Supplementary Information

Supplementary Information.

## Data Availability

All data generated or analyzed during this study are included in this published article and its Supplementary Information files). All datasets are available from the corresponding author on reasonable request.
