# Supplementary material for: New proapoptotic chemotherapeutic agents based on the quinolone-3-carboxamide scaffold acting by VEGFR-2 inhibition
Source: Sci Rep. 2023 Jul 13;13:11346. doi: 10.1038/s41598-023-38264-w (PMC10444817; doi:10.1038/s41598-023-38264-w)
Supplement: Supplementary file 1 — Supplementary Information. [file 41598_2023_38264_MOESM1_ESM.pdf]

Design and synthesis of new quinolone-3-carboxamide derivatives as VEGFR-2 inhibitors and proapoptotic chemotherapeutic agents

Zeinab S. El-Fakharany [a], Yassin M. Nissan [a,b], Nada K. Sedky [c], Reem K. Arafa [d,e,*], Sahar M. Abou-Seri [b,*]

[a] *Department of Pharmaceutical Chemistry, Faculty of Pharmacy, MSA university.*
[b] *Department of Pharmaceutical Chemistry, Faculty of Pharmacy, Cairo University, Kasr El-Aini Street, 11562, Cairo, Egypt*
[c] *Department of Biochemistry, School of Life and Medical Sciences, University of Hertfordshire Hosted by Global Academic Foundation, New Administrative Capital, Cairo, Egypt*
[d] *Biomedical Sciences Program, University of Science and Technology, Zewail City of Science and Technology, 12578, Cairo, Egypt*
[e] *Drug Design and Discovery Lab, Zewail City of Science and Technology, 12578, Cairo, Egypt*

***Corresponding Authors**

Sahar Mahmoud Abou-Seri

Professor of Pharmaceutical Chemistry
Faculty of Pharmacy, Cairo University
Kasr El-Aini Street, Cairo, Egypt, 11562
Mobile: +2-01010640490
E-mail: sahar.shaarawy@pharma.cu.edu.eg

Reem K. Arafa
Professor of Biomedical Sciences
University of Science and Technology
Zewail City of Science and Technology
October Gardens, 6th of October City, Giza, Egypt, 12578
Mobile: +2-01002074028
E-mail: rkhidr@zewailcity.edu.eg

10a

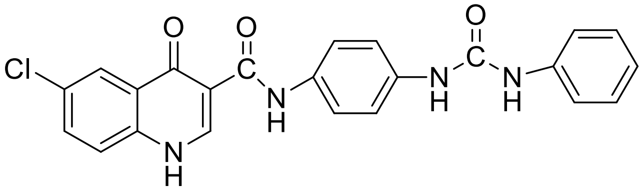

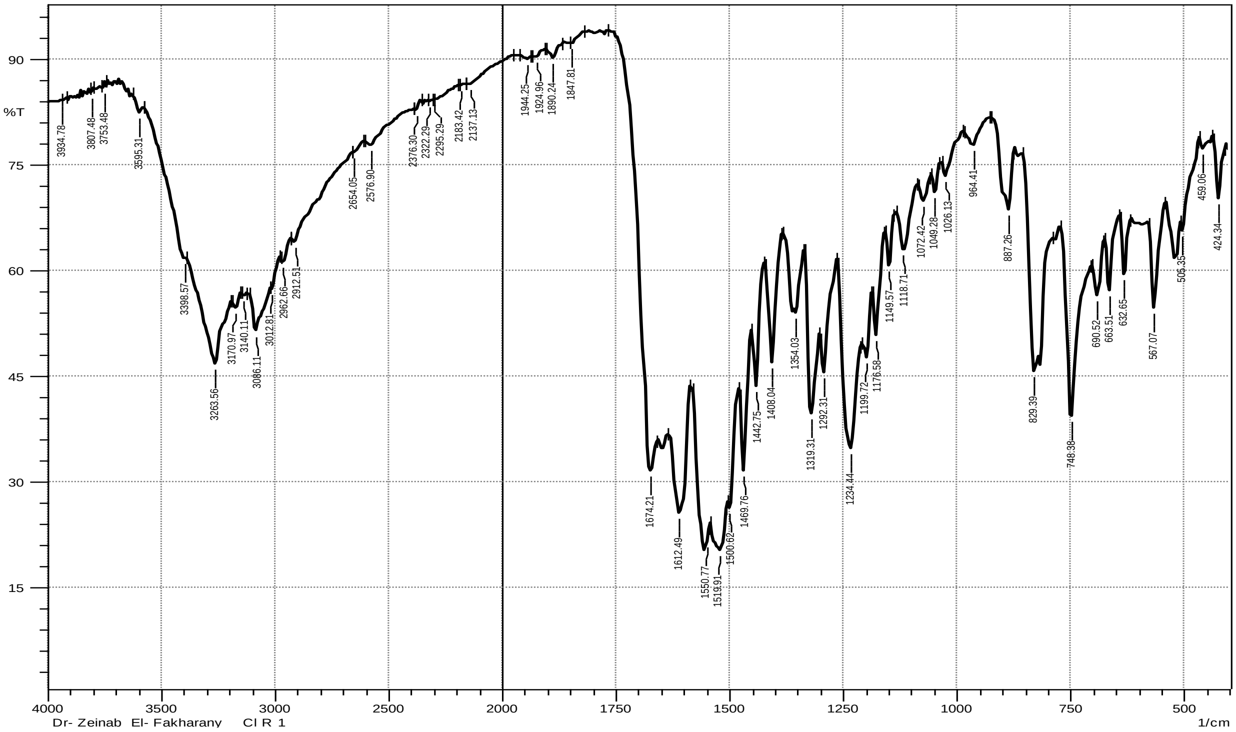

Fig S1: IR of **10a**

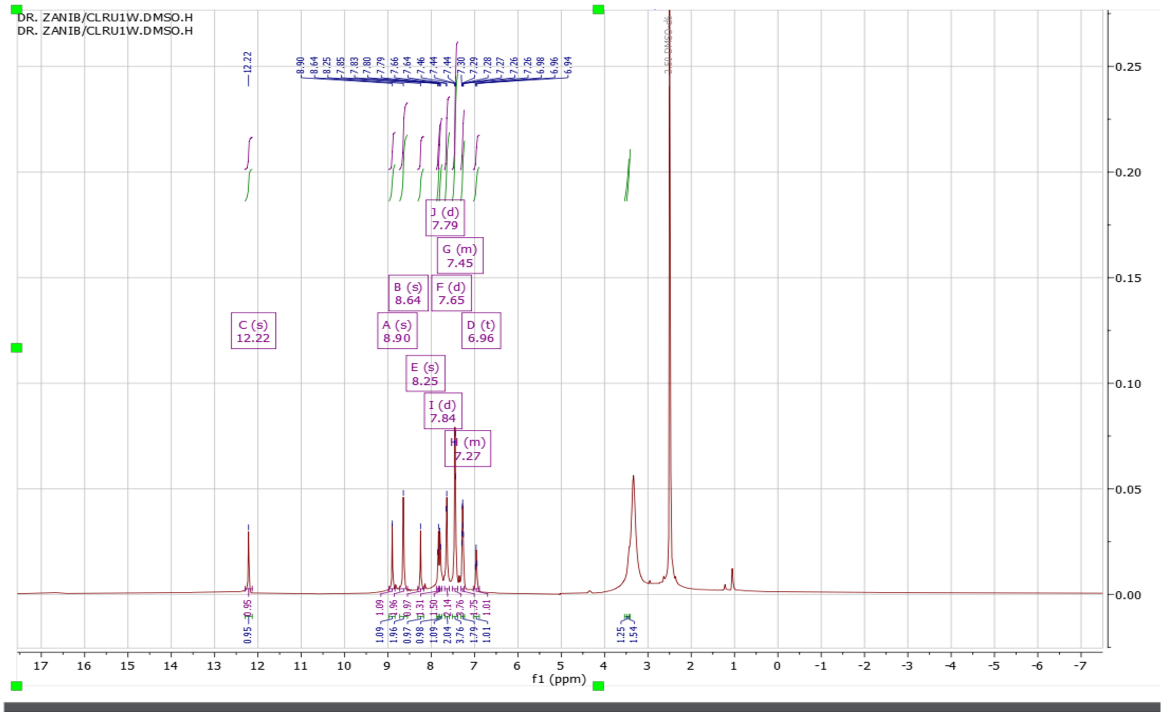

Fig S2: 1HNMR of **10a**

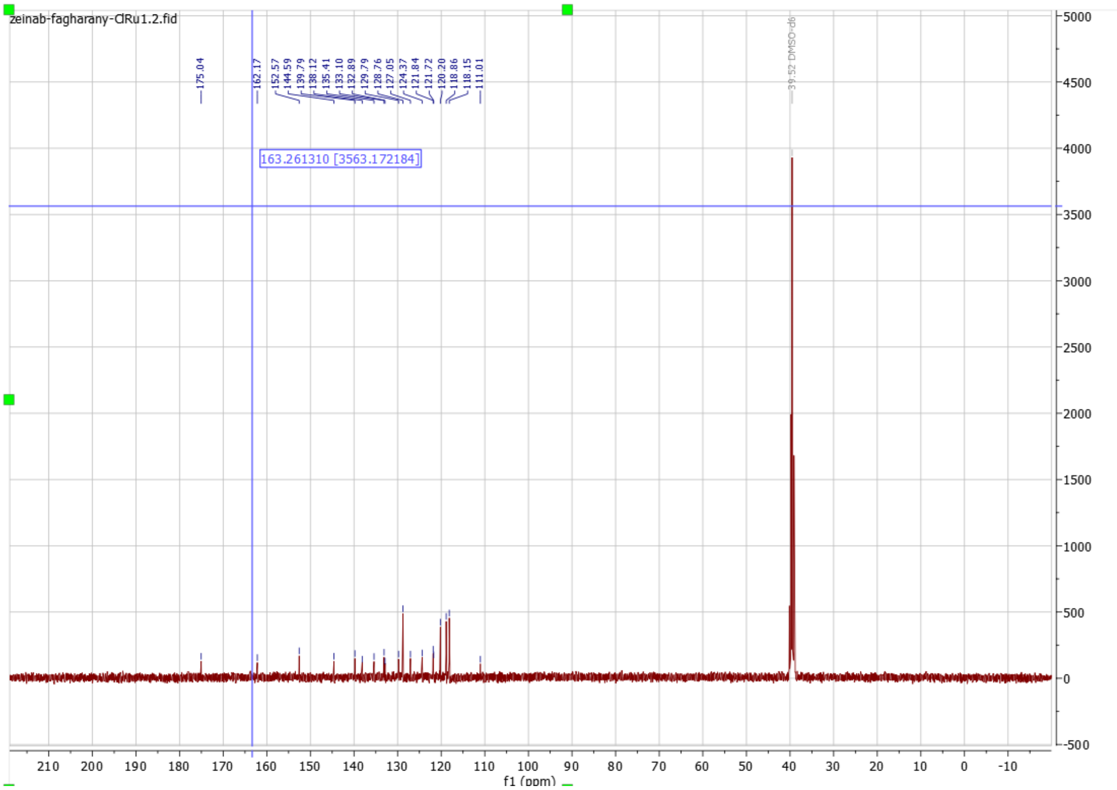

Fig S3: 13CNMR of **10a**

10b

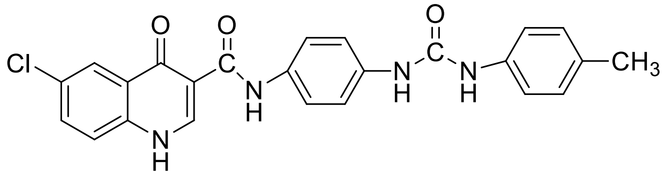

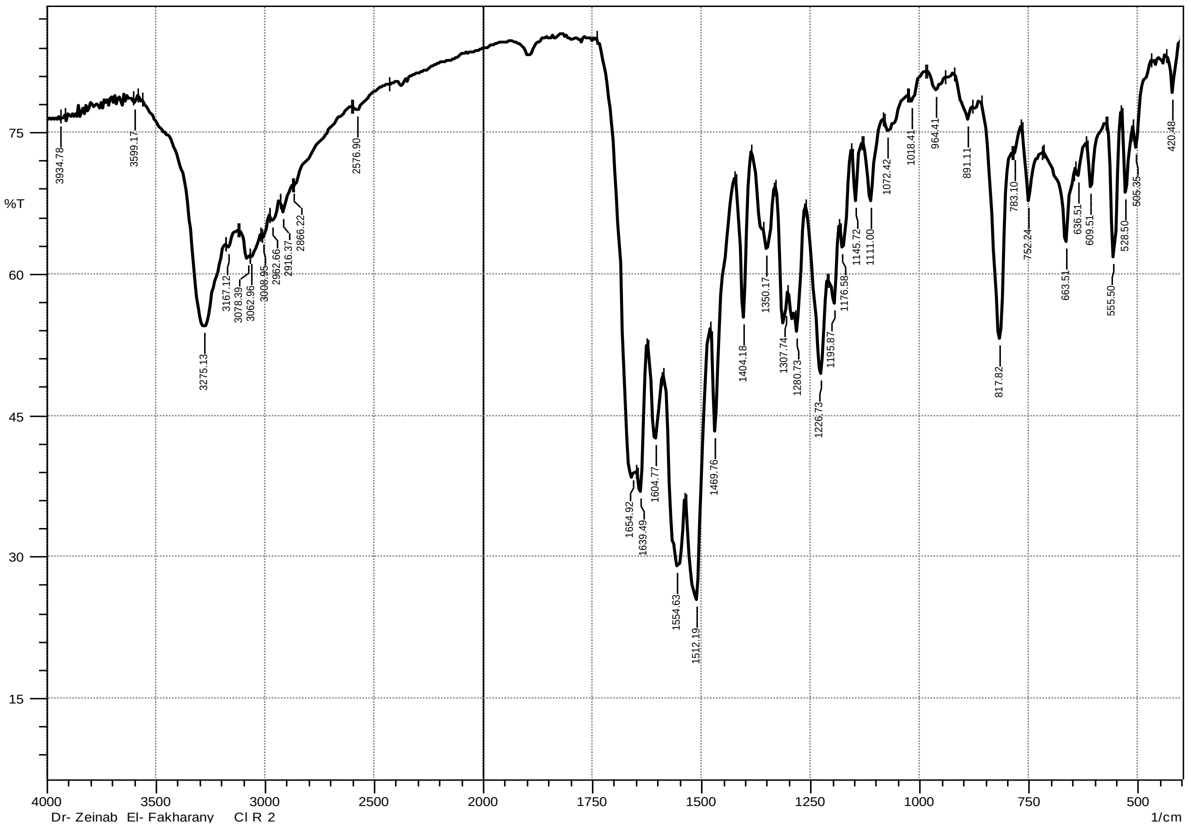

Fig S4: IR of **10b**

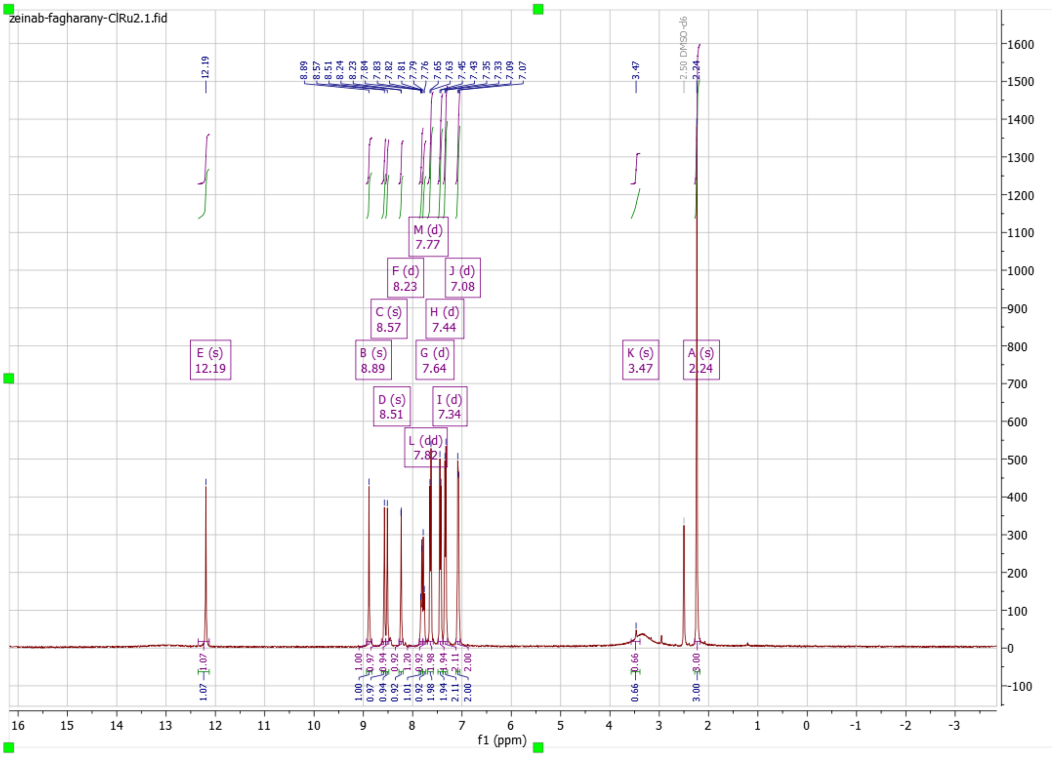

Fig S5: 1HNMR of **10b**

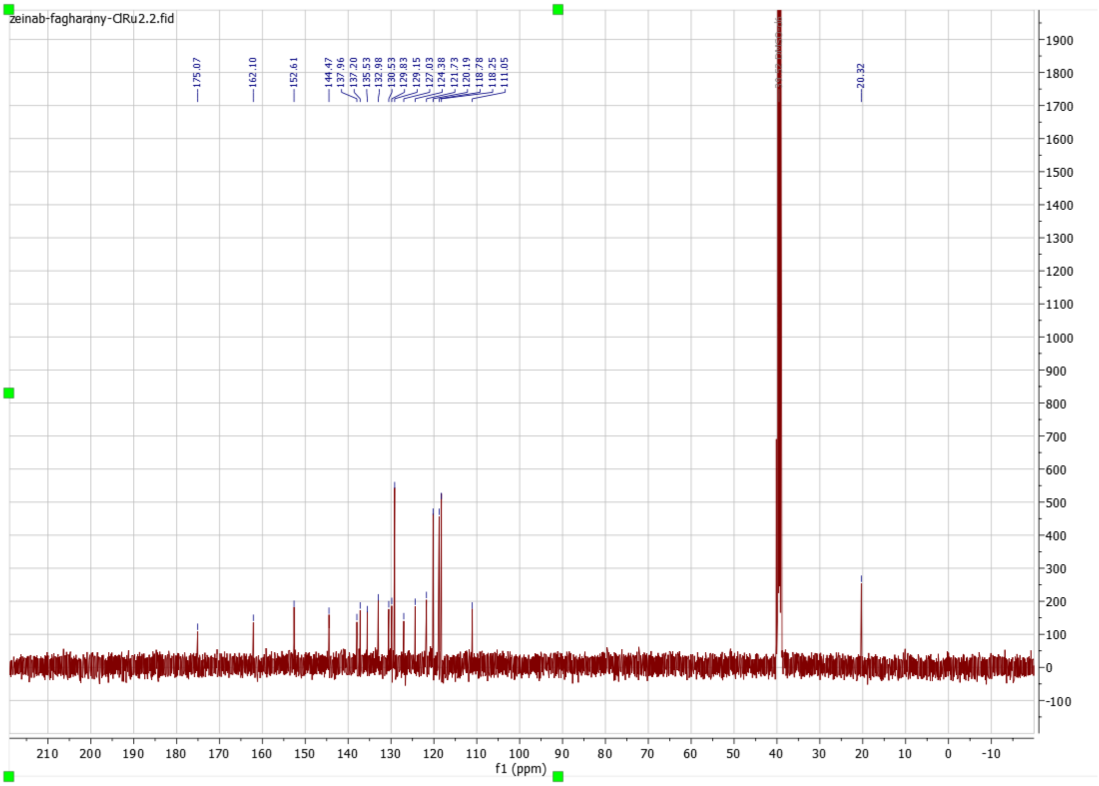

Fig S6: 13CNMR of **10b**

10c

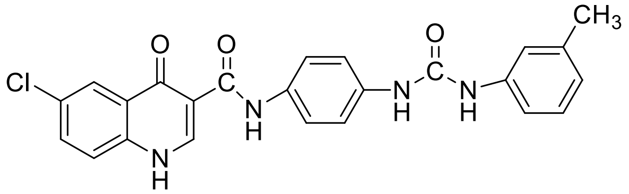

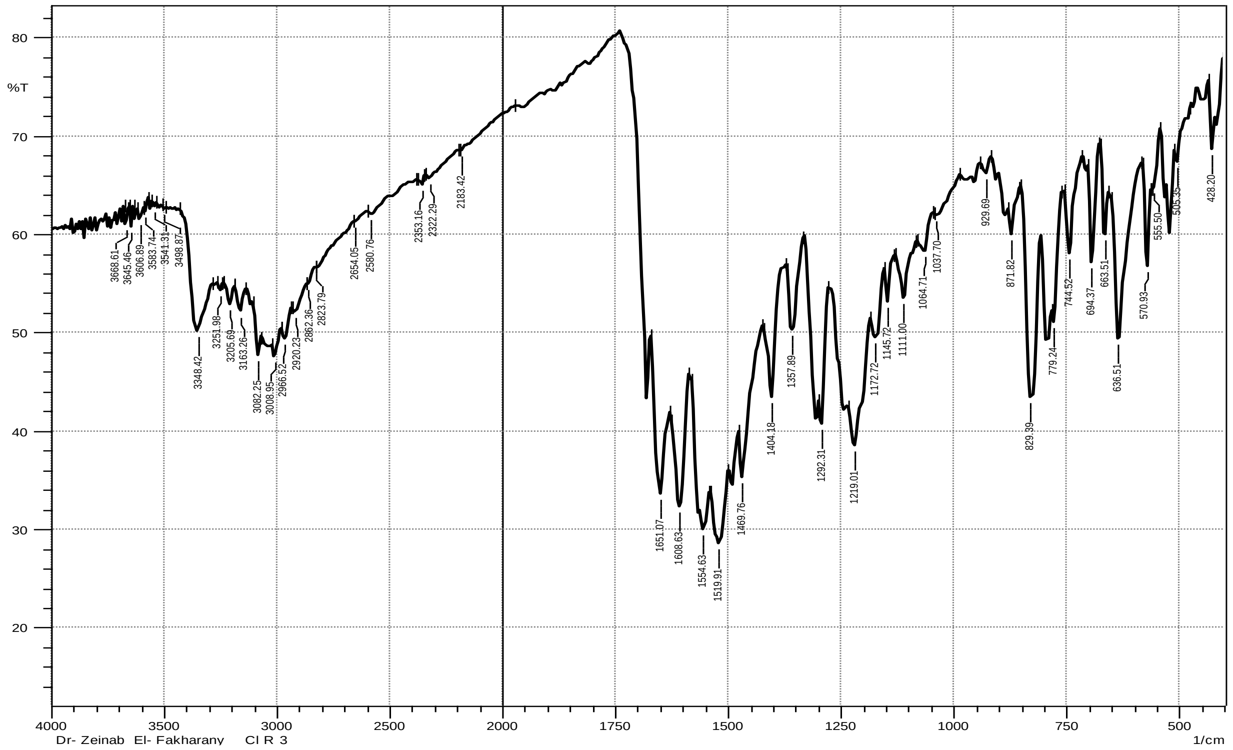

Fig S7: IR of **10c**

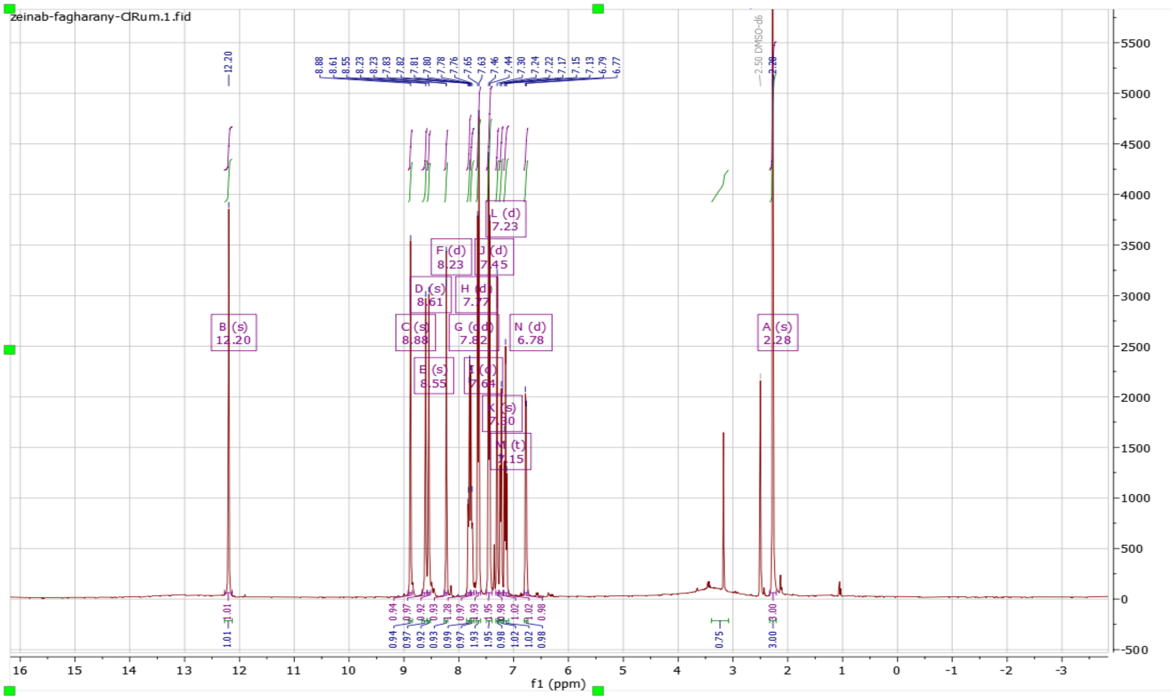

Fig S8: 1HNMR of **10c**

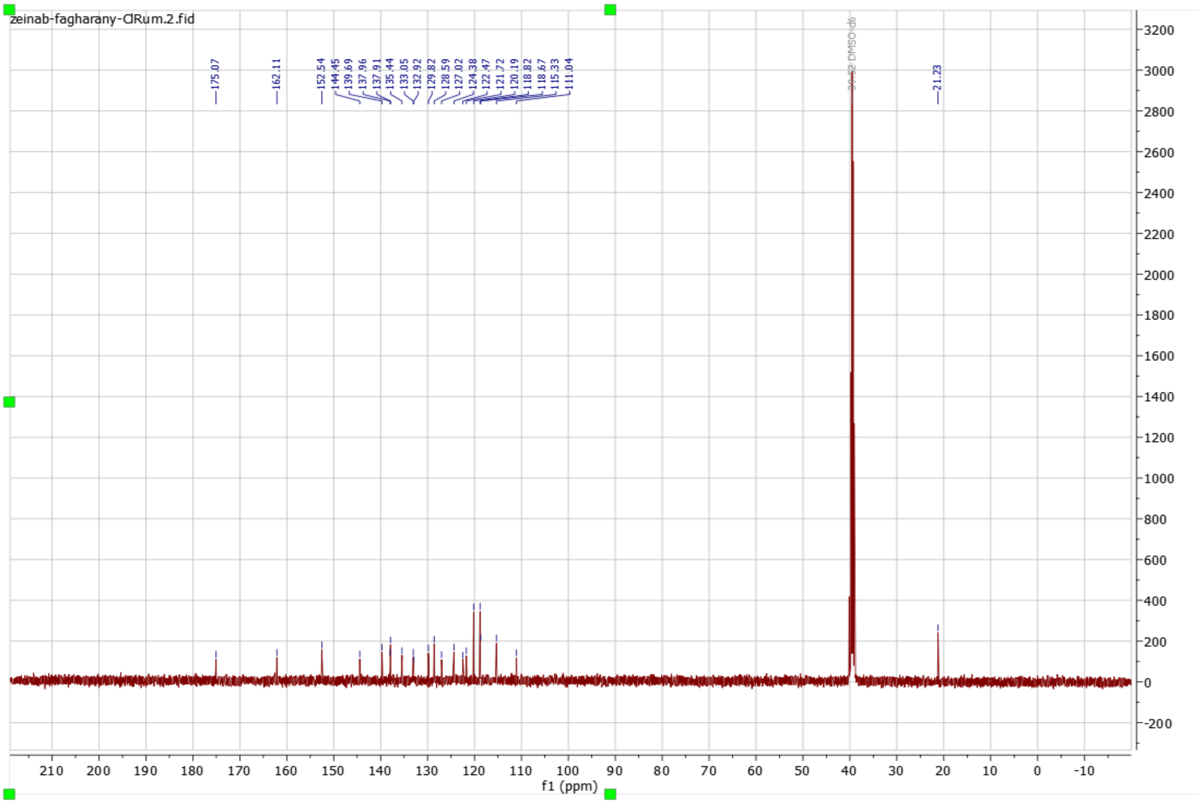

Fig S9: 13CNMR of **10c**

10d

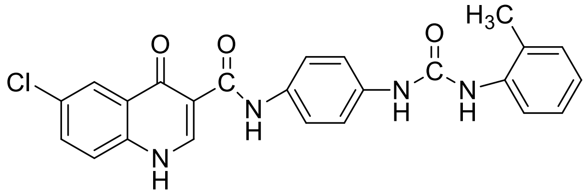

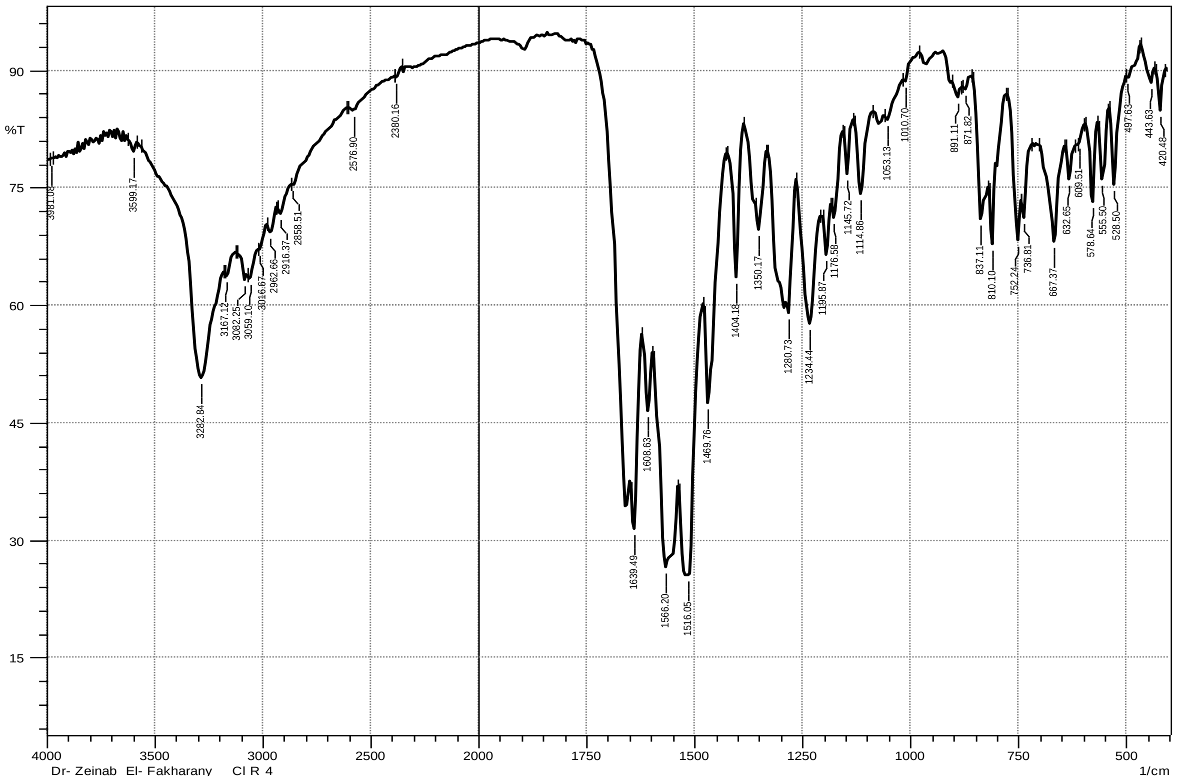

Fig S10: IR of **10d**

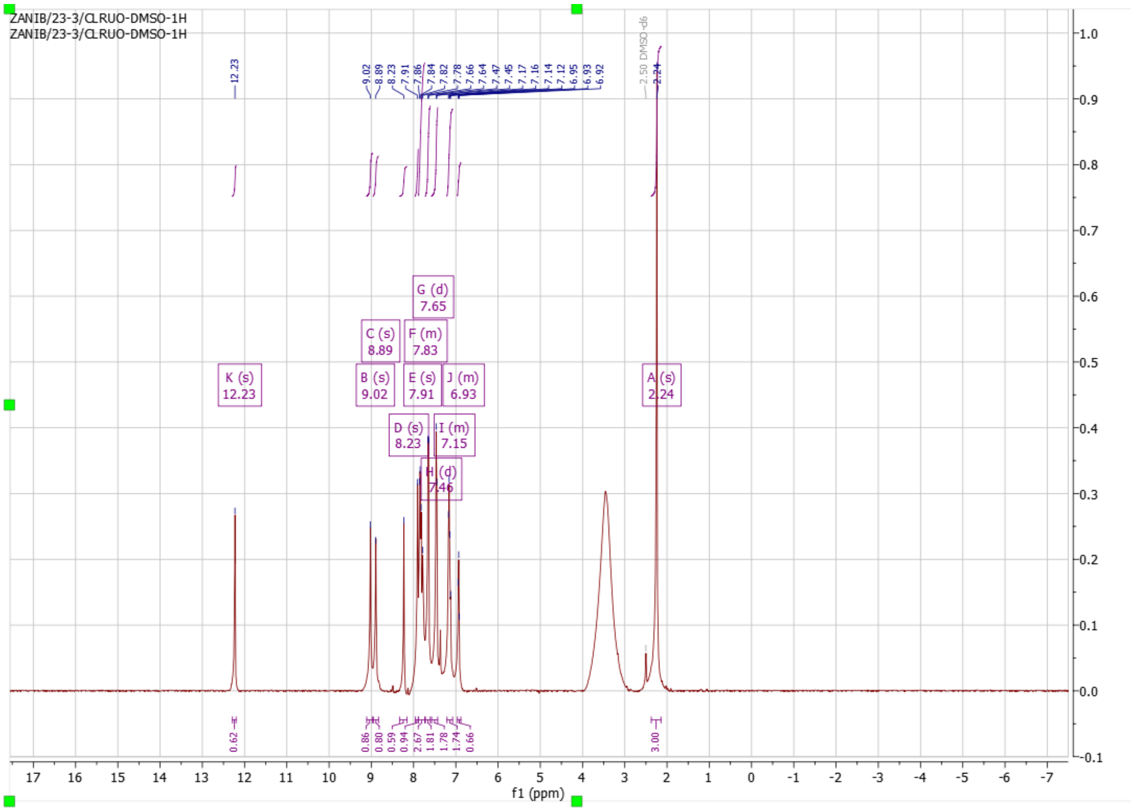

Fig S11: 1HNMR of **10d**

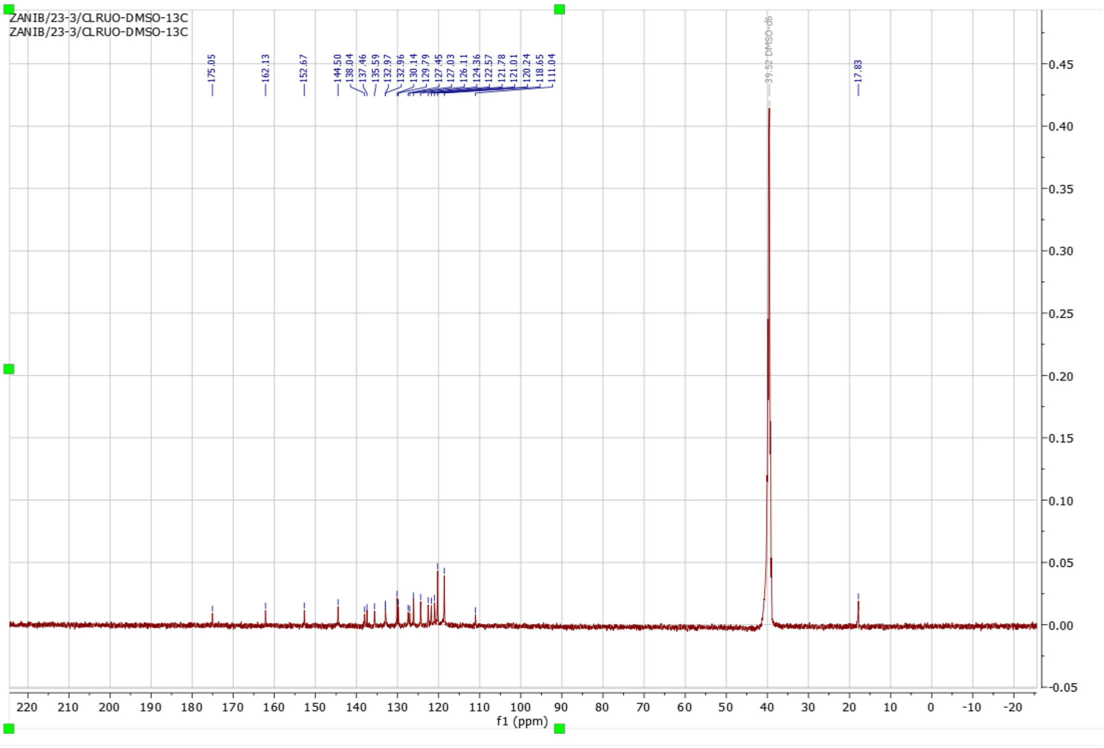

Fig S12: 13CNMR of **10d**

10e

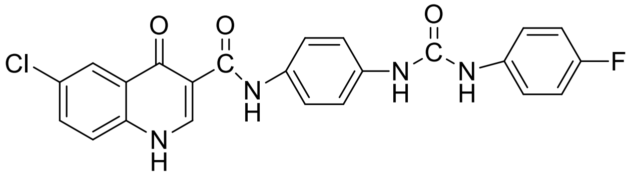

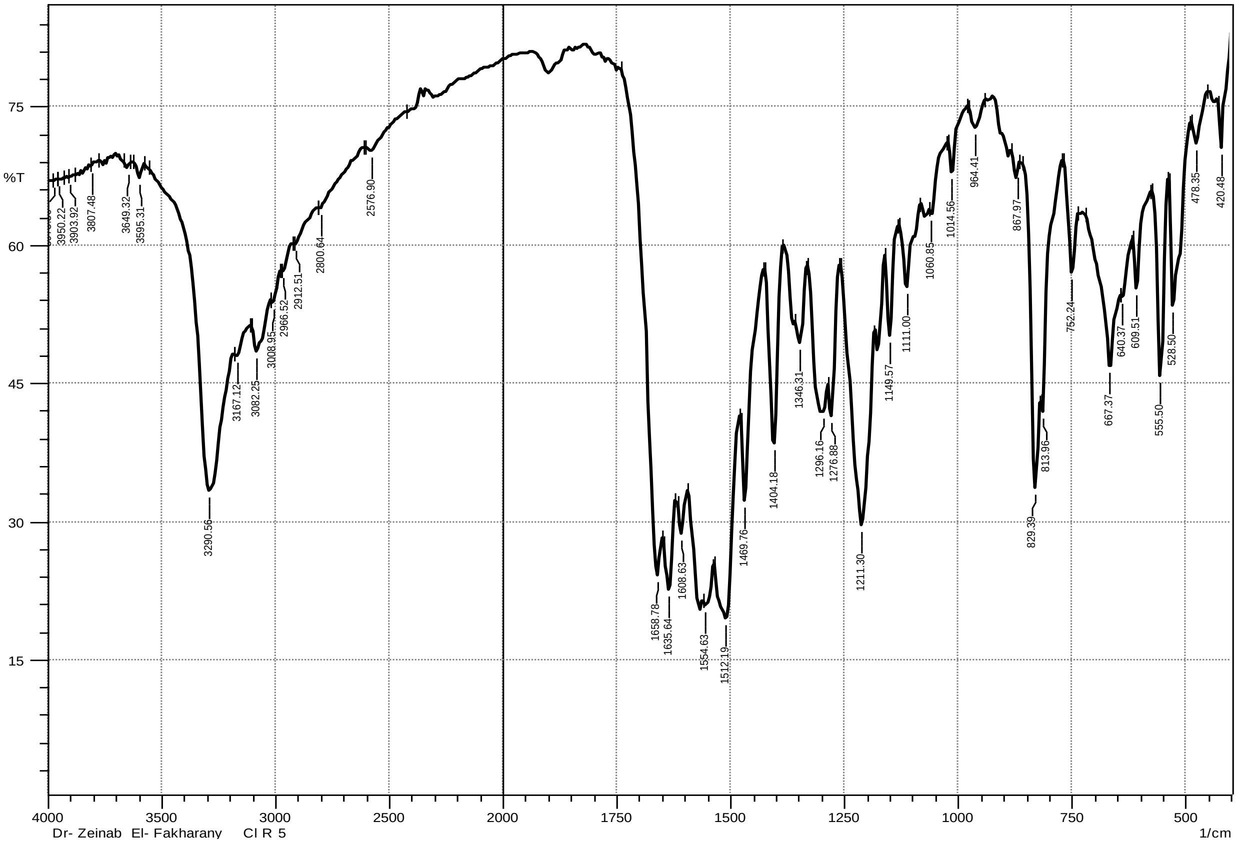

Fig S13: IR of **10e**

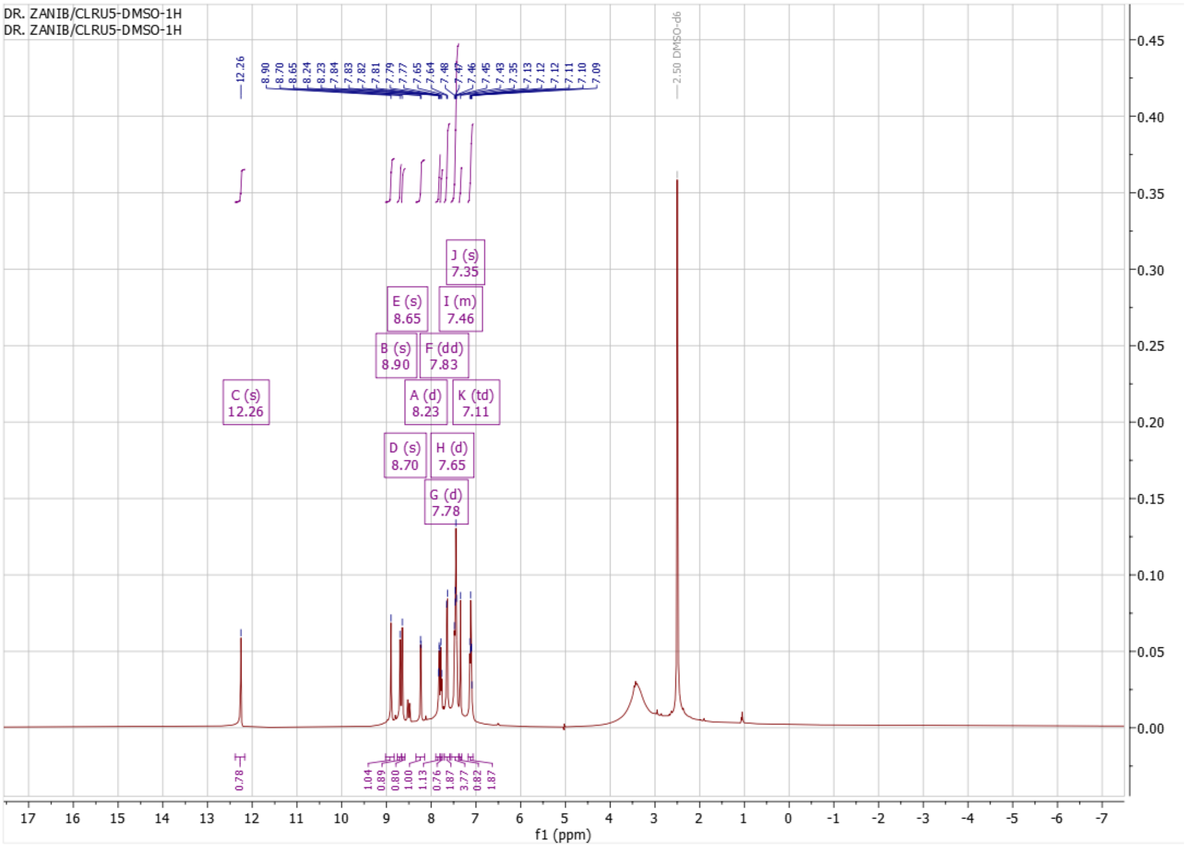

Fig S14: 1HNMR of **10e**

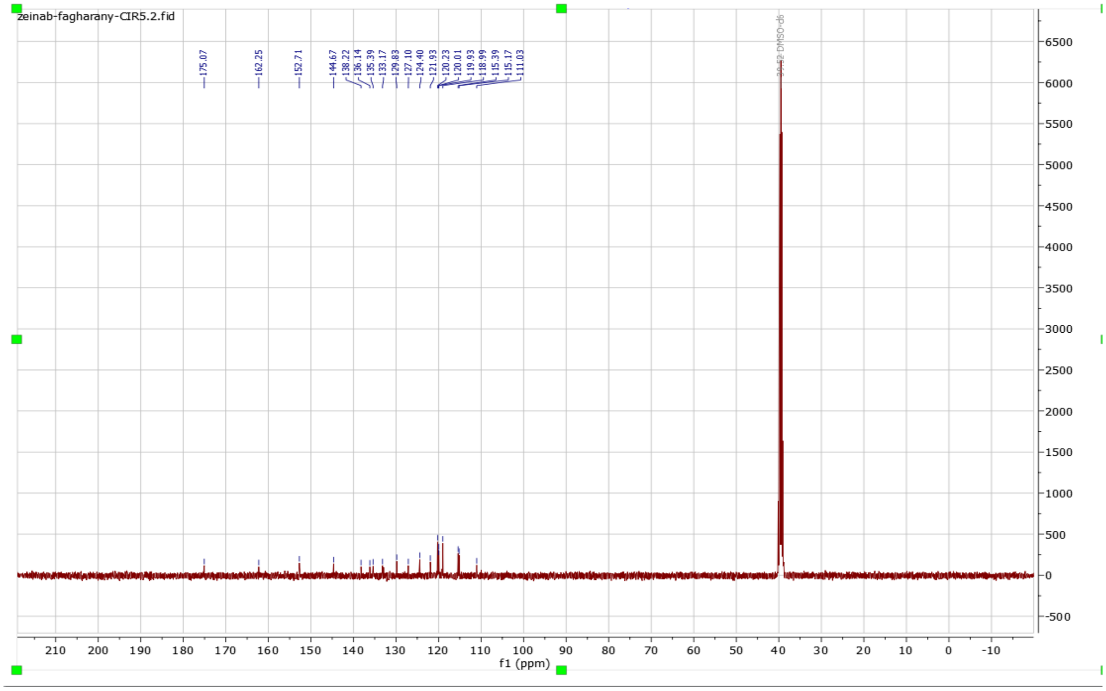

Fig S15: 13CNMR of **10e**

10g

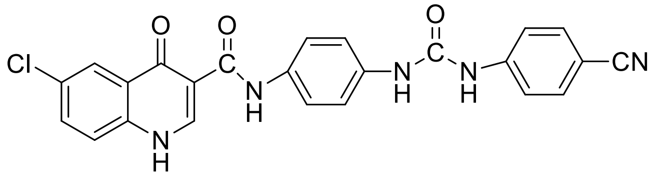

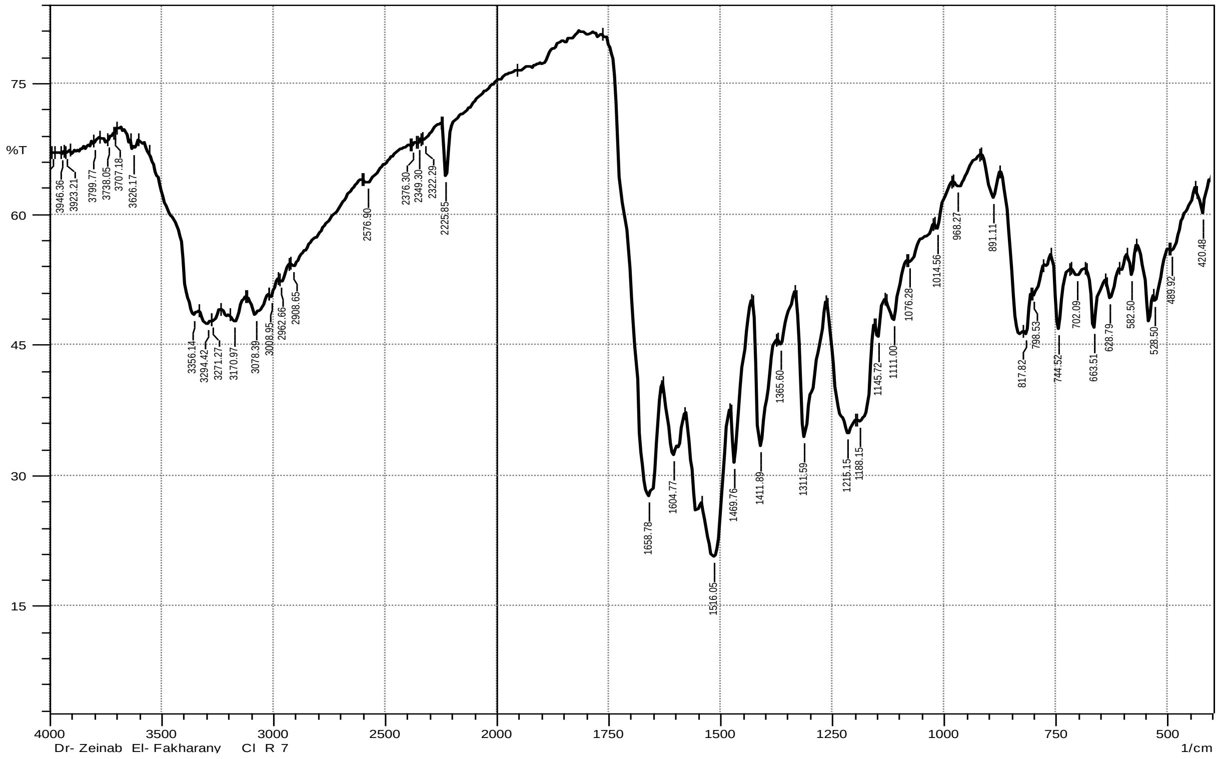

Fig S16: IR of **10g**

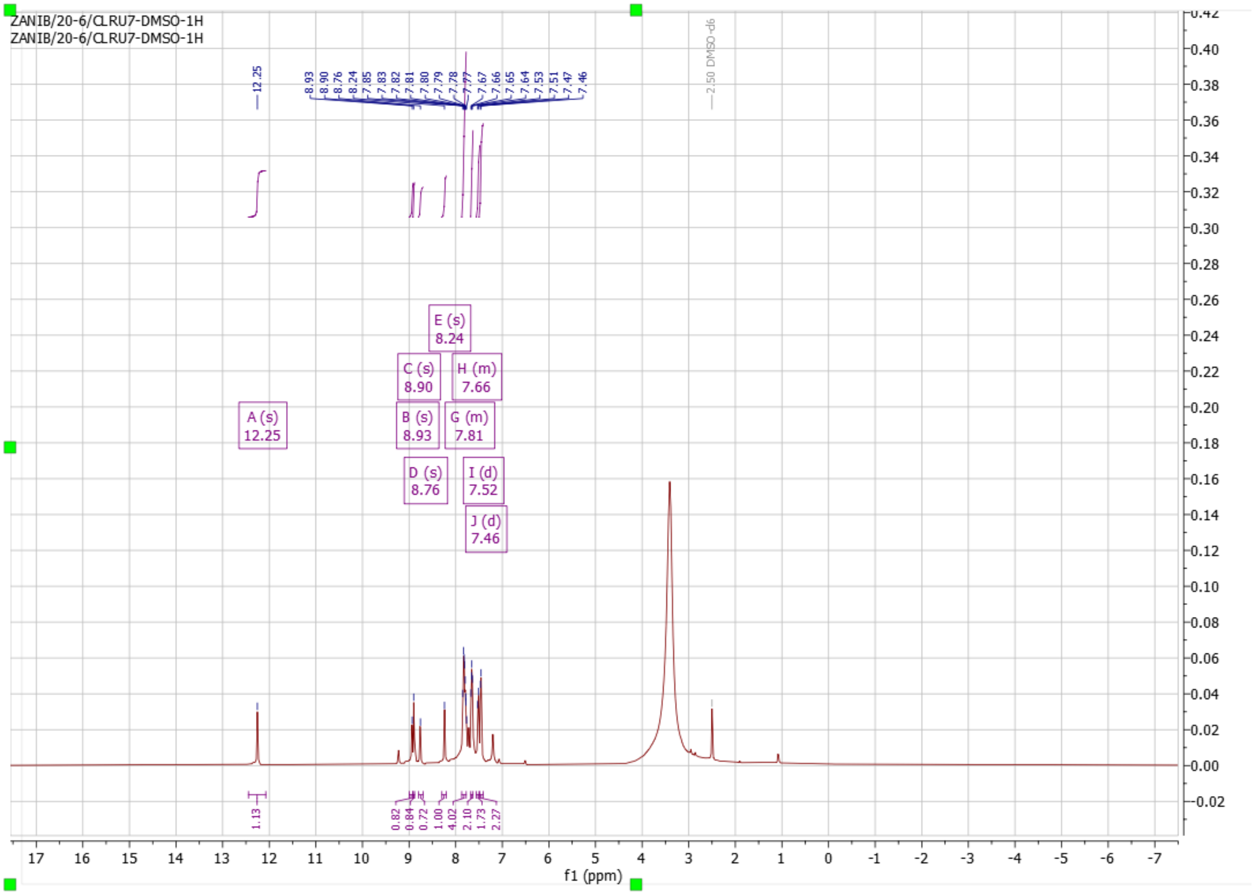

Fig S17: 1HNMR of **10g**

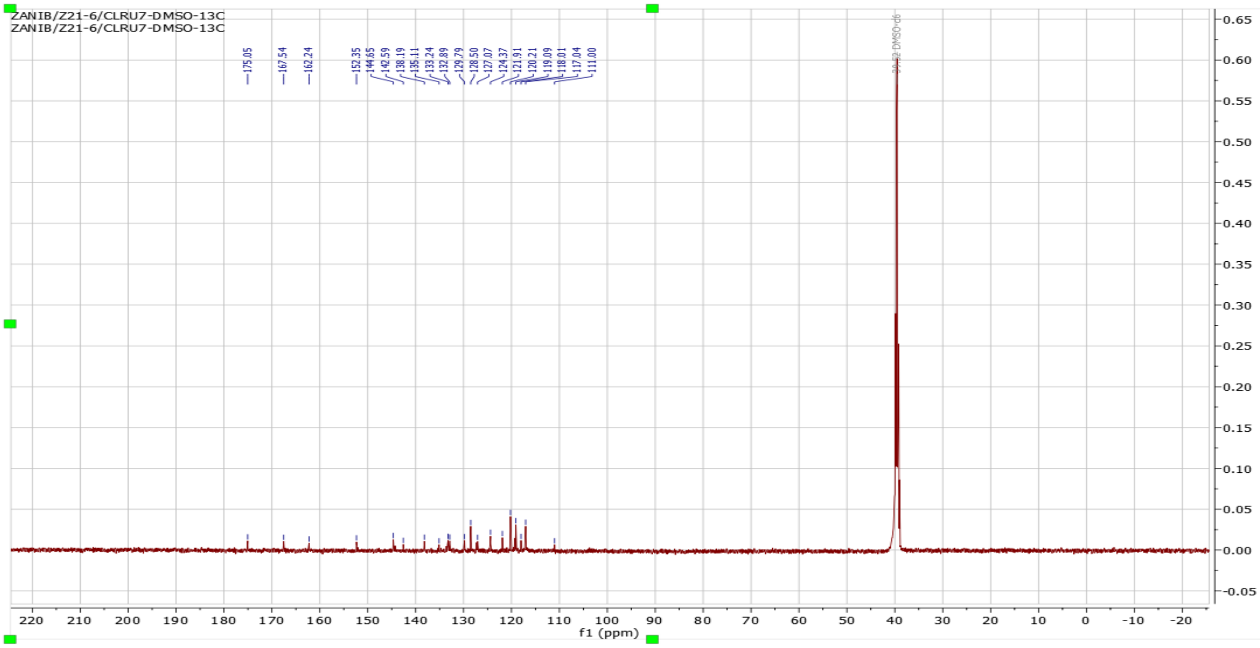

Fig S18: 13CNMR of **10g**

10h

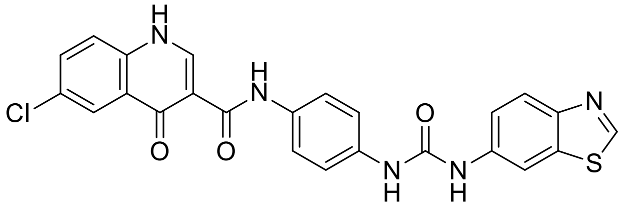

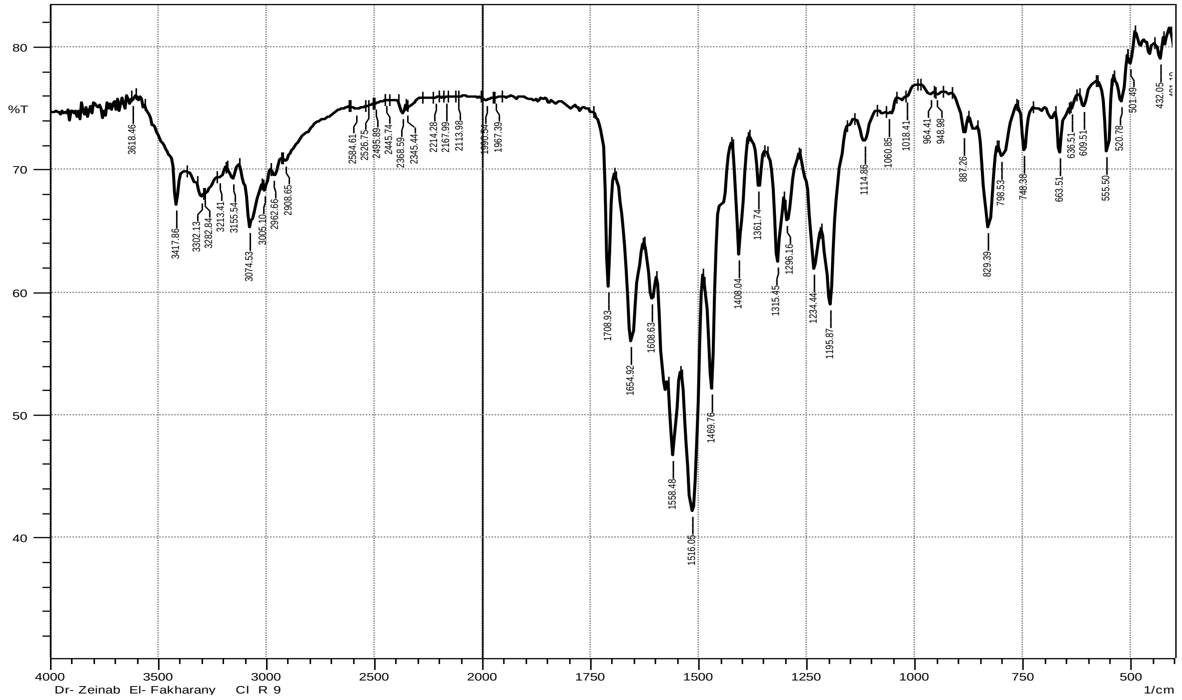

Fig S19: IR of **10h**

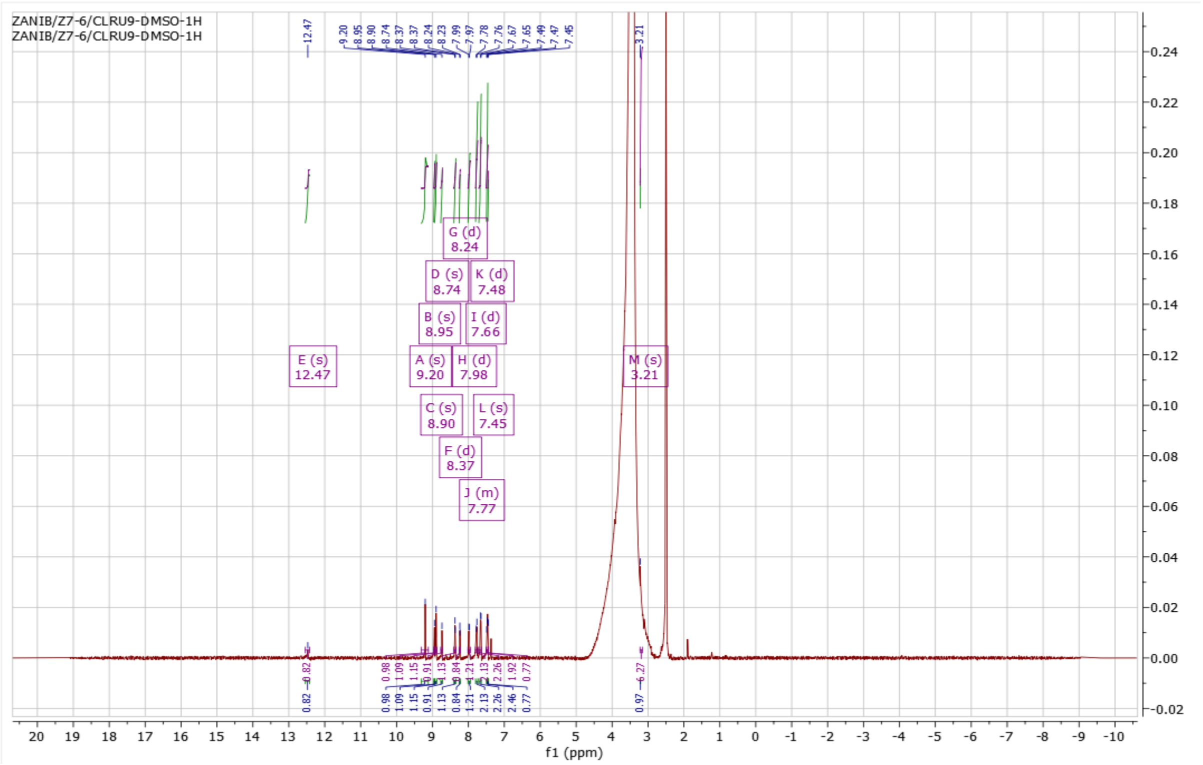

Fig S20: 1HNMR of **10h**

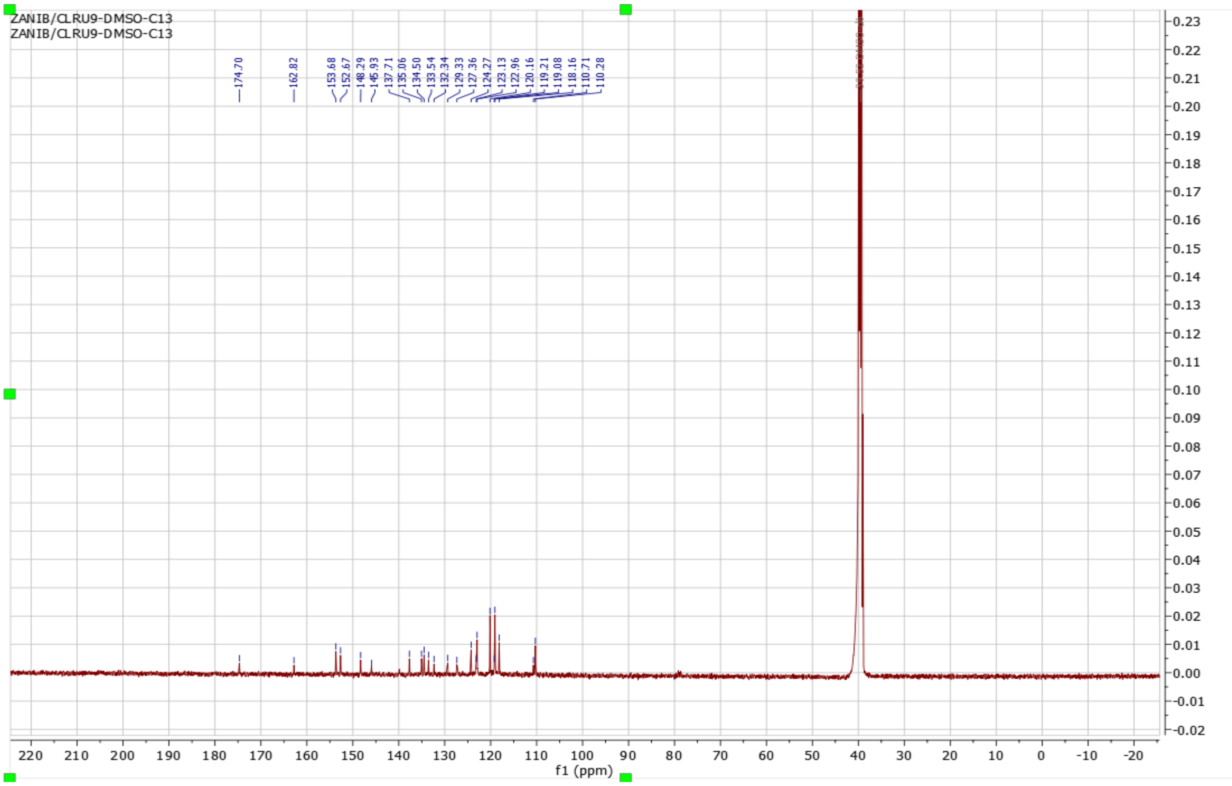

Fig S21: 13CNMR of **10h**

10i

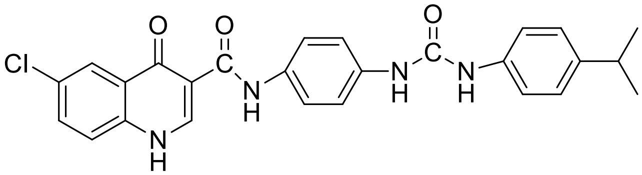

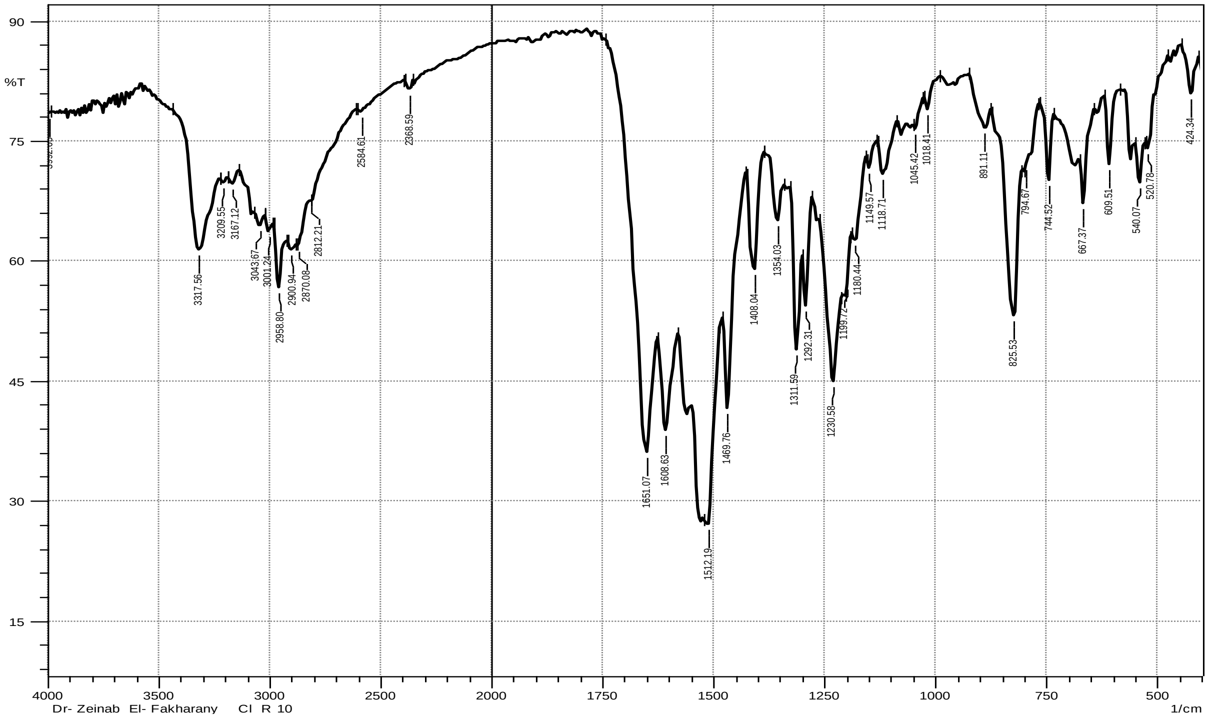

Fig S22: IR of **10i**

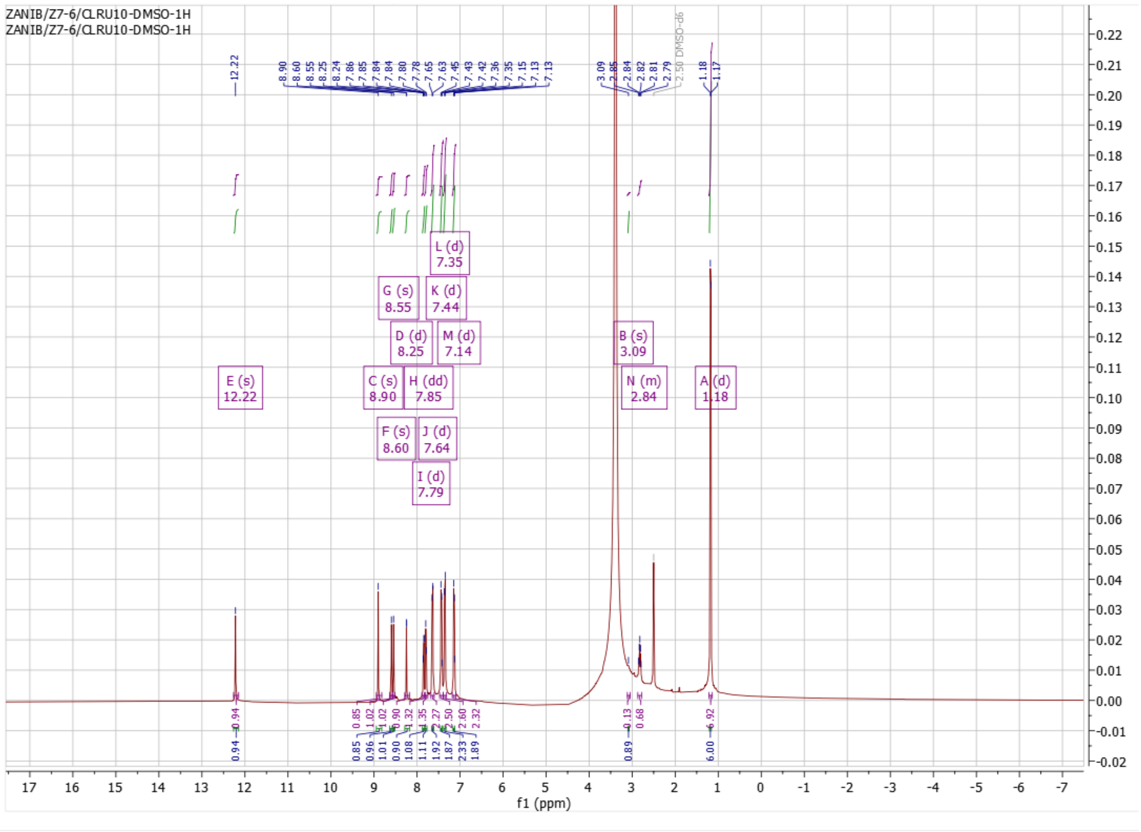

Fig S23: 1HNMR of **10i**

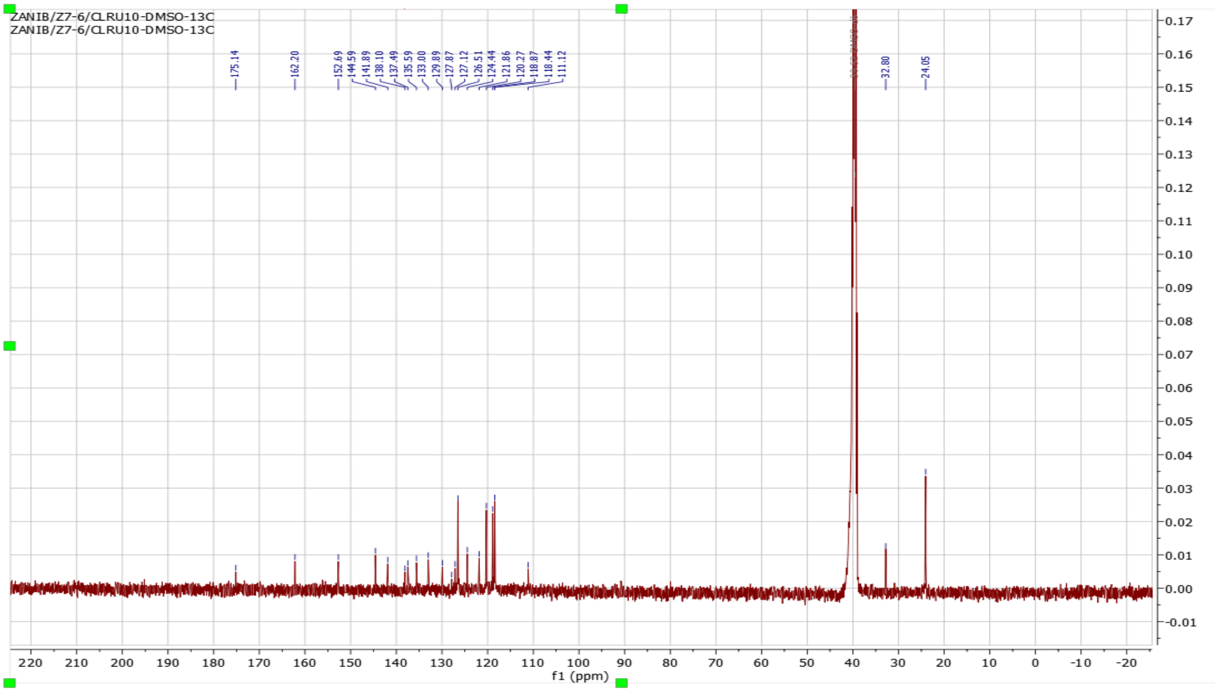

Fig S24: 13CNMR of **10i**

10j

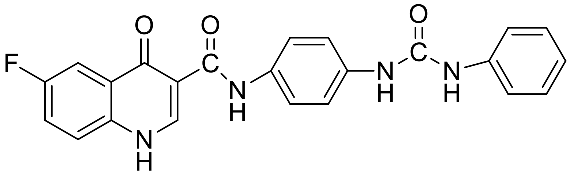

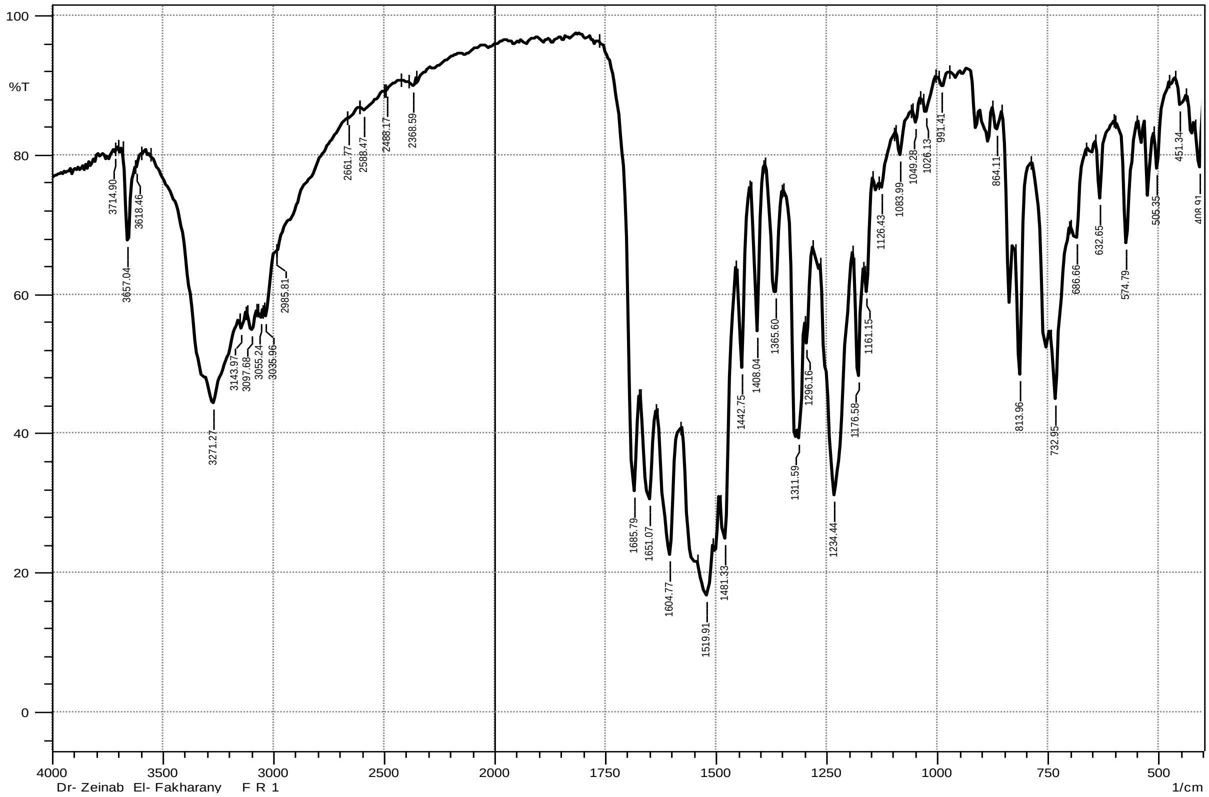

Fig S25: IR of **10j**

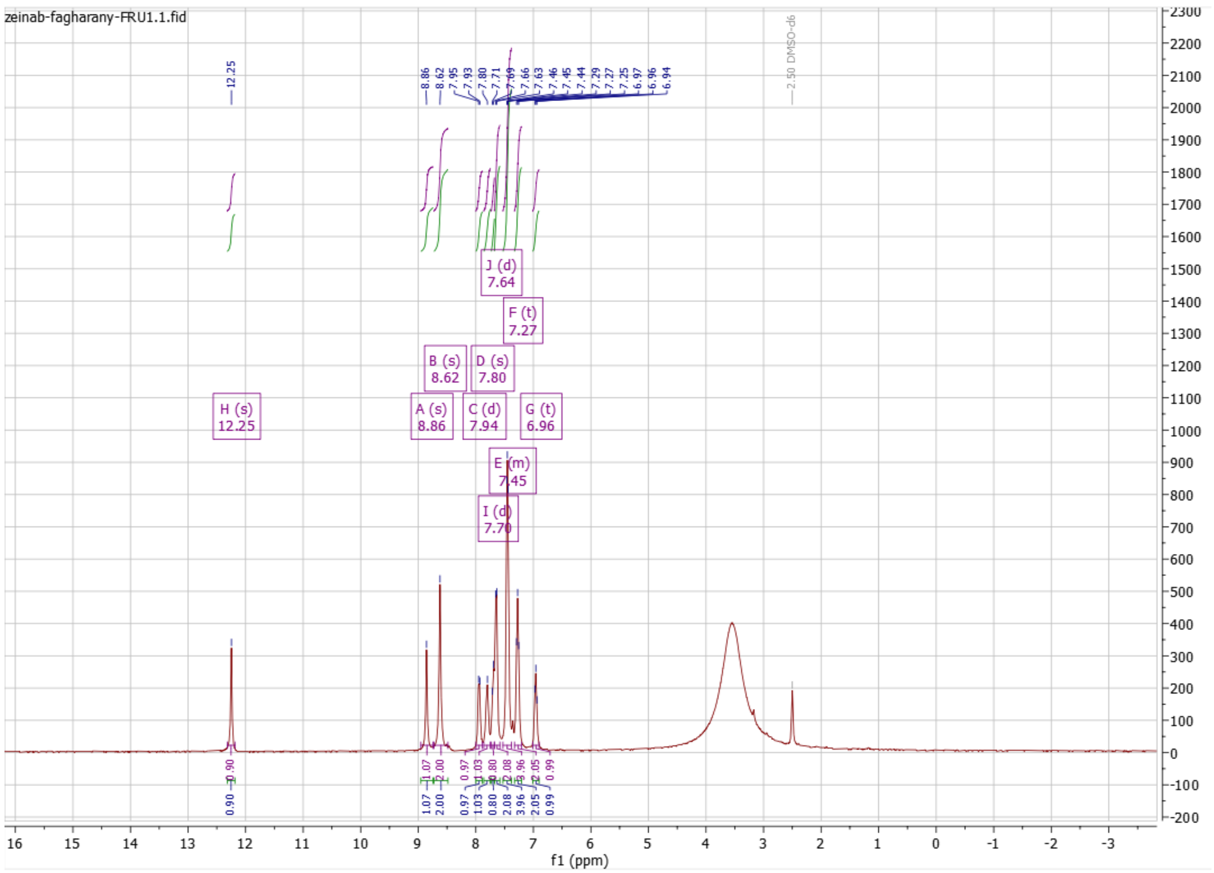

Fig S26: 1HNMR of **10j**

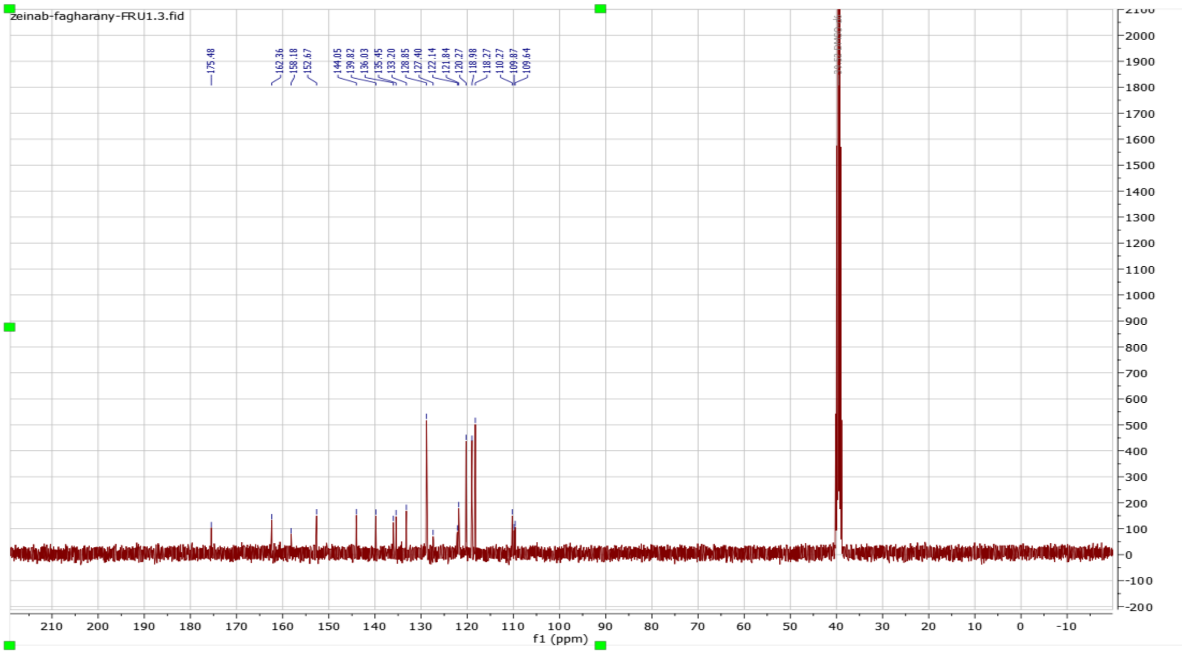

Fig S27: 13CNMR of **10j**

10k

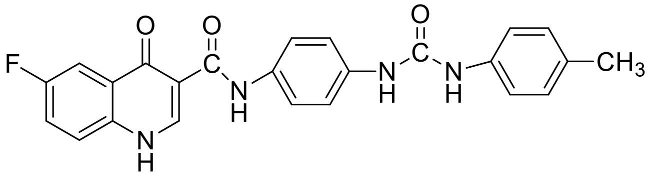

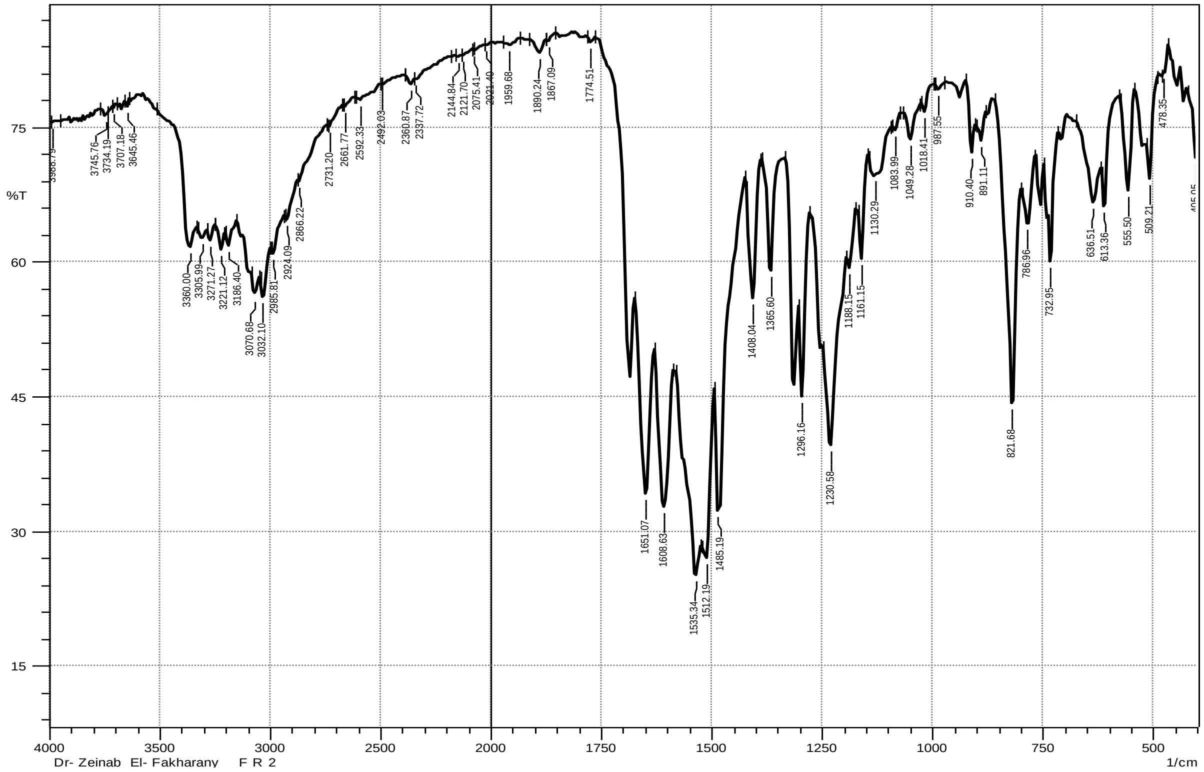

Fig S28: IR of **10k**

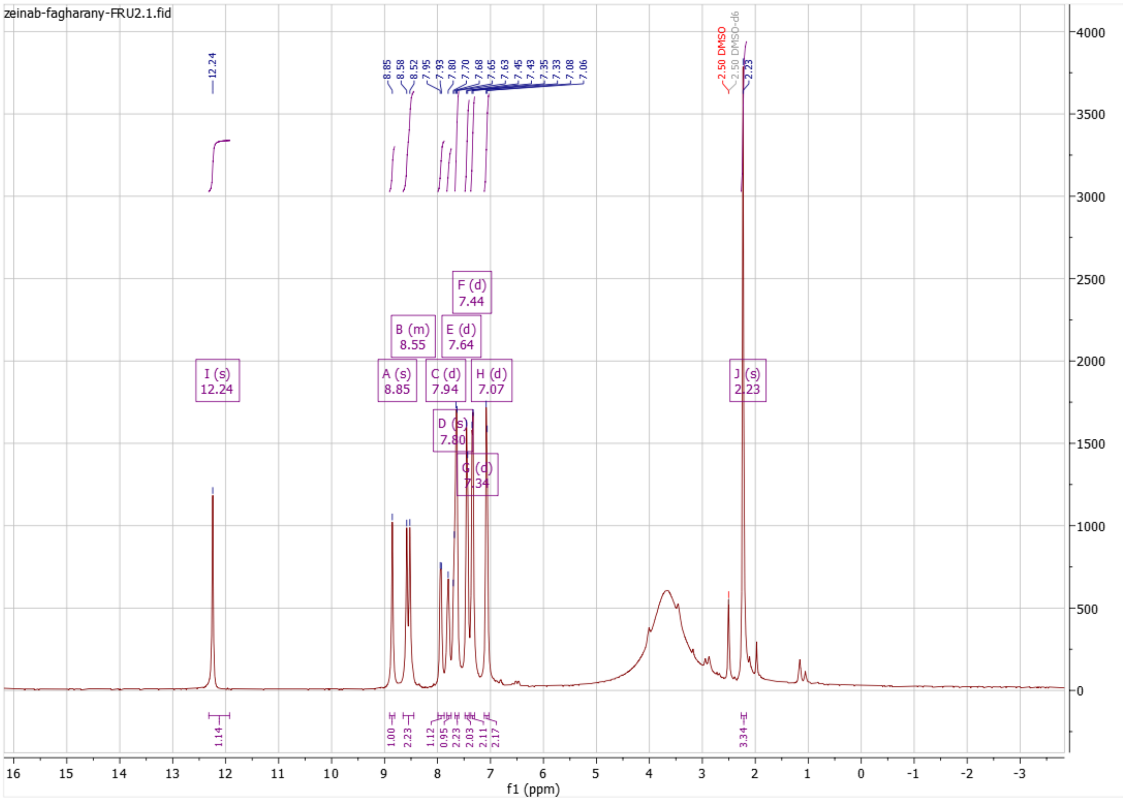

Fig S29: 1HNMR of **10k**

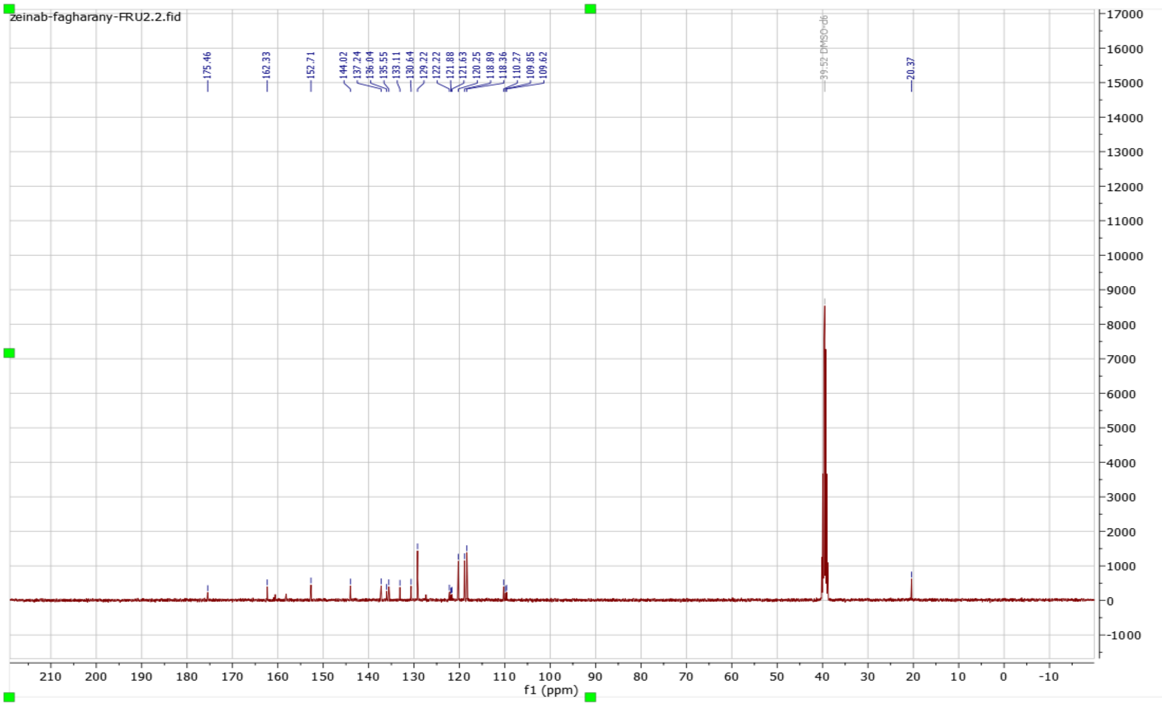

Fig S30: 13CNMR of **10k**

10l

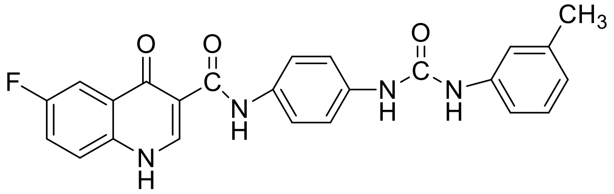

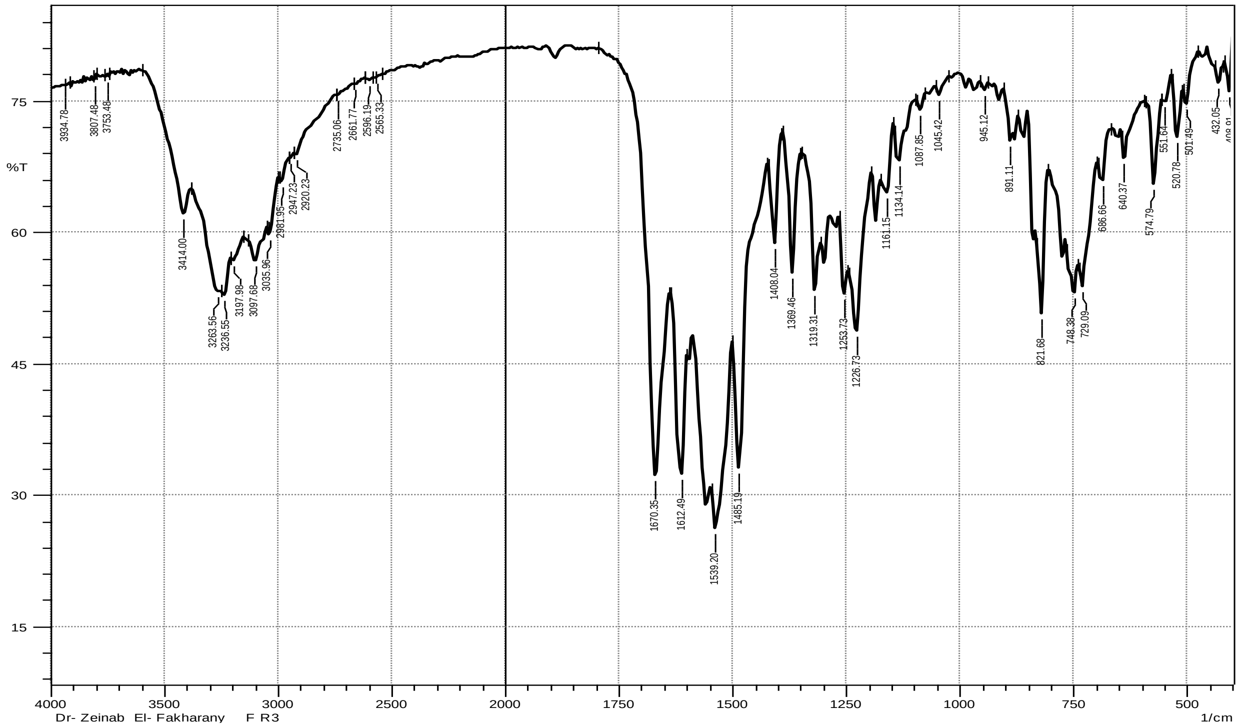

Fig S31: IR of **10l**

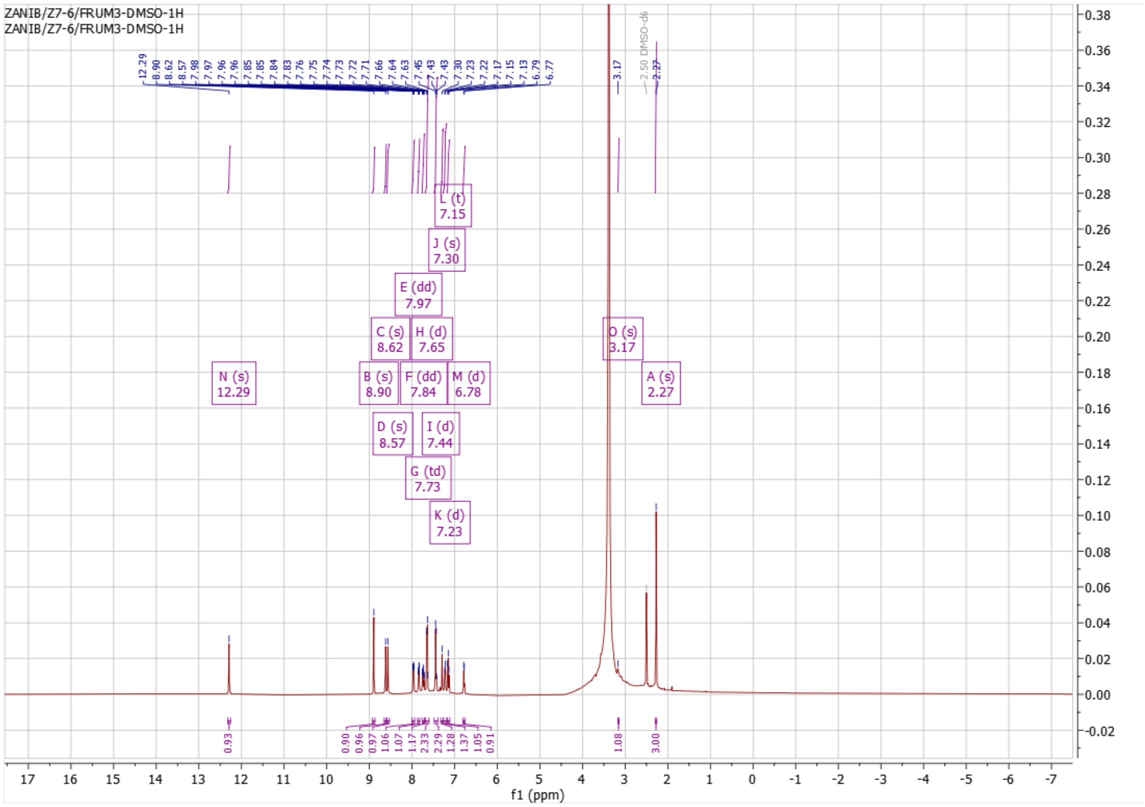

Fig S32: 1HNMR of **10l**

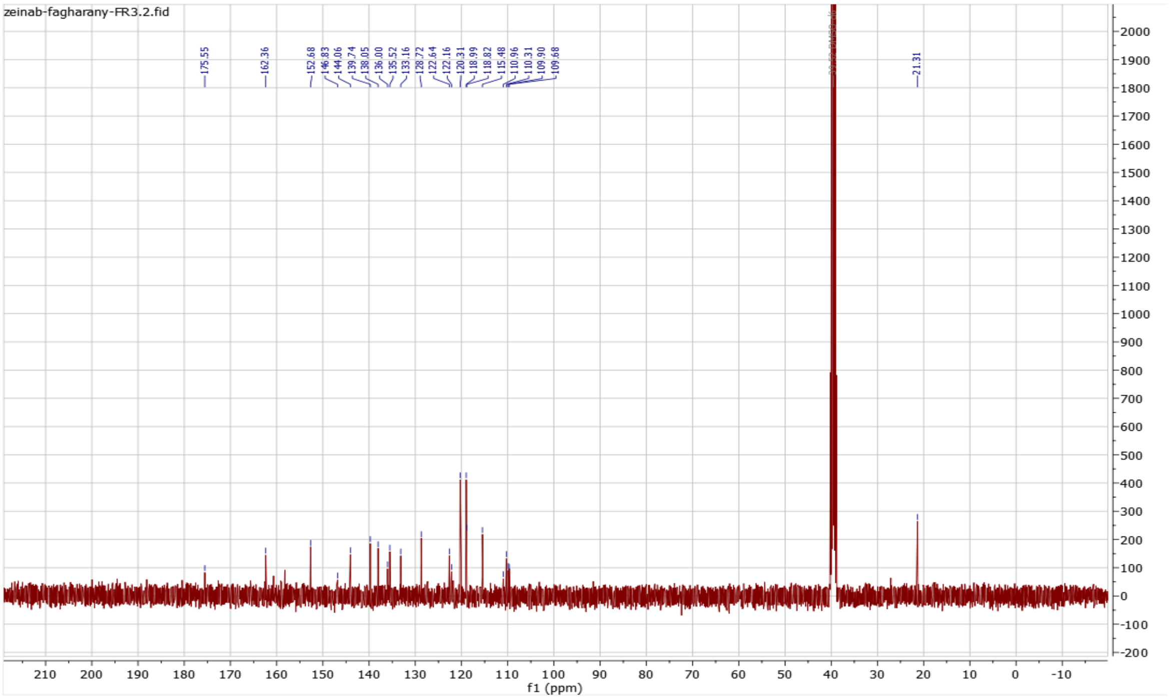

Fig S33: 13CNMR of **10l**

10m

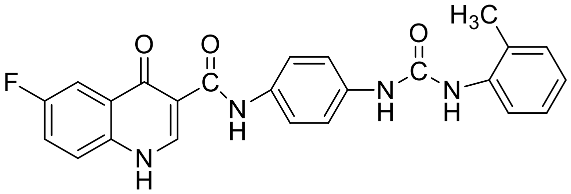

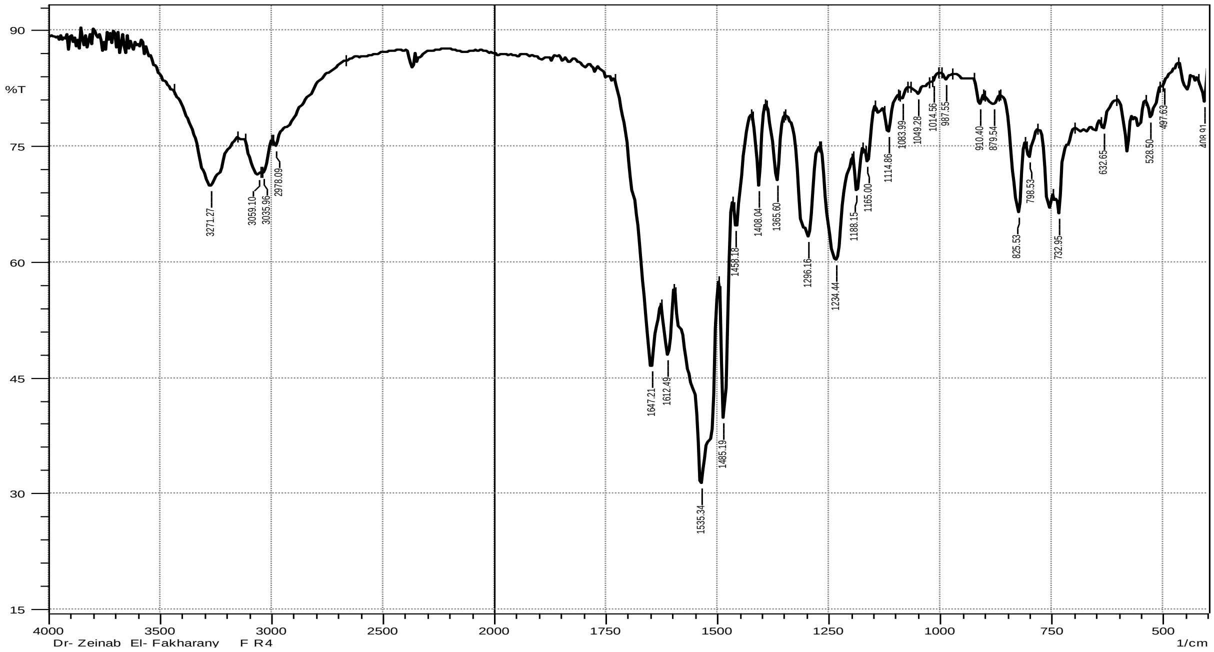

Fig S34: IR of **10m**

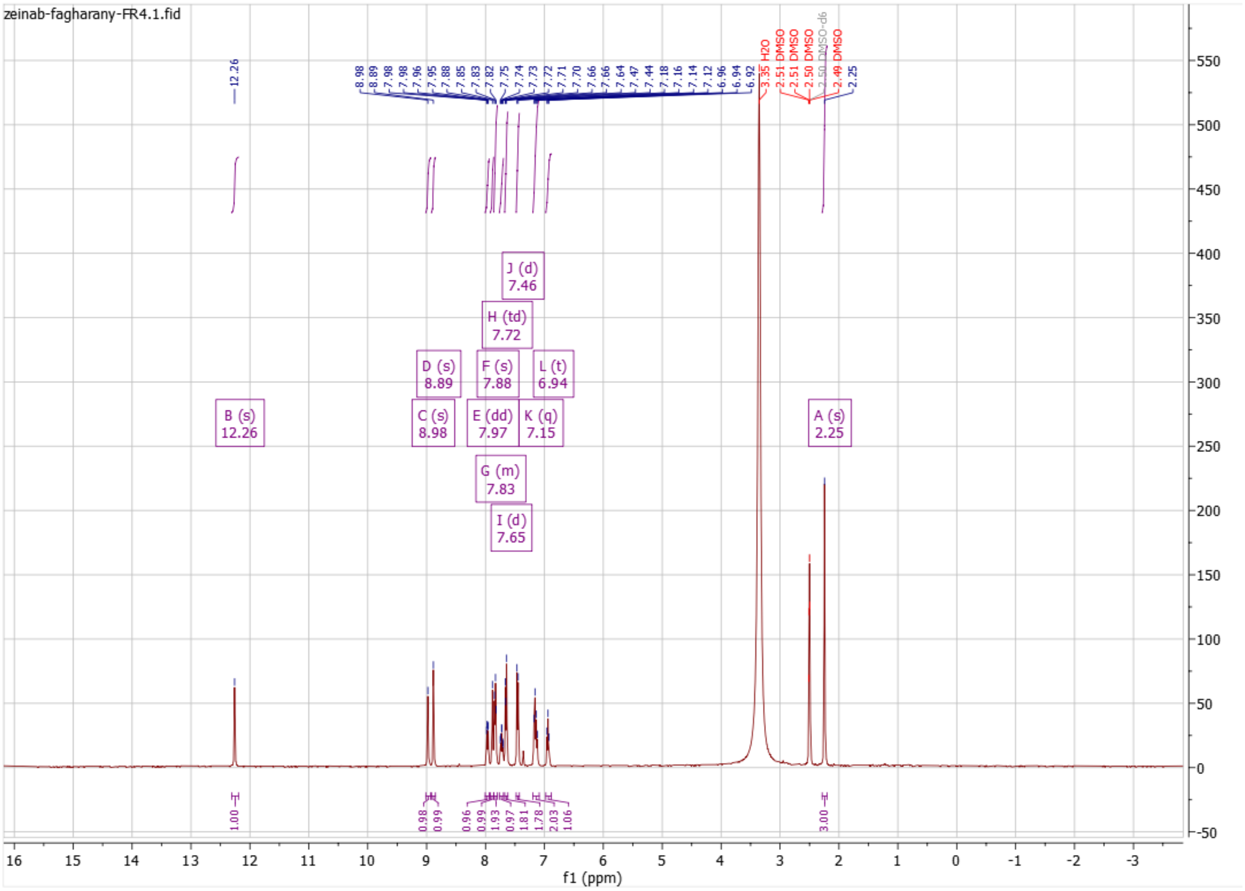

Fig S35: 1HNMR of **10m**

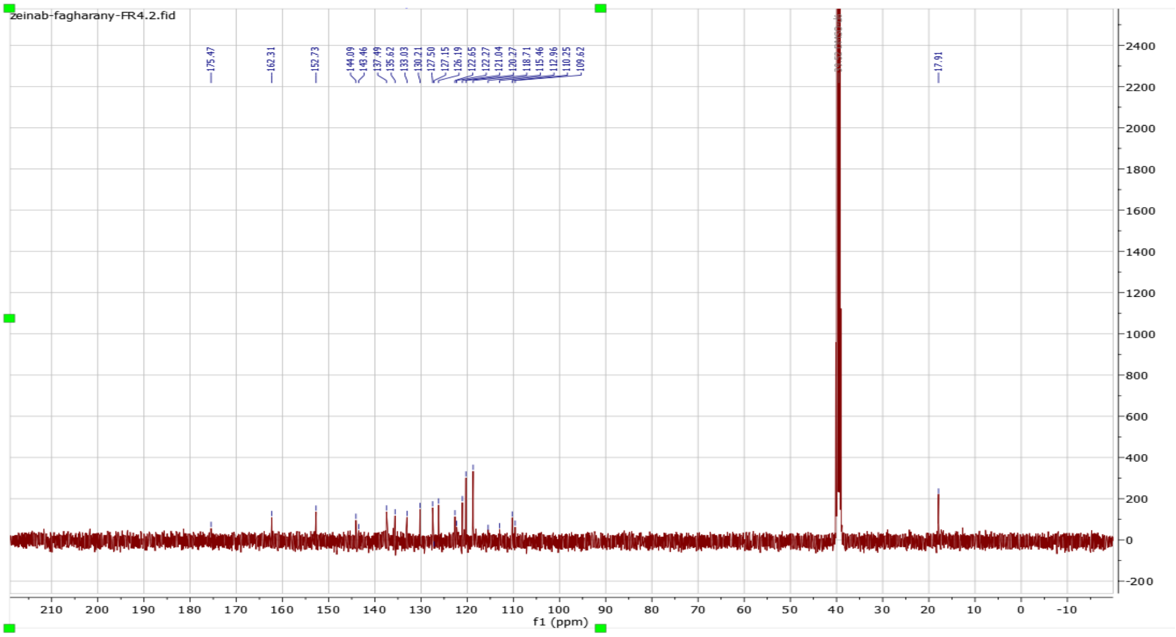

Fig S36: 13CNMR of **10m**

10n

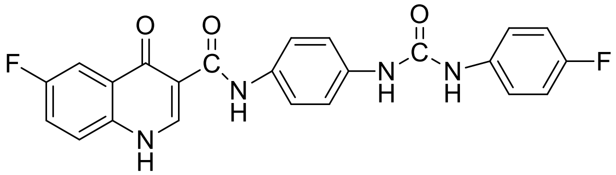

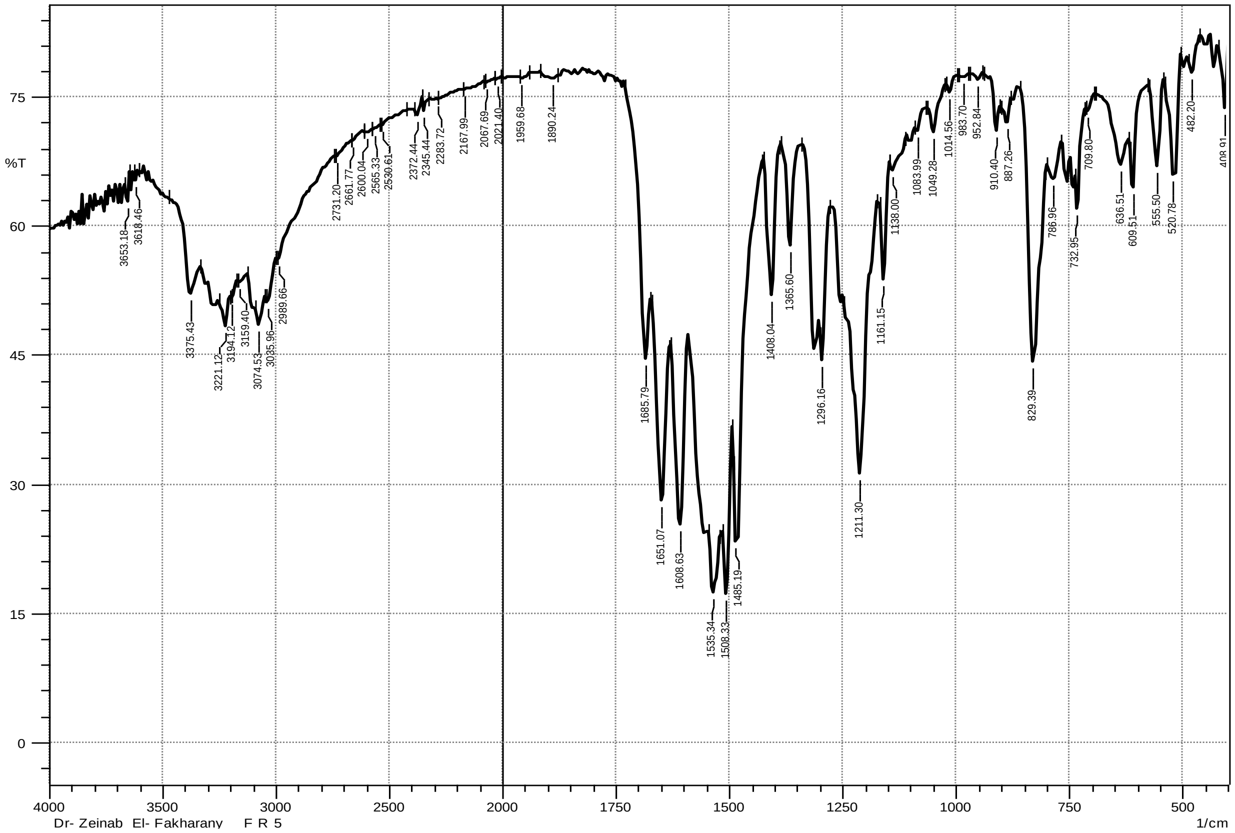

Fig S37: IR of **10n**

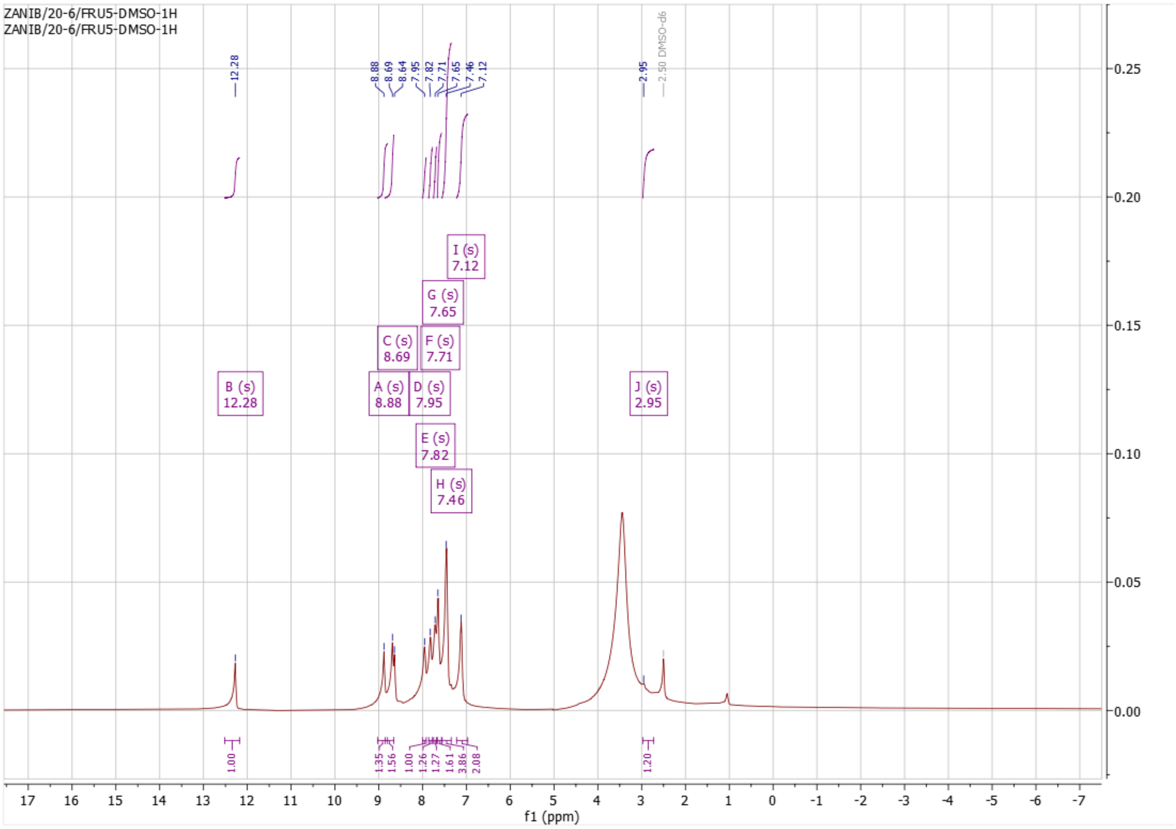

Fig S38: 1HNMR of **10n**

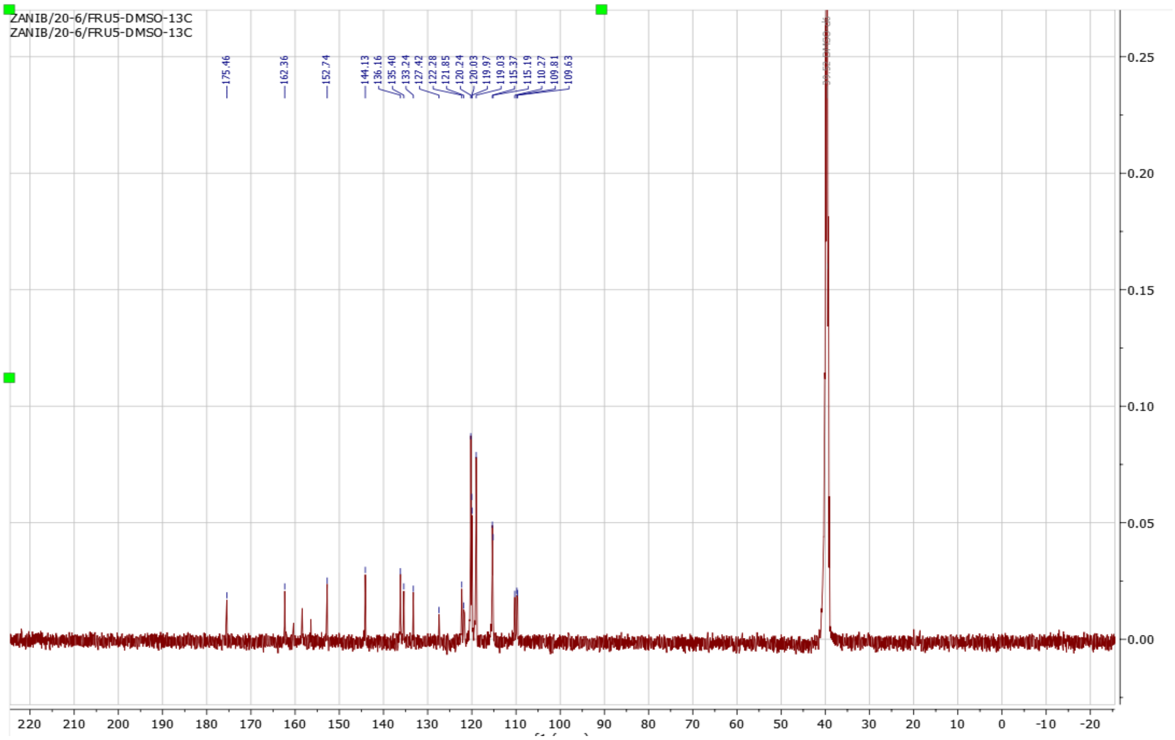

Fig S39: 13CNMR of **10n**

10o

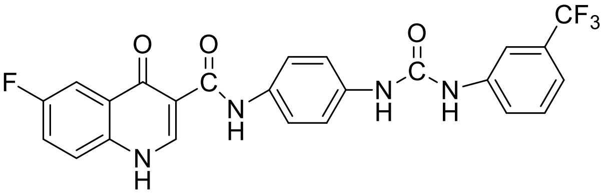

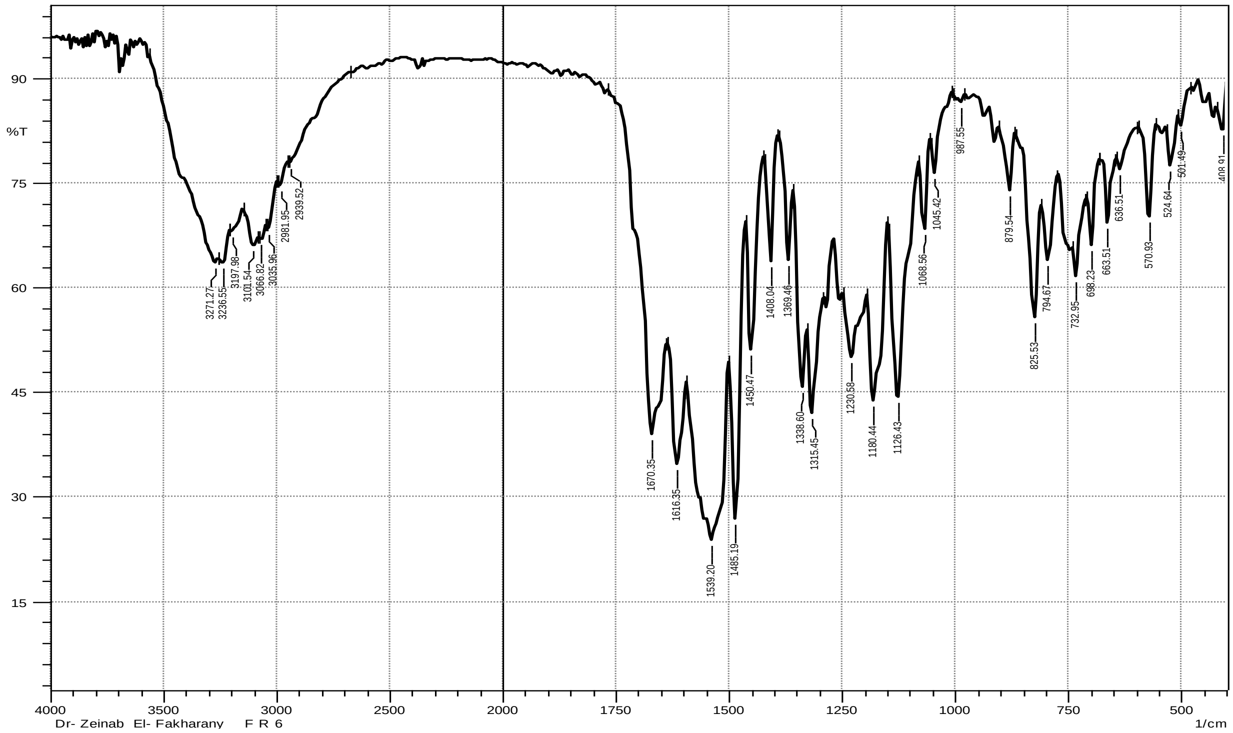

Fig S40: IR of **10o**

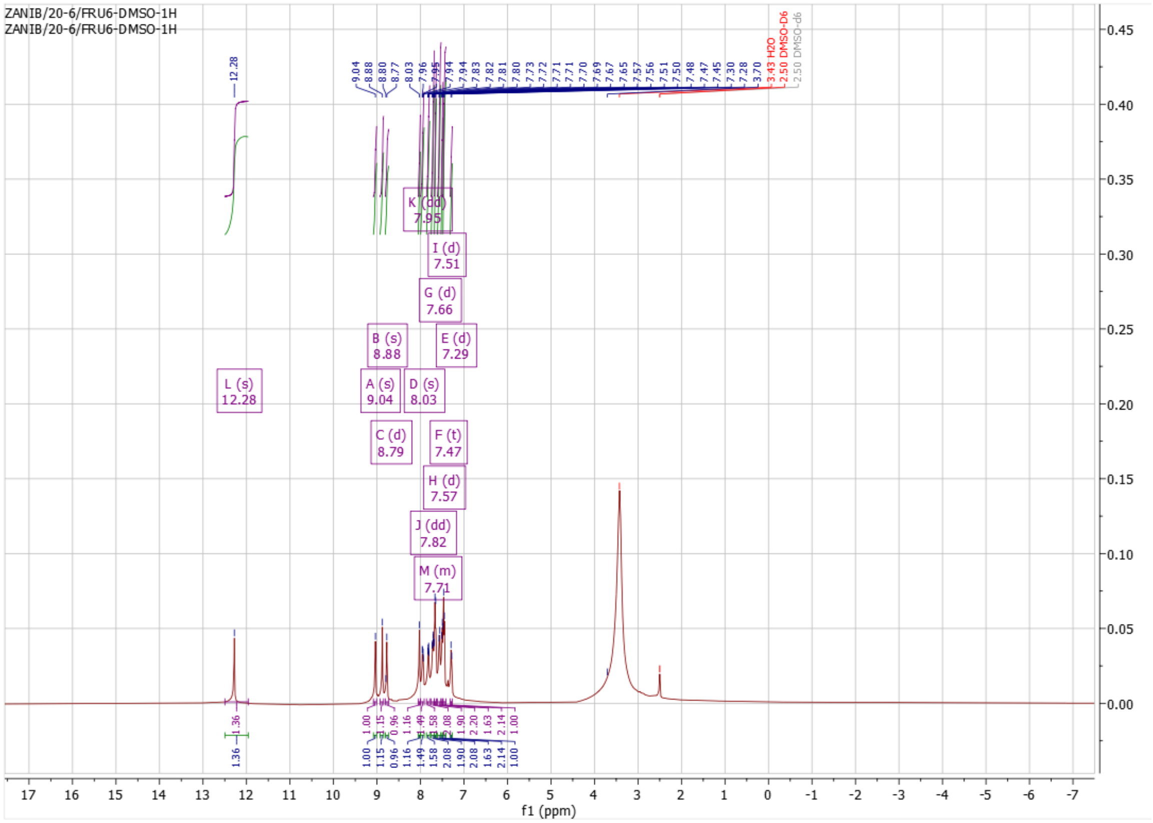

Fig S41: 1HNMR of **10o**

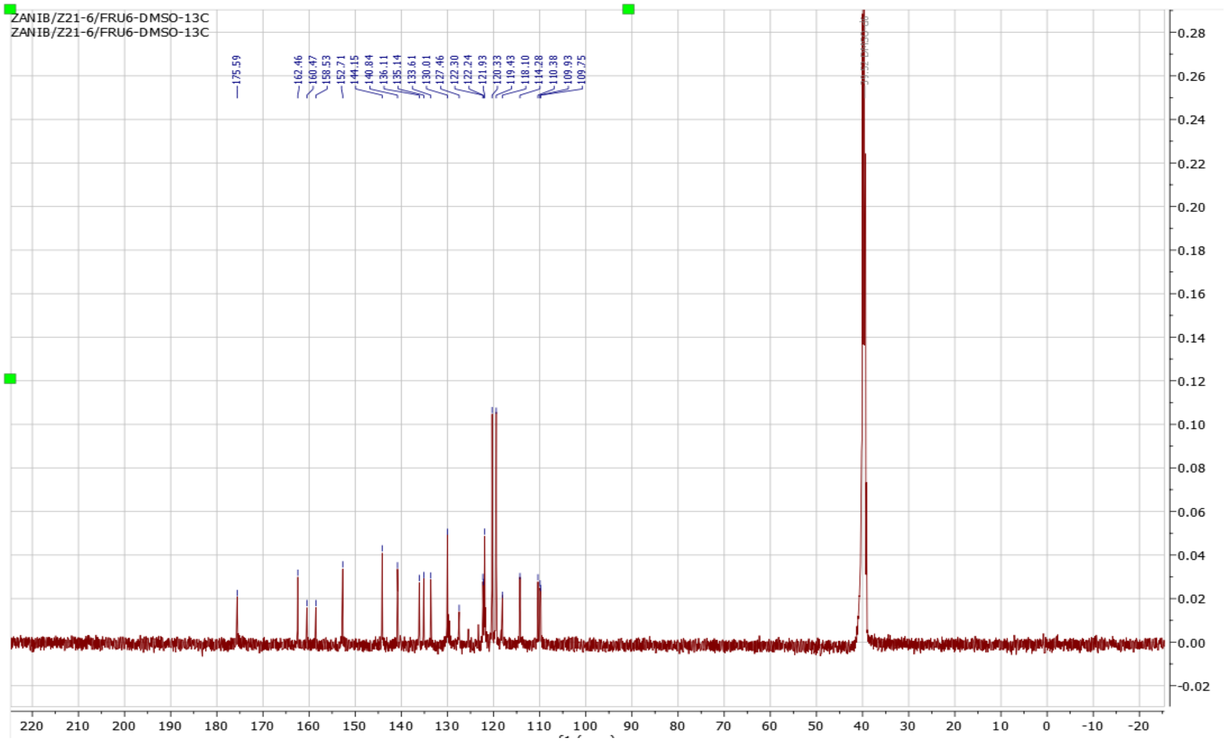

Fig S42: 13CNMR of **10o**

10p

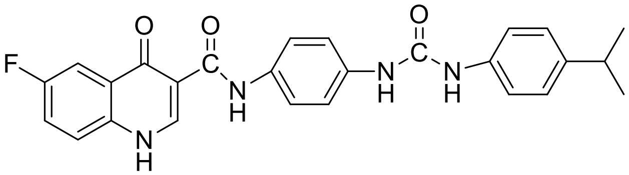

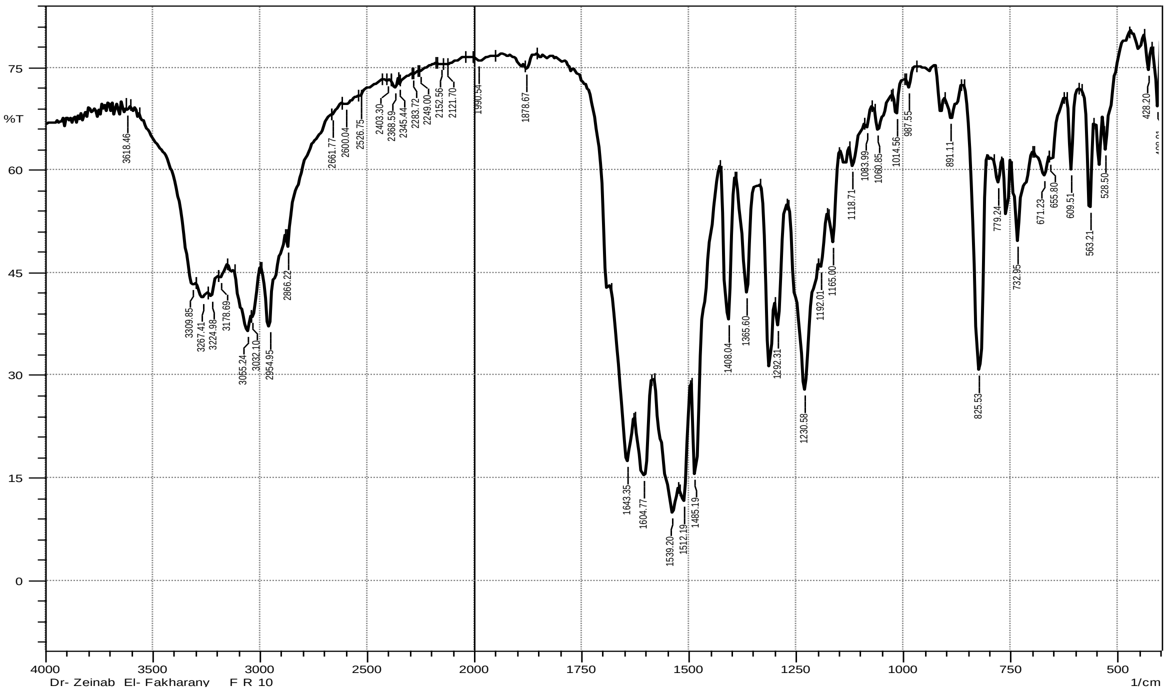

Fig S43: IR of **10p**

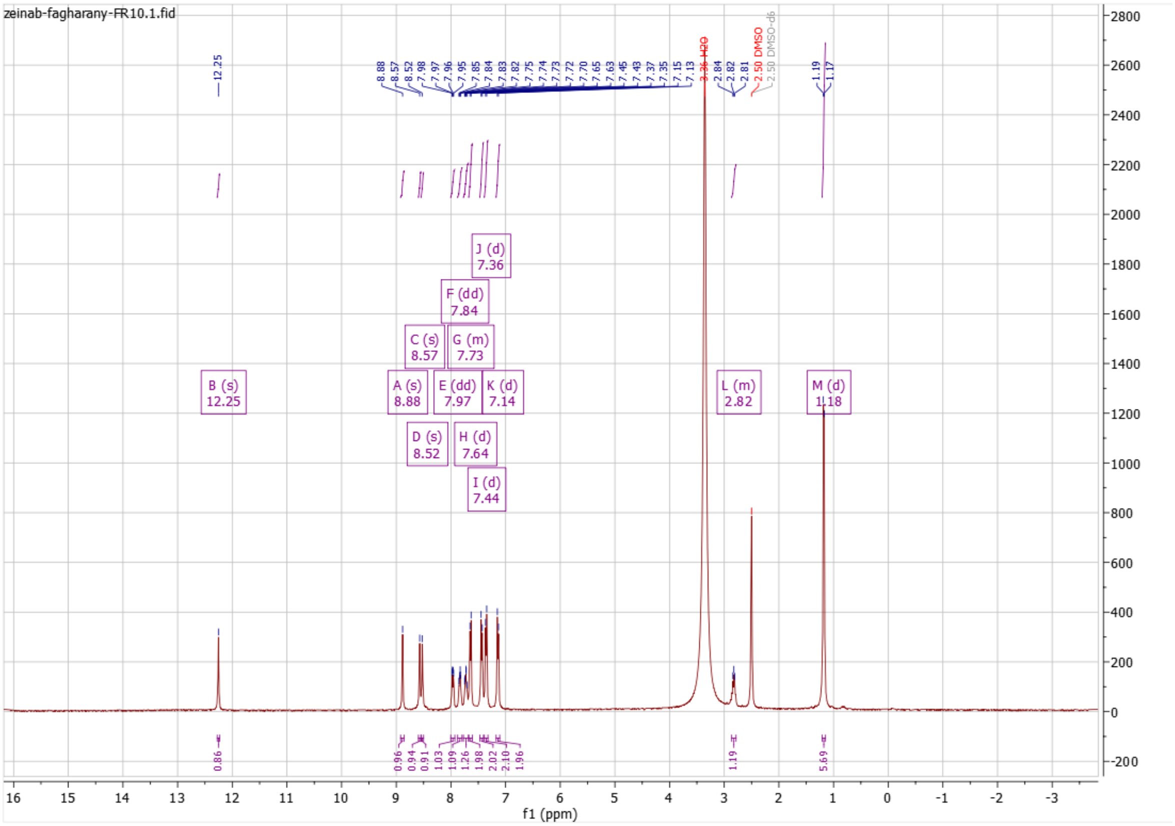

Fig S44: 1HNMR of **10p**

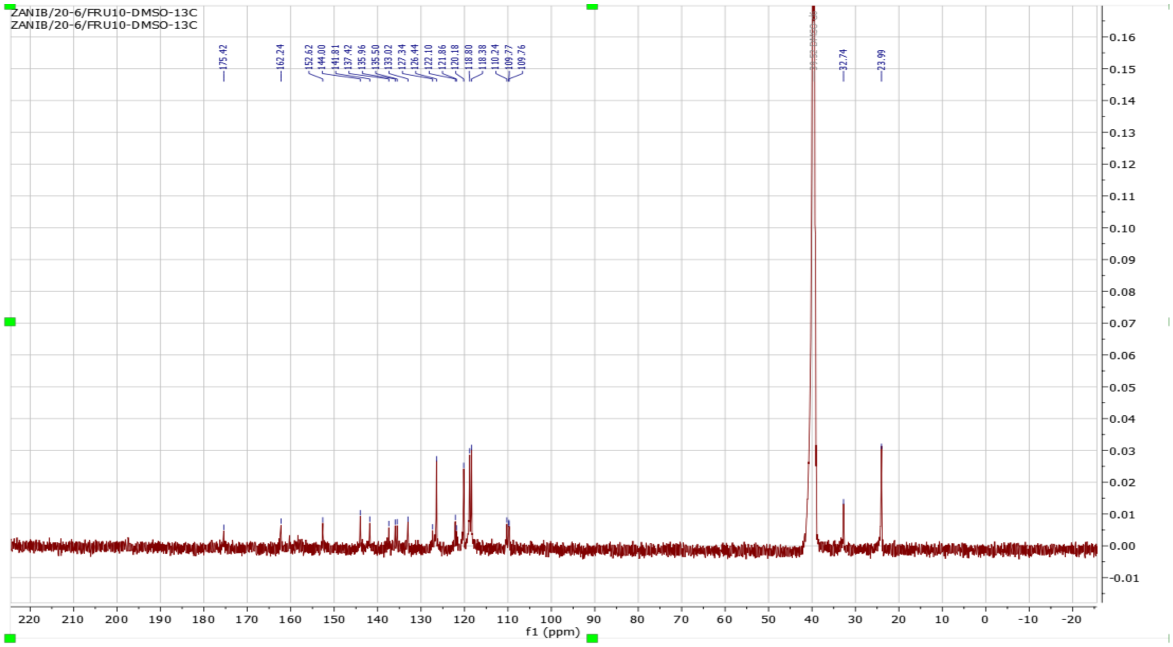

Fig S45: 13CNMR of **10p**
